# Prior-guided factorization for reliable imputation of scRNA-seq data

**DOI:** 10.1371/journal.pcbi.1014051

**Published:** 2026-03-20

**Authors:** You Wu, Li Xu, Ye Win Aung, Alex Michel Daoud

**Affiliations:** 1 College of Computer Science and Technology, Harbin Engineering University, Harbin, Heilongjiang, China; 2 National Engineering Laboratory for Modeling and Emulation in E-Government, Beijing, China; 3 Defense Services Medical Research Centre, Nay Pyi Taw, Myanmar; 4 Division of Neurosurgery, Department of Surgery, Irmandade da Santa Casa de Misericórdia de São Paulo, São Paulo, SP, Brazil; Wuhan University, CHINA

## Abstract

Single-cell RNA sequencing (scRNA-seq) provides an important means to reveal the heterogeneity and dynamic processes of tissues, organisms, and complex diseases, but technical capture loss (dropout) often obscures true biological expression, and existing imputation methods have difficulty distinguishing biological zeros (silent expression) from technical noise. To address this, we propose the imputation framework scZN. scZN assumes that the observed scRNA-seq data arise from a combination of RNA’s two-state transcription process and dropout, and formulates imputation as nonnegative factorization: decomposing the raw count matrix into two interpretable nonnegative factors, performing learning and optimization under constraints from prior knowledge and multiple regularizations, thereby reconstructing the cellular expression landscape. Experiments show that scZN can capture the true distributional characteristics at both the gene and cell levels and significantly suppress spurious activation of genes that should not be expressed. Across multiple real datasets, it outperforms dozens of state-of-the-art methods. Especially in complex experimental design scenarios, scZN markedly improves trajectory inference for embryonic stem cells and mouse dentate gyrus data. In Alzheimer’s disease data, scZN can also effectively recover pathways related to neuroinflammation, improving downstream scRNA-seq analysis. Overall, scZN provides a unified framework for missing-value imputation and expression reconstruction that combines accuracy and interpretability.

## Author summary

We aim to better understand true gene expression states in single-cell RNA sequencing data, where technical limitations introduce many zero values arising from both genuine gene silencing and missing signals due to insufficient capture efficiency. Distinguishing these two types of zeros is essential for revealing cellular heterogeneity but remains challenging for existing methods. Here, we present scZN, a single-cell data imputation framework based on a stochastic two-state model of RNA transcription that explicitly accounts for dropout. By formulating imputation as a biologically constrained nonnegative factorization problem, scZN recovers gene expression while maintaining interpretability. Across multiple real datasets, scZN more effectively suppresses spurious gene activation and improves downstream analyses such as developmental trajectory inference and pathway analysis, particularly in complex experimental designs and disease-related data, providing a unified and biologically meaningful solution for handling missing values in single-cell RNA sequencing.

## Introduction

Single-cell RNA sequencing (scRNA-seq) technology offers unprecedented resolution for studying individual cells [1], enabling comprehensive characterization of cellular heterogeneity [2], identification of novel cell types [3], reconstruction of complex differentiation and developmental trajectories [4], and deeper insights into human diseases [5]. However, despite continual improvements in sequencing methodologies, inherent technical limitations—such as amplification bias [6], cell cycle effects [7], and low RNA capture efficiency [8]—inevitably introduce significant noise into scRNA-seq experiments, resulting in count matrices replete with zeros. These zeros comprise both true zeros, which indicate genuine absence of gene expression reflective of cell-specific transcriptomes, and false zeros, which arise from technical noise (i.e., dropout events) [9]. The prevalence of false zeros undermines the integrity of the biological signal, thereby impeding downstream analyses [10].

To address this challenge, early approaches primarily comprised smoothing-based imputation strategies and statistical modeling methods, which leverage intercellular similarity and the underlying data structure [11–17]. Although smoothing-based methods perform well in reconstructing trajectories from time-series scRNA-seq data [18], most count matrices encountered in practice lack intrinsic temporal structure. These approaches can therefore induce substantial changes to expression values, potentially distorting the original gene-expression landscape. In contrast, methods that purely model data structure may preserve global patterns but often overlook biological context, imputing all zeros indiscriminately, which can overestimate performance and diminish biological interpretability.

In recent years, deep learning methods have increasingly been applied to scRNA-seq data imputation. DeepImpute [19] uses neural networks to impute missing values, but its approach of allocating 95% of the dataset for training may lead to overfitting. AutoImpute [20] combines autoencoders with the inherent data distribution for imputation, yet its tendency to over-impute in pursuit of optimal results often produces a substantial number of invalid (negative) values. DCA [21] enhances scRNA-seq imputation by integrating the negative binomial distribution, the zero-inflated negative binomial distribution, and autoencoders. scVI [22,23], a hierarchical Bayesian model, projects the data into a latent space for imputation. However, it struggles to handle cases where cell counts exceed gene counts. DISC [24] treats unexpressed genes as trainable parameters to improve the model’s resistance to overfitting and enhance imputation reliability, although it imposes significant computational demands on large datasets. sciGANs [25] redefines the imputation task as an image restoration problem by converting cellular gene expression into a square matrix using GAN-based methods, which introduces considerable noise. In our previous work [26], we further improved scIGANs using masking and attention mechanisms, developing the scMASKGAN algorithm that achieved higher imputation performance. scGNN [27] leverages graph learning to model the topology of scRNA-seq data, but the resulting topology can greatly affect the expression of marker genes. Moreover, although many other deep learning models have been developed [28–30], they often pay limited attention to the biological significance of imputation, and relying solely on clustering metrics prevents their application to downstream analyses.

To address these challenges, we propose scZN. The framework starts from the physical processes of RNA transcription and capture dropout in single-cell sequencing and explicitly models gene-level overdispersion and zero inflation. scZN assumes that each cell’s transcriptome is a nonnegative additive mixture of regulatory modules. Leveraging nonnegative matrix factorization (NMF) [31], we decompose the global count matrix into a soft assignment matrix mapping cells to cell types and a cell-type gene expression matrix. Building on this, we inject prior biological knowledge into the factor matrices via a simple linear shrinkage scheme, thereby improving interpretability and suppressing biologically implausible activations, which in turn reduces spurious signals during imputation. The entire model is trained end-to-end with multiple regularizers that jointly reconstruct scRNA-seq data, yielding efficient and accurate gene-expression estimation and dropout imputation.

## Results

### Overview of scZN

**Fig 1:**
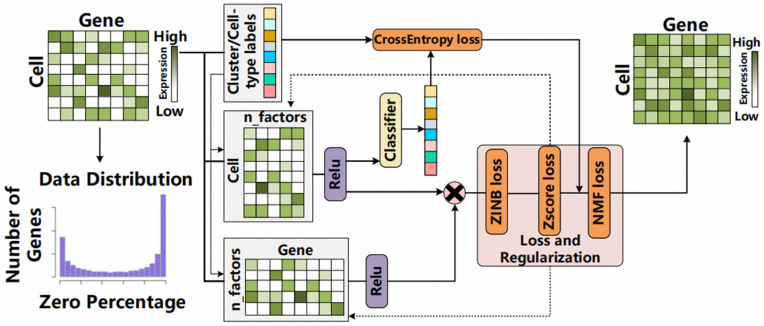
The framework of scZN.

We propose a single-cell RNA sequencing (scRNA-seq) imputation framework, scZN, which supports prior-guided supervised imputation as well as fully unsupervised imputation. Specifically, scZN assumes that transcriptional bursting follows a Gamma–Poisson (negative binomial) process and that technical dropout introduces excessive zeros, and it explicitly uses a zero-inflated negative binomial (ZINB) to model these two sources of sparsity. To mitigate the non-convexity of factorization, scZN linearly injects biological structure—such as lineage features and cell types—as priors into the decomposition process. scZN is implemented end-to-end in PyTorch and optimized using the Adam optimizer together with multiple regularization strategies. (see Fig 1).

### Performance benchmarking

To improve the reliability and accuracy of downstream analyses and biological discovery, the core objective of scRNA-seq imputation is to correct technical noise. Accordingly, we performed a systematic benchmark across multiple real datasets (see Methods) with cell-type labels. In the raw data, pervasive dropout often manifests as cluster intermixing in dimensionality-reduced embeddings such as UMAP, an effective imputation method should attenuate this technical artifact, enhance the resolution of cell-population heterogeneity, and yield consistent improvements in clustering metrics. Using these criteria, we quantitatively evaluated the imputation performance of scZN and compared it objectively against baseline methods.

**Fig 2:**
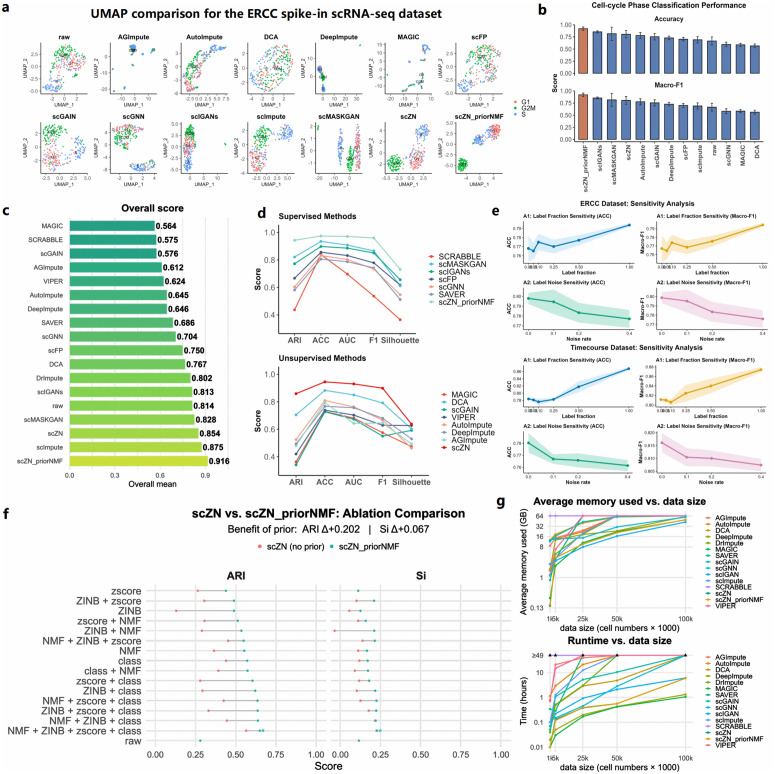
Benchmarking of imputation methods. **(a)** UMAP projections of the ERCC spike-in scRNA-seq dataset comparing the raw data with imputed results from 13 methods, including AGImpute, AutoImpute, DCA, DeepImpute, MAGIC, scFP, scGAIN, scGNN, scIGANs, scImpute, scMASKGAN, scZN, and scZN_priorNMF. **(b)** Cell-cycle phase classification performance on the ERCC spike-in dataset, evaluated using Accuracy (ACC) and Macro-F1 score. **(c)** Bar plot showing the overall performance score, computed as the average across all evaluation metrics. **(d)** External consistency evaluation on the D1 datasets, where all baseline methods are grouped into supervised and unsupervised categories. **(e)** Comparison of memory consumption and runtime between scZN and all baseline methods across datasets of different sizes. **(f)** Ablation study evaluating different combinations of regularization hyperparameters in scZN (red points) and scZN_priorNMF (blue points), illustrating the effect of prior injection on ARI and silhouette coefficient. The blue star marks the hyperparameter configuration achieving the best performance. **(g)** Robustness analysis of scZN_priorNMF with respect to different qualities of prior labels. **(h)** Label sensitivity analysis during the imputation process on Time-course datasets, shaded regions around the curves indicate variability.

Using the D1 dataset (see Methods), we systematically compared 14 baseline imputation algorithms. We first performed UMAP visualization to examine whether each method could recover cellular heterogeneity. Fig 2a shows the UMAP embeddings of the ERCC spike-in dataset [32] processed by different methods. Compared with the raw data, scZN and scZN_priorNMF exhibit clearer cluster separation. In contrast, several methods (e.g., DrImpute, SAVER, VIPER, and SCRABBLE) generate an imputed number of cells exceeding the size of the ground-truth label set, introducing spurious structures. As a result, UMAP embeddings and external consistency metrics cannot be computed in these cases.

However, we observed that the circular structure corresponding to cell-cycle stages appeared to be partially disrupted in the UMAP embeddings. We therefore further evaluated the accuracy of cell-cycle phase classification on the ERCC spike-in dataset. Specifically, we treated cell-cycle phase prediction as a supervised multi-class classification task, using the imputed expression profiles produced by each method as input features and the provided phase labels as ground truth. For each imputation method, we trained a multinomial logistic regression classifier using standardized features. Performance was evaluated using 5-fold stratified cross-validation, in which cells were randomly split into five folds with preserved phase proportions. In each fold, the classifier was trained on 80% of the cells and tested on the remaining 20%. The results indicate that, among all compared methods, scZN_priorNMF consistently achieves the highest classification accuracy and macro-F1 score (Fig 2b). This demonstrates that, although the circular pattern is less apparent in low-dimensional embeddings, scZN_priorNMF enhances the discriminative information of cell-cycle phases in a quantitative sense.

To more rigorously assess imputation quality, we applied *k*-NN clustering to the imputed matrices and evaluated external consistency metrics across all datasets (see S1 Table). The results show that only scMASKGAN, scImpute, scZN, and scZN_priorNMF achieve overall improvements compared with the raw data, whereas the average performance of the other methods decreases (Fig 2c), indicating that most imputation strategies do not consistently improve data quality across datasets. Among them, scZN_priorNMF achieves the largest improvement across all datasets and all metrics. We also provide box plots of the evaluation metrics across all datasets in S1 Fig. These box plots summarize the performance distributions of all methods across datasets and clearly demonstrate the robustness and stability of scZN_priorNMF, which consistently achieves strong and stable performance across multiple evaluation metrics.

Notably, the imputation methods compared in this study include both unsupervised and supervised approaches. The unsupervised methods include AGImpute [33], MAGIC, DCA, scGAIN, VIPER, AutoImpute, DeepImpute, and scZN, whereas the supervised methods include SCRABBL, scMASKGAN, scIGANs, scFP, scGNN, SAVER, and scZN_priorNMF. The results show that, among unsupervised and supervised methods, scZN and scZN_priorNMF achieve the best overall performance across the evaluated metrics, respectively (Fig 2d).

Finally, in a hybrid computing environment with two vGPUs (32 GB) and a 13th-generation i9 CPU, we systematically quantified the runtime and memory usage of 15 algorithms across single-cell matrices of varying sizes. The results (Fig 2e) show that scZN and scZN_priorNMF both rank within the overall top three, exhibiting excellent computational efficiency and memory economy while maintaining leading imputation accuracy, making them suitable for routine and high-throughput processing of large-scale single-cell datasets. The dataset used for this evaluation was randomly generated.

### Hyperparameter ablation experiment

scZN incorporates multiple regularization techniques to ensure that the generated data are biologically meaningful, specifically including nonnegative matrix factorization(NMF) reconstruction, zero-inflated negative binomial (ZINB) [34] negative log-likelihood, z-score [35] regularization, and cell-type classification (see section Methods). Each regularization term has its own hyperparameter. The specific hyperparameters are described as follows: **Loss weight coefficients** $\lambda_{\text{NMF}}\;$, $\lambda_{\text{ZINB}}\;$, $\lambda_{\text{zscore}}\;$, and $\lambda_{\text{class}}\;$ are binary switches in {0,1} :


$$\begin{array}{c} \lambda =\left\{ \begin{array}{cc} 1, & \text{enable the corresponding loss term},\\ 0, & \text{disable the corresponding loss term}. \end{array}\right.\; \end{array}$$

We evaluated all 16 subsets of $\{\lambda_{NMF},\lambda_{ZINB},\lambda_{zscore},\lambda_{class}\}\;$. For each configuration, the model was trained for a fixed number of epochs on the ERCC spike-in scRNA-seq data, and performance on the held-out data was assessed using the ARI and average silhouette coefficient (Si). As shown in Fig 2f, activating a single loss term resulted in moderate performance improvements—with ${ℒ}_{class}\;$ alone achieving the highest ARI of 0.572—while certain pairs and triplets demonstrated clear synergistic effects (e.g., ${ℒ}_{ZINB}+{ℒ}_{zscore}+{ℒ}_{class}\;$ reached an ARI of 0.637). Notably, the full four-term combination ($\lambda_{NMF}=\lambda_{ZINB}=\lambda_{zscore}=\lambda_{class}=1\;$) stood out, achieving the highest ARI of 0.650 and the highest silhouette coefficient of 0.228—more than doubling the clustering accuracy relative to the unregularized baseline. These findings indicate that low-rank reconstruction, zero-inflation modeling, variance alignment, and supervised classification each play unique and complementary roles, and that their joint optimization yields the most biologically consistent imputation outcomes. Therefore, we adopted this four-term configuration in all subsequent experiments to ensure optimal performance and interpretability. However, when the raw data have already been normalized, ${ℒ}_{zscore}\;$ often provides limited benefit and may become redundant, and is therefore not recommended. We also compared method performance across different hyperparameter combinations before and after incorporating priors, and assessed stability with versus without priors. The results show that introducing prior knowledge can effectively improve model performance.

However, we observe that under the configurations of ${ℒ}_{NMF}\;$, ${ℒ}_{ZINB}\;$, and ${ℒ}_{class}\;$, scZN appears to outperform scZN_priorNMF. Therefore, we conducted multiple rounds of experiments on ERCC Spike-in, Time-course, and Dentate Gyrus scRNA-seq datasets under the joint regularization of the NMF, ZINB, and classification losses. The results demonstrate that scZN_priorNMF consistently achieves superior and more stable performance. In contrast, the seemingly better results obtained by scZN in some cases can be largely attributed to fluctuations caused by random initialization. When considering both the best-case performance and the average performance across multiple runs, scZN_priorNMF still exhibits overall better performance (see S2 Table) and effectively improve stability in the matrix optimization results.

### Robustness analysis of prior labels

In addition, we systematically examined the robustness of scZN_priorNMF to the quality of prior labels. We conducted additional experiments on a human brain dataset using three types of labels: (i) randomly generated incorrect labels, (ii) clustering-based labels obtained using the Leiden algorithm, and (iii) high-quality manual annotations. Under the guidance of manually annotated labels, scZN_priorNMF achieves the best performance across multiple evaluation metrics. Even when using clustering-based labels, scZN_priorNMF still demonstrates strong imputation performance (Fig 2g).

To systematically assess how model performance depends on the availability and reliability of cell-type labels, we conducted two complementary sensitivity analyses (A1 and A2) on all datasets in D1 (Results for all datasets are presented in S2 Fig).

(A1) Label fraction sensitivity. We varied the fraction of available cell-type labels from 0%, 5%, 10%, 25%, 50%, to 100%, and evaluated downstream classification performance using Accuracy and Macro-F1. Fig 2h shows the results on the Time-course scRNA-seq dataset. Performance improves smoothly as more labels are provided and gradually saturates when sufficient labels become available. Importantly, no abrupt performance jump is observed in the high-label regime, indicating that the method does not degenerate into a purely supervised model. Even with only partial labels, the generative structure learned from expression data remains effective.

(A2) Label perturbation analysis. To further verify that performance gains are not caused by label leakage, we performed a controlled perturbation experiment. Specifically, we progressively injected noise into the label files used to construct $H_{\text{Prior}}\;$ and $W^{+}\;$ at noise levels of 10%, 20%, 30%, and 40%, while still evaluating against the original ground-truth labels. As shown in Fig 2h, both ARI and SI decrease monotonically as noise increases, confirming that the model’s sensitivity to label quality is interpretable and controllable, and that no label information leakage occurred.

### scZN does not introduce spurious biological signals

**Fig 3:**
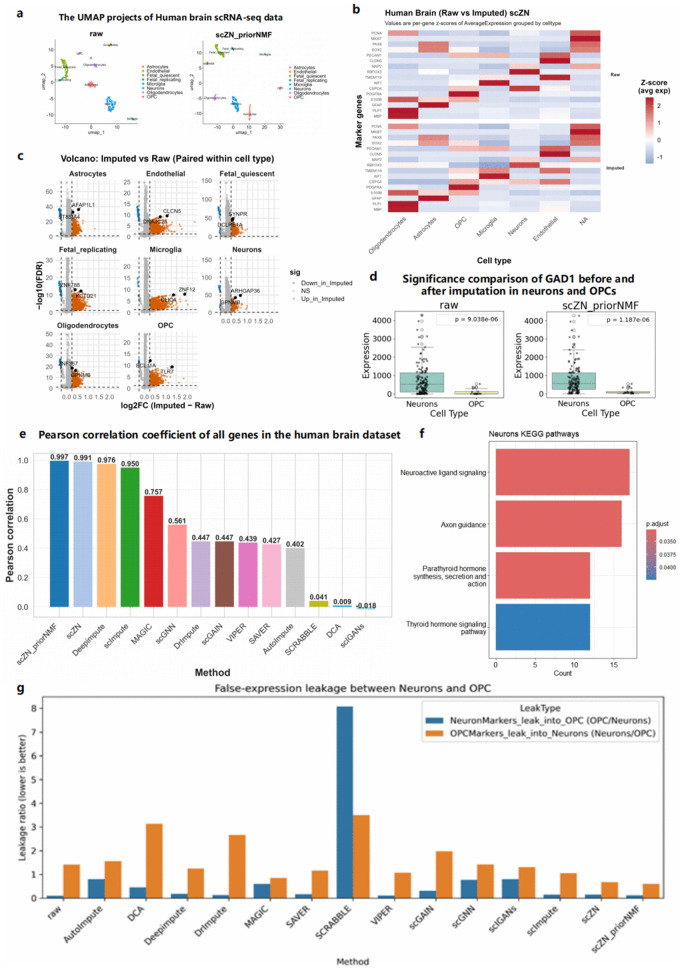
(a) UMAP comparison between the raw data and the scZN-imputed results. (b) Heatmap of two sets of marker genes per cell in the human brain dataset after scZN imputation. (c) Volcano plot comparison of all cell imputation results in the human brain dataset. (d) Significant changes in the imputed data relative to the raw data. (e) Gene–gene correlation before and after imputation, quantified by Pearson correlation coefficients. (f) KEGG pathways enriched among significantly upregulated genes in human brain Neurons following scZN imputation. (g) False-expression leakage between Neurons and OPC.

Most existing imputation methods rarely systematically assess whether they introduce exogenous noise or spurious biological signals, leaving the reliability of imputed data in doubt. To address this, we performed a multi-faceted evaluation on the Human Brain dataset. First, we examined post-imputation cell distributions and global structure using UMAP (Fig 3a). scZN_priorNMF improved both the overall structure and the sharpness of cluster boundaries relative to the raw data and baseline methods.

To further evaluate the impact on biological signal, we generated heatmaps showing two marker genes per cell type for each method (Fig 3b; S3 Fig). We found that AutoImpute, DCA, MAGIC, scGNN, scIGANs, and SCRABBLE substantially perturbed marker-gene expression patterns: genes expected to be highly expressed were suppressed to near silence, and aberrant upregulation appeared in unrelated cell types. scFP performs imputation only on the top 2,000 highly variable genes selected by Scanpy [36]. Because HVG sets differ across tools, key markers were omitted, preventing complete heatmap rendering. Its imputed values effectively resembled noise injection, limiting utility for downstream analyses. By contrast, the remaining methods largely preserved the original biological signal across cell types.

Having identified methods that preserve native expression patterns, we next asked whether gene-level imputation systematically alters expression. Using volcano plots, we observed that most methods elevate a subset of genes—as expected when mitigating dropout—whereas scMASKGAN and SAVER showed virtually no up- or down-regulated genes (Fig 3c; S4 Fig). This behavior likely reflects an overly conservative design that restricts imputation to the local manifold around each cell type. While such conservatism minimizes artifactual changes, it also fails to recover bona fide biological signal.

To assess marker specificity, we compared differential significance after imputation. Using GAD1 as a sentinel marker—expected to be significant in neurons but not in OPCs—only MAGIC, DeepImpute, scZN, and scZN_priorNMF reproduced the anticipated pattern, preserving neuronal significance without spurious elevation in OPCs. By contrast, SCRABBLE and scIGANs disrupted this behavior: they abolished the expected neuronal significance (P > 0.05) and/or produced spurious changes, undermining biological plausibility (Fig 3d; S5 Fig). Moreover, we extended our analysis to multiple canonical Neuron and OPC marker genes, and validated the results on the human brain dataset. Specifically, we selected the Neuron markers SLC17A7, SLC6A1, and GRIN1, as well as the OPC markers CLDN11, CSPG4, and SLC44A1. We compared the expression distributions between neurons and OPCs. For genes that already show clear cell-type separation in the raw data (e.g., SLC17A7 and CLDN11), scZN_priorNMF further strengthens the statistical significance between neurons and OPCs. More importantly, for SLC6A1, GRIN1, CSPG4, and SLC44A1, which fail to show significant cell-type differences in the raw data due to severe dropout, scZN_priorNMF successfully recovers their expected expression patterns in neurons and OPCs and restores statistically significant cell-type differences(S6 Fig).

Finally, we compared Pearson correlation coefficients before and after imputation across methods. scZN and scZN_priorNMF ranked first and second, respectively, achieving the highest Pearson correlation (Fig 3e). These results indicate that both methods denoise while preserving the native correlation structure, whereas several baselines substantially deviated from the original data—consistent with their reduced marker specificity.

Building on this, we performed KEGG enrichment on upregulated genes for each cell type using scZN_priorNMF. In Neurons (Fig 3f), significantly enriched pathways included Neuroactive ligand–receptor interaction and Axon guidance—two canonical neuronal programs—as well as endocrine modules (Thyroid hormone signaling and Parathyroid hormone synthesis, secretion, and action) that modulate neuronal differentiation and excitability. Comparable, cell type–appropriate enrichments were observed across other lineages (S7 Fig), with no inflation of irrelevant pathways. Collectively, these analyses show that scZN delivers top-tier denoising performance without introducing spurious biological signals.

To quantitatively assess whether imputation introduces spurious biological signals, we performed a false-positive expression leakage analysis using 20 groups of differentially expressed marker genes for Neurons and OPCs, respectively. This analysis measures two complementary error modes: (i) leakage of neuronal marker gene expression into OPCs, and (ii) leakage of OPC marker gene expression into neurons. These leakage rates directly quantify the extent to which an imputation method blurs true cell-type-specific expression patterns. The leakage ratio is defined as the ratio of the average expression of marker genes in the incorrect cell type to that in the corresponding correct cell type, with lower values indicating less false-positive expression leakage. As shown in Fig 3g, scZN_priorNMF achieves the lowest leakage rates between Neuron and OPC, indicating that it introduces the fewest false-positive expression signals across cell types, followed by scZN. This suggests that scZN_priorNMF and scZN produce the most reliable imputation results.

### Robust reconstruction of gene–gene correlations

**Fig 4:**
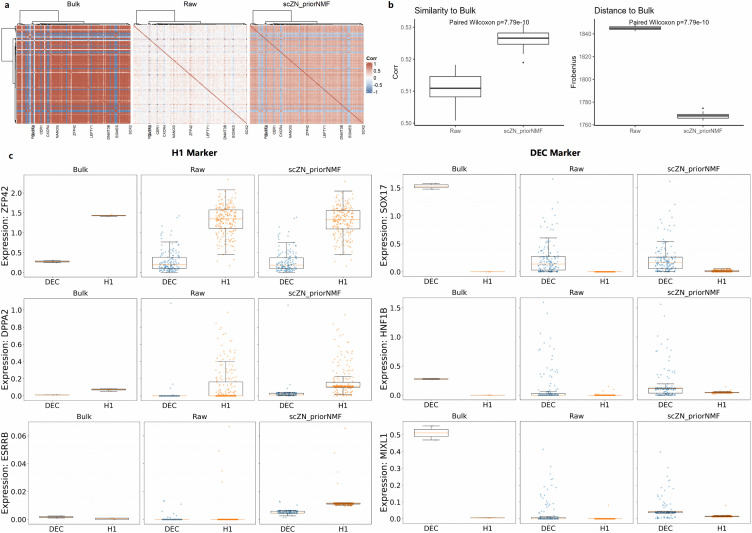
Consistency with bulk RNA-seq and recovery of cell-type–specific gene expression. **(a)** Gene–gene correlation heatmaps computed from bulk RNA-seq data, raw single-cell RNA-seq data, and scZN_priorNMF-imputed data. Hierarchical clustering is applied consistently across panels. **(b)** Quantitative comparison of similarity to bulk RNA-seq. Left: distribution of correlation similarity scores between single-cell data and bulk RNA-seq. Right: Frobenius distance between gene–gene correlation matrices derived from single-cell and bulk RNA-seq data. Statistical significance is assessed using paired Wilcoxon signed-rank tests. **(c)** Expression distributions of representative marker genes for the H1 and DEC cell types, shown for bulk RNA-seq, raw single-cell data, and scZN_priorNMF-imputed data. Box plots and overlaid points illustrate gene expression levels across cell types.

We further evaluated how imputation affects the internal gene–gene correlation structure of the expression matrix. Because bulk RNA-seq can estimate gene co-expression patterns more reliably than sparse single-cell data, it serves as a useful reference for assessing correlation structure. We therefore used a Human ESC scRNA-seq dataset comprising H1 human embryonic stem cells (hESCs) and differentiated endoderm/mesoderm-like cells (DEC), and compared gene–gene correlations derived from raw scRNA-seq data, scZN_priorNMF-imputed scRNA-seq data, and bulk RNA-seq data. This analysis consists of three parts. First, we visualized gene–gene correlation matrices using heatmaps (Fig 4a). The results show that correlation patterns derived from scZN_priorNMF-imputed data exhibit substantially higher concordance with bulk RNA-seq than those derived from raw scRNA-seq data.

Second, to evaluate the robustness of gene–gene correlation structures after imputation, we performed a stability analysis. Specifically, we treated the bulk RNA-seq correlation matrix as a fixed reference and randomly subsampled 20% of cells from both the raw and imputed scRNA-seq expression matrices. Agreement with the bulk reference was quantified using two complementary metrics: (i) the Pearson correlation between the upper-triangular elements of the correlation matrices (similarity; higher is better), and (ii) the Frobenius norm of their difference (distance; lower is better). This procedure was repeated for 50 iterations. The distributions of these metrics were visualized using box plots (Fig 4b). The results indicate that scZN_priorNMF consistently increases similarity to bulk RNA-seq correlations while reducing distance, demonstrating that the recovered correlation structure is both effective and robust.

Third, we visualized the expression patterns of H1 marker genes (ZFP42, DPPA2, and ESRRB) and DEC marker genes (SOX17, HNF1B, and MIXL1) (Fig 4c). The results show that, relative to bulk RNA-seq, scZN_priorNMF-imputed data preserve the gene–gene correlation structure observed in the raw data for genes that already display differential expression, while introducing minimal additional noise. Notably, for certain genes that exhibit differential expression in bulk RNA-seq but appear non-differential in raw scRNA-seq data due to dropout, scZN_priorNMF is able to recover these biologically meaningful differences. In particular, ESRRB is expected to be highly expressed in H1 cells. However, this pattern is not clearly observed in the raw scRNA-seq data. After scZN_priorNMF imputation, the expected cell-type-specific expression of ESRRB is successfully restored. These results indicate that scZN_priorNMF effectively recovers biologically meaningful gene expression patterns and preserves coherent gene–gene correlation networks.

### scZN strengthens cellular trajectory reconstruction

Single-cell RNA-seq (scRNA-seq) not only improves cell-type resolution, but also enables ordering cells along temporal and developmental axes via trajectory inference. Standard trajectory algorithms typically reconstruct a latent path that cells are assumed to traverse and then place cells onto that path. However, these methods generally do not account for zero inflation/dropout, which can distort both manifold geometry and ordering.

**Fig 5:**
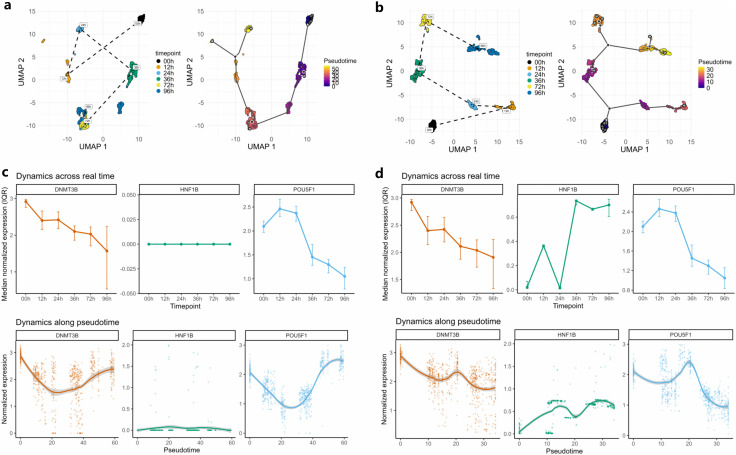
scZN enhances pseudotime analysis with Monocle 3. (a) Pseudotime analysis of the raw embryonic stem cell differentiation data; the left subpanel shows the correct differentiation trajectory derived from temporal labels, and the right subpanel shows Monocle 3’s pseudotime result. (b) Analysis results on the imputed data. (c) and (d): Before/after imputation analyses for DNMT3B, POU5F1, and the DEC marker HNF1B; the top row shows the true temporal progression, and the bottom row shows the pseudotime inference.

To evaluate whether imputation can improve trajectory reconstruction, we analyzed the D2 time-course scRNA-seq dataset of H1 embryonic stem cells differentiating toward definitive endoderm cells (DEC) using Monocle 3 [37]. We first generated a 2-D UMAP embedding from the raw data, annotated cells by induction time, and connected timepoints sequentially to depict the empirical chronology (Fig 5a, left). We then computed pseudotime with Monocle 3 and overlaid the learned principal graph (Fig 5a, right).

The results indicated two key issues. First, consistent with dropout artifacts, cells at 72 h and 96 h were poorly resolved and appeared intermixed in the raw time-course embedding. Second, the pseudotime ordering was largely discordant: aside from progenitor cells being placed plausibly early, most subsequent timepoints were misassigned. Marker dynamics reinforced these concerns. The pluripotency genes DNMT3B and POU5F1 (OCT4), and the DEC marker HNF1B, showed trajectories that were globally inconsistent with the raw data; in particular, HNF1B exhibited near-absent expression in the raw counts even though it is expected to increase during endoderm specification. Taken together, these observations indicate that pseudotime estimation on the raw dataset is unreliable and likely confounded by dropout, motivating a direct comparison with imputed data to assess whether imputation restores biologically coherent trajectories.

Applying the same workflow to the scZN_priorNMF–imputed dataset yielded markedly improved structure. In the UMAP embedding, clusters were cleanly separated and the temporal path exhibited minimal overlap (Fig 5b, left). The Monocle 3 pseudotime reconstruction correctly ordered most time points, with only a localized misassignment between 12 h and 24 h (Fig 5b, right). Marker dynamics were likewise improved. In particular, HNF1B expression was restored in a manner consistent with definitive endoderm specification.

A broader comparison across imputation methods (S8 Fig) supported these findings. Among alternatives, only scIGANs and scImpute effectively mitigated UMAP-level mixing. For temporal ordering, scIGANs ranked second to scZN_priorNMF. All other methods failed to improve trajectory inference. Collectively, these results indicate that appropriate imputation—especially scZN_priorNMF—can materially enhance trajectory analysis in time-course scRNA-seq.

### scZN enhances RNA velocity analysis

RNA velocity is a powerful approach that leverages the ratio of spliced to unspliced mRNA to estimate instantaneous changes in gene expression, thereby inferring cell-state transition trajectories. Compared with pseudotime-based analyses, it is more sensitive to subtle cellular changes. However, existing scRNA-seq velocity methods have not accounted for the impact of dropout events. We hypothesize that appropriate imputation can improve RNA-velocity analysis.

**Fig 6:**
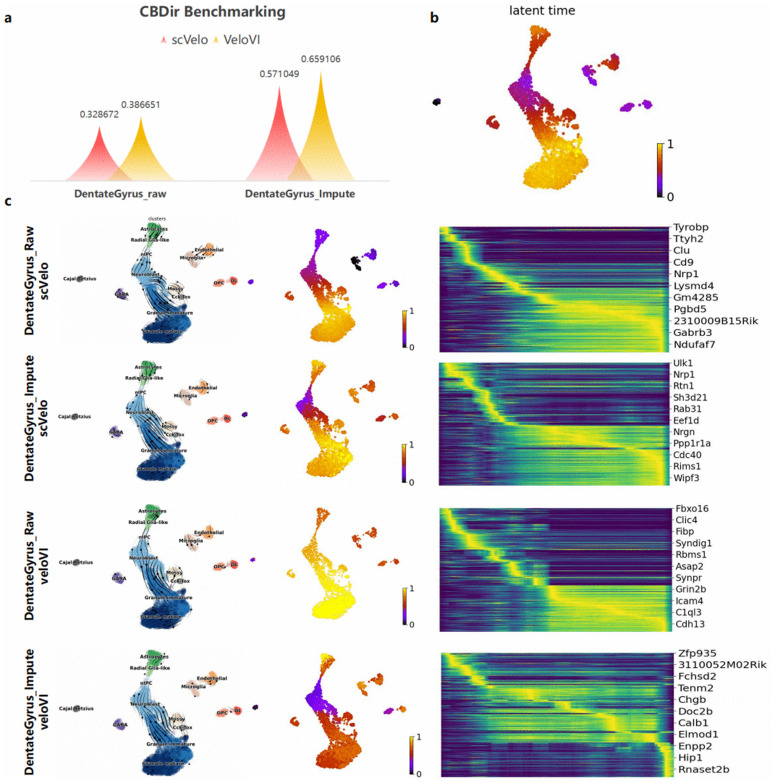
RNA velocity analysis with scVelo and VeloVI on datasets before and after scZN imputation. **(a)** Evaluation of velocity flow accuracy along cell boundaries before and after imputation using the CBDir metric. **(b)** Expression dynamics across latent time in mouse dentate gyrus. **(c)** RNA velocity analyses by scVelo and VeloVI on raw and imputed data, showing (from left to right) the velocity field on the UMAP embedding, velocity-inferred pseudotime, and gene-expression heatmaps.

Using the mouse dentate gyrus dataset with known ground-truth lineages—Radial glia-like → Astrocytes and nIPC → Neuroblast → Granule immature → Granule mature—we ran two RNA-velocity inference models (scVelo and VeloVI) before and after imputation. We first evaluated cross-boundary directional consistency to assess trajectory accuracy. As shown in Fig 6a, after imputation both methods improved by more than 25% . We also present latent time (Fig 6b), UMAP velocity streamlines, velocity-derived pseudotime, and gene heatmaps along the inferred timeline. After imputation, these temporal trends become clearer and more accurate. In addition, fitting the spliced/unspliced dynamics for the genes in the heatmap yielded trajectories and temporal ordering that were more consistent with expectations (S9 Fig). Together, these results demonstrate that scZN mitigates dropout effects and enhances RNA velocity analysis.

### Alzheimer’s disease scRNA-seq analysis

**Fig 7:**
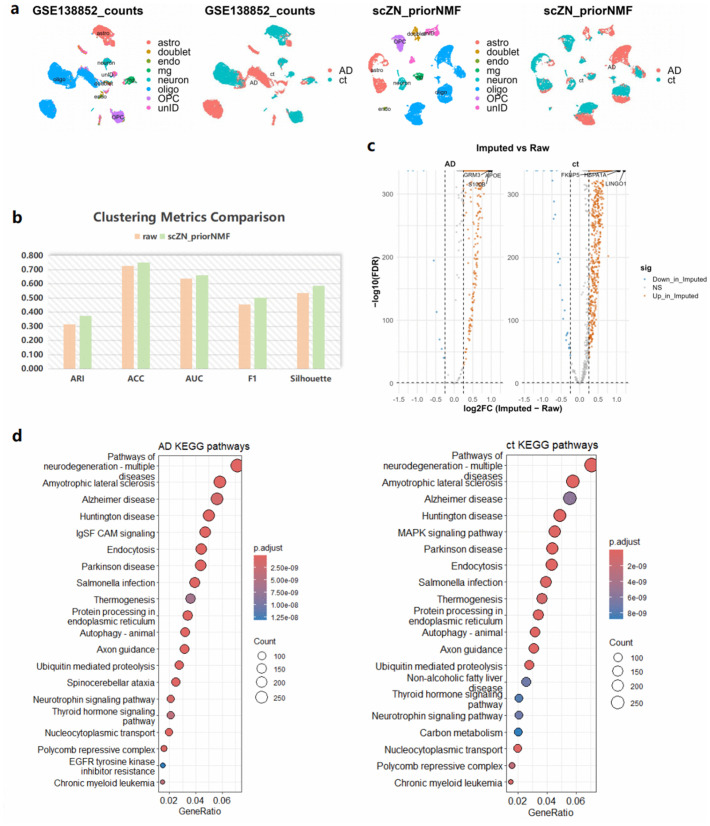
scRNA-seq analysis of Alzheimer’s disease (AD) data with scZN imputation. (a): UMAP cell-type classifications and AD vs. control comparisons before and after imputation. (b): External clustering metrics compared before and after imputation. (c): Genes significantly upregulated in the imputed data relative to the original AD and control datasets. (d): KEGG pathway analysis of the two upregulated gene sets.

To evaluate the applicability of scZN in real biological contexts and on extremely sparse scRNA-seq data, we performed imputation and downstream analyses on the GSE138852 dataset (10,850 genes and 13,124 cells, dropout rate 93.53%, including Alzheimer’s disease (AD) and control (ct)). In the original data, the UMAP representation showed pronounced cluster adhesion and weak separation of cellular heterogeneity (Fig 7a). After scZN imputation, cellular heterogeneity increased and clustering metrics improved (Fig 7b). Pairwise differential analyses with volcano plots (Fig 7c) and KEGG enrichment (Fig 7d) further indicated that among upregulated pathways the AD group was significantly enriched for IgSF CAM signaling, Spinocerebellar ataxia, and EGFR tyrosine kinase inhibitor resistance, which suggests enhanced adhesion and immune activity and stress responses downstream of receptor tyrosine kinases. The ct group showed upregulation of the MAPK signaling pathway, Non-alcoholic fatty liver disease, and Carbon metabolism, indicating more intact canonical signaling and mitochondrial carbon-metabolic homeostasis. This pattern aligns with the canonical molecular landscape of AD, characterized by heightened neuroinflammation, blood–brain barrier and glial responses along with impaired energy metabolism and synaptic homeostasis [38]. Therefore, scZN can effectively enhance cell-type resolution in highly sparse single-cell transcriptomic data while preserving biologically meaningful differential signals.

## Discussion

We present scZN, a single-cell RNA-seq (scRNA-seq) imputation framework that directly addresses two pervasive shortcomings of current deep learning methods: (i) treating all zeros as missing values and applying global smoothing, and (ii) restricting imputation to highly variable genes (HVGs) selected by Scanpy, which forces downstream analyses to depend on a consistent HVG selection tool. The core innovation of scZN is not to propose another smoother, but to redefine imputation as an interpretable factorization process jointly constrained by a count-based observation model and biological priors.

Specifically, scZN performs optimization over the entire gene set within an NMF space: we use per-cell library-size factors for observation-level normalization to decouple technical scale from biological signal and inject cell-type priors via linear shrinkage and anchor gene–module factors to prototype expression means, thereby alleviating the non-convexity of nonnegative matrix factorization (NMF) and the instability of random initialization. We impute only zero values to preserve reliable nonzero counts, and employ composite regularization to further refine the estimates. In comprehensive benchmarking across multiple real datasets, scZN consistently outperforms more than a dozen competing methods while maintaining biological consistency and improving pseudotime and RNA velocity analyses. In sum, the core contribution of scZN is to place imputation within an interpretable NMF space that simultaneously accounts for count statistics and biological priors, thereby addressing common problems of deep learning models at their source. Moreover, during the AD analysis, we observed upregulated neuroinflammation-related pathways, which both align with previous research and validate the effectiveness and real-world applicability of scZN.

Despite scZN’s strong performance across multiple benchmarks, it still has limitations: it requires pre-specifying the factorization rank k, which is currently chosen via clustering, and a poor choice can degrade performance. After introducing deep-learning–based optimization, the computational cost and training time become higher than with purely statistical models. Moreover, under the unsupervised setting, scZN achieves only limited improvement over the raw data and ranks third among the evaluated methods (Fig 2c). This result highlights a fundamental limitation: without external supervision or biological priors, recovering complex gene expression structure from sparse scRNA-seq data remains intrinsically challenging. In the absence of label or module-level constraints, the factorization problem is highly underdetermined, particularly under severe dropout, which limits the achievable performance gains of unsupervised imputation. Therefore, in future work we will improve the framework to enhance its imputation performance in unsupervised settings. In addition, we will extend interpretable factorization—constrained by count statistics and biological priors—to multi-omics and spatial data (e.g., scATAC-seq, multiome, and spatial transcriptomics). For reference mapping and data integration, we will use spatial coordinates or histological images as priors to build spatially constrained, interpretable factorizations that enable cross-modal “spatial remapping” and unified imputation. We also plan to incorporate spliced/unspliced counts into the modeling to improve the joint analysis of pseudotime and RNA velocity.

## Methods

### Ethics statement

No human or animal subjects were involved in this study. All data analyzed were obtained from published literature or public databases, and thus ethical approval was not required.

### Data preparation

We compiled a comprehensive collection of scRNA-seq datasets, which can be categorized into four distinct groups. The first group (D1), sourced from Xu et al. [25], comprises seven datasets representing a broad spectrum of data types and experimental platforms. These datasets include Human brain scRNA-seq data, which provides high-quality data from human brain tissues for assessing imputation in complex biological systems. (dropout 81%) https://www.ncbi.nlm.nih.gov/geo/download/?acc=GSE67835;

ERCC spike-in RNA scRNA-seq dataset, featuring spike-in RNA molecules and an atypical setting with more cells than genes, is used to benchmark imputation performance on extremely small gene expression matrices. (dropout 33%) https://www.ebi.ac.uk/biostudies/arrayexpress/studies/E-MTAB-2512;

A mouse embryonic stem cell (ESC) scRNA-seq dataset, encompassing embryonic stem cell differentiation and large-scale measurements, is used to evaluate imputation performance in developmental contexts. (dropout 70%) https://www.ncbi.nlm.nih.gov/geo/download/?acc=GSE65525;

Time-course scRNA-seq data are used to evaluate the ability of imputation methods to preserve temporal gene expression dynamics under a high dropout rate. (dropout 55%) https://www.ncbi.nlm.nih.gov/geo/download/?acc=GSE75748;

sc_Drop-seq data generated by droplet-based sequencing are used to evaluate cross-platform robustness of imputation methods. (dropout 62%) https://www.ncbi.nlm.nih.gov/geo/download/?acc=GSE118706;

sc_CEL-seq2 data generated by plate-based sequencing are employed to benchmark the adaptability of imputation methods to high-precision platforms. (dropout 74%) https://www.ncbi.nlm.nih.gov/geo/download/?acc=GSE117617;

sc_10X data generated by the 10X Genomics platform are used to evaluate the broad applicability of the method. (dropout 45%) https://www.ncbi.nlm.nih.gov/geo/download/?acc=GSE111108.

The second group (D2), derived from Aivazidis et al. [39], comprises dentate gyrus scRNA-seq data, a challenging setting where most RNA velocity inference methods perform poorly. This dataset is therefore used to evaluate whether imputation can enhance inference accuracy.

The third group (D3) consists of an Alzheimer’s disease (AD) scRNA-seq dataset that provides gene expression profiles for both AD and control samples and exhibits an extremely high dropout rate (93.53%). https://www.ncbi.nlm.nih.gov/geo/query/acc.cgi?acc=GSE138852. We therefore used it to assess the practical utility of scZN.

The fourth group (D4) consists of a human embryonic stem cell (ESC) scRNA-seq dataset for differential expression analysis. This dataset includes six bulk RNA-seq samples, which are used as a reference to compare gene–gene relationships before and after imputation. https://www.ncbi.nlm.nih.gov/geo/query/acc.cgi?acc=GSE75748

In addition, prior to all result analyses, we performed Seurat-based quality control on the scRNA-seq data [40], specifically excluding mitochondrial (MT) genes and selecting 2,000 highly variable genes for analysis.

### The framework of scZN

#### scZN generative framework.

In scZN, we assume that whether a zero entry for a gene in a given cell should be imputed is not determined by the zero value itself, but rather by the position of this entry in a latent similarity space. Specifically, if a gene exhibits consistent and stable nonzero expression among cells that are similar to the target cell, then an observed zero is more likely to arise from technical dropout; conversely, if the gene is intrinsically lowly expressed or highly variable among similar cells, the zero more likely reflects true biological absence and should not be corrected. This principle motivates a generative formulation in which imputation is driven by a latent biological expression generator defined over a similarity space, rather than by heuristic rules applied directly to observed zeros.

Let the scRNA-seq count matrix be denoted by $𝐗\in {\mathbb{N}}_{0}^{n\times m}\;$, where *n* is the number of cells and *m* is the number of genes. We first estimate a cell-specific size factor that captures library-size and capture-rate variation exclusively at the observation layer:


$$s_{i}\;=\;\frac{\sum_{j=1}^{m}X_{ij}}{{\mathrm{median}}_{\;r}\;\sum_{j=1}^{m}X_{rj}},\;i=1,\ldots ,n.\;$$ (1)

This choice is justified by Poisson thinning: if the biological molecule counts satisfy $Y_{ij}^{bio}\sim Poisson(\lambda_{ij}$ and each molecule is captured independently with a cell-specific probability $p_{i}\;$, then the observed counts follow $Y_{ij}^{obs}\sim Poisson(p_{i}\lambda_{ij}$, implying $s_{i}\propto p_{i}\;$. Thus, technical scaling is handled by $s_{i}\;$ at the observation layer, without entangling sequencing depth with the biological mean.

Our purpose is to generate a matrix μ  that represents the imputed results inferred by the model. Using PyTorch [41], we first initialize two trainable factor matrices: $𝐖\in {\mathbb{R}}^{n\times k}\;$ and $𝐇\in {\mathbb{R}}^{k\times m}\;$, where *k* denotes the number of cell types, cell clusters, or latent biological modules. The generated matrix ***μ*** is defined as the matrix product of these two factors:


μ=𝐖𝐇  (2)

The *i*-th row ${𝐰}_{i}\;$ of matrix **W** represents the nonnegative module membership probabilities of cell *i*, which can be interpreted as a probability distribution over *k* modules; the *l*-th row ${𝐡}_{l}\;$ of matrix 𝐇  describes the gene expression profile associated with module *l*. The key interpretation of this generative matrix is as fo*l*lows: for a given cell–gene pair (i,j, if cell *i* is close, in the induced simi*l*arity space, to a group of cells that stably and highly express gene *j* (i.e., ${𝐰}_{i}\;$ is close to the representations of those cells), then a relatively reasonable value $\mu_{i,j}\;$ will be produced via the inner product as the imputed result. This supports the interpretation that the observed zero value is more likely due to technical dropout.

Conversely, if the gene exhibits low expression or high variability among similar cells, the generator will naturally produce a very small value of $\mu_{i,j}\;$, thereby avoiding over-correction of zero entries.

When cell type labels or other module annotations are available, scZN incorporates this prior information to link latent cell abundance factors with known biological characteristics, resulting in scZN_priorNMF.

Let $𝐏\in \{0,1{\}}^{n\times k}\;$ denote a one-hot (or soft) encoding matrix of cell type or module assignments. To ensure nonnegativity, we define $\hat{𝐖}=ReLU(𝐖$ and $\hat{𝐇}=ReLU(𝐇$, and obtain a prior-aligned cell factor via an equally weighted row-wise softmax operation:


$${𝐖}^{+}\;=\;\frac{1}{2}\;(𝐏+softmax(\hat{𝐖}))\;$$ (3)

To guide ***μ*** toward canonical expression patterns and reduce sensitivity to random initialization, we generate a simple prototype expression matrix ${𝐇}_{prior}\in {\mathbb{R}}^{k\times m}\;$ based on cell-type-wise averages:


$${𝐇}_{prior}(i,j)\;=\;\frac{1}{\left|{𝒞}_{i}\right|}\sum_{c\in {𝒞}_{i}}X_{c,j},\;i=1,\ldots ,k,\;j=1,\ldots ,m,\;$$ (4)

where ${𝒞}_{i}\;$ denotes the index set of cells assigned to the *i*-th cell type, and $|{𝒞}_{i}|\;$ is the number of cells in the index set.


$${𝐇}^{+}\;=\;\frac{1}{2}\;({𝐇}_{prior}+\hat{𝐇}),\;\hat{𝐇}=ReLU(𝐇).\;$$ (5)

Finally, we obtain the imputed expression matrix through the prior-aligned factors:


$$\mu \;=\;{𝐖}^{+}{𝐇}^{+}\;$$ (6)

which represents the inferred biological-scale mean expression for each cell–gene pair. To compare the inferred means with the observed scRNA-seq counts, we project ***μ*** back to the observation layer by accounting for cell-specific sequencing depth. Let $𝐬\in {\mathbb{R}}_{>0}^{n}\;$ denote the size-factor vector; the reconstructed count matrix is given by


$${\hat{𝐗}}^{\left(recon\right)}\;=\;diag(𝐬)\;\mu \;+\;\varepsilon \;$$ (7)

where *ε* denotes stochastic observation noise.

When gene-specific batch effects are present, we further incorporate a multiplicative correction term. Let $\kappa_{j,b(i)}\;$ denote the batch effect for gene *j* in the batch b(i of cell *i*. The reconstruction then becomes


$${\hat{X}}_{ij}^{\left(recon\right)}\;=\;s_{i}\;\kappa_{j,b(i)}\;\mu_{ij}\;+\;\varepsilon \;$$ (8)

where the identifiability constraint ensures that batch effects are centered on the log scale and do not confound the biological mean expression.

### Multiple regularization design

Based on the framework described above, it is evident that the learning process in this work is essentially a non-convex optimization problem. Without sufficient structural constraints, the optimization is prone to producing numerically valid yet biologically implausible imputation results. Accordingly, we construct regularization terms from four aspects: the solution space of optimal solutions, data distribution properties, geometric relationships, and interpretability. Specifically, the overall loss is decomposed into the following components: (i) the NMF reconstruction loss (${ℒ}_{\text{NMF}}\;$), which measures the mean Frobenius norm error between the original and reconstructed matrices; (ii) the ZINB [34] negative log-likelihood loss (${ℒ}_{\text{ZINB}}\;$), which models the zero inflation and over-dispersion in the counts; (iii) the z-score [35] regularization loss (${ℒ}_{\text{zscore}}\;$), which ensures consistency in the standardized gene expression profiles within each cell; and (iv) the cell-type classification loss (${ℒ}_{\text{class}}\;$), which leverages cell-type labels to constrain the low-dimensional representation and enhance biological interpretability. The following subsections detail the formulation of each loss component.

#### NMF reconstruction loss.

${ℒ}_{\text{NMF}}\;$ employs the Frobenius norm, which imposes a symmetric quadratic penalty on the reconstruction error of the imputed data under an observation-aligned scale. This is equivalent to assuming a tighter Gaussian tolerance region, which stabilizes the optimization of the non-convex matrix factorization problem. Moreover, the Frobenius norm is the simplest and most stable choice widely adopted in the NMF literature to enforce alignment between the imputed values and the original count matrix. Concretely, let ***μ*** be the biological-scale mean and let $𝐬\in {\mathbb{R}}_{>0}^{n}\;$ denote per-cell library-size factors, we use the depth-calibrated reconstruction $\mu_{\text{adjusted}}=diag(𝐬,\mu \;$. The loss is the mean Frobenius norm [42]:


$${ℒ}_{\text{NMF}}\;=\;\frac{\backslash |𝐗-\mu_{\text{adjusted}}{\backslash |}_{F}}{n\times m},\;$$ (9)

#### ZINB model.

scRNA-seq count data typically exhibit zero inflation (arising from capture dropout and transcriptionally silent states) and overdispersion (due to bursty transcription and cell-to-cell heterogeneity). To constrain these distributional characteristics during reconstruction—without asserting the ZINB model as the true data-generating process—we adopt a ZINB-based distributional regularizer.

Let $x_{ij}\;$ denote the observed count for gene *j* in cell *i*. The ZINB probability mass function $P(x_{ij}$ is defined as


$$\begin{array}{c} \left\{ \begin{array}{c} @l\pi_{ij}+(1-\pi_{ij}){\left(\frac{\theta }{\mu_{ij}+\theta }\right)}^{\theta }\;\;\;\;\;\;\;\;\;\;\;\text{ if }x_{ij}=0,\\ (1-\pi_{ij})\;\frac{\Gamma (x_{ij}+\theta )}{\Gamma (\theta )\;\Gamma (x_{ij}+1)}{\left(\frac{\mu_{ij}}{\mu_{ij}+\theta }\right)}^{x_{ij}}{\left(\frac{\theta }{\mu_{ij}+\theta }\right)}^{\theta }\;\text{if }x_{ij}>0, \end{array}\right.\; \end{array}$$ (10)

where $\mu_{ij}\;$ is the reconstructed mean at entry (i,j, $\pi_{ij}=\sigma (\mathrm{\backslash cdotin}[0,1]\;$ denotes the dropout (structural-zero) probability, and θ>0  is a shared negative binomial dispersion parameter, jointly optimized with $\mu_{ij}\;$ and $\pi_{ij}\;$.

The corresponding regularization term is given by the negative log-likelihood:


$${ℒ}_{\text{ZINB}}\;=\;-\frac{1}{nm}\sum_{i=1}^{n}\sum_{j=1}^{m}\log P(x_{ij}).\;$$ (11)

This regularizer explicitly aligns the zero fraction and the mean–variance relationship of the reconstructed data with empirically observed scRNA-seq behavior, discouraging overly smoothed imputations that erase zero entries or underestimate expression dispersion.

#### Z-score regularization.

Many downstream analyses in single-cell studies—such as marker gene ranking, module activity scoring, and gene co-expression analysis—depend primarily on relative expression patterns within cells rather than absolute count magnitudes. Consequently, preserving the internal geometry of gene expression profiles is often more critical than matching raw counts exactly. By computing the loss on z-score–normalized expression values, we enforce consistency between reconstructed and observed data in a scale-free space that emphasizes relative deviations. This prevents the imputation process from collapsing heterogeneous cellular expression profiles into a common structure and helps preserve intrinsic expression geometry across genes within each cell.

From a maximum a posteriori (MAP) viewpoint, minimizing the mean squared error on z-scores is equivalent to imposing a Gaussian prior on the standardized, scale-invariant relative expression geometry. Under this assumption, the MSE loss naturally arises as the negative log-likelihood associated with that prior. Therefore, for cell *i*,


$${\bar{x}}_{i}=\frac{1}{m}\sum_{j=1}^{m}x_{ij},\;\sigma_{i}=\sqrt{\frac{1}{m}\sum_{j=1}^{m}(x_{ij}-{\bar{x}}_{i}{)}^{2}},\;z_{ij}=\frac{x_{ij}-{\bar{x}}_{i}}{\sigma_{i}+\epsilon },\;$$ (12)

and for the reconstruction,


$${\bar{\mu }}_{i}=\frac{1}{m}\sum_{j=1}^{m}\mu_{ij},\;{\hat{\sigma }}_{i}=\sqrt{\frac{1}{m}\sum_{j=1}^{m}(\mu_{ij}-{\bar{\mu }}_{i}{)}^{2}},\;{\hat{z}}_{ij}=\frac{\mu_{ij}-{\bar{\mu }}_{i}}{{\hat{\sigma }}_{i}+\epsilon }.\;$$ (13)

We penalize standardized discrepancies:


$${ℒ}_{\text{zscore}}\;=\;\frac{1}{nm}\sum_{i=1}^{n}\sum_{j=1}^{m}(z_{ij}-{\hat{z}}_{ij}{)}^{2},\;$$ (14)

so that, after removing per-cell scale/offset, the reconstruction preserves each cell’s intrinsic relative expression shape.

#### Cell-type classification loss.

The purpose of ${ℒ}_{class}\;$ is to anchor ***μ*** to a latent semantic representation. In highly non-convex optimization settings, reconstruction and distributional constraints alone may yield solutions that are numerically valid but biologically uninterpretable. By requiring known cell identities to be linearly decodable from the latent embedding ${𝐖}_{i}^{+}\;$, the classification loss introduces an effective semantic anchor that eliminates such degenerate solutions, without enforcing a one-to-one correspondence between modules and cell types. Let ${𝐖}_{i}^{+}\;$ be the prior-aligned embedding of cell *i*, a linear probe produces class probabilities


$${\hat{𝐲}}^{\;i}=\mathrm{softmax}\;({𝐖}_{i}^{+}\;{𝐰}_{c}+b),\;$$ (15)

with ${𝐰}_{c}\in {\mathbb{R}}^{k\times C}\;$, $b\in {\mathbb{R}}^{C}\;$, and *C* cell types. The cross-entropy is


$${ℒ}_{class}=-\frac{1}{n}\sum_{i=1}^{n}\sum_{c=1}^{C}1\{y_{i}=c\}\;\log{\hat{y}}_{ic}.\;$$ (16)

Because a truly biological module coordinate should be linearly decodable, this term both tests and enforces that ${𝐖}^{+}\;$ carries cell-identity information, suppressing biologically implausible module mixing and stabilizing rare states.

#### Optimization.

We minimize the total objective


$$ℒ\;=\;\lambda_{\text{NMF}}\;{ℒ}_{\text{NMF}}\;+\;\lambda_{\text{zinb}}\;{ℒ}_{\text{ZINB}}\;+\;\lambda_{\text{zscore}}\;{ℒ}_{\text{zscore}}\;+\;\lambda_{\text{class}}\;{ℒ}_{\text{class}},\;$$ (17)

where each coefficient *λ* controls the contribution of its term. All trainable parameters $\Theta =\{𝐖,𝐇,\pi ,\theta ,{𝐰}_{c},b\}\;$ are updated with Adam [43]. Let $g_{t}=\nabla_{\Theta }ℒ(\Theta )\;$. Adam updates are


$$\Theta^{\left(t+1\right)}=\Theta^{\left(t\right)}-\eta_{t}\;\frac{{\hat{m}}_{t}}{\sqrt{{\hat{v}}_{t}}+\epsilon_{adam}}\;$$ (18)

where ${\hat{m}}_{t}\;$ and ${\hat{v}}_{t}\;$ are the bias‐corrected first and second moment estimates, $\epsilon_{adam}=10^{-8}\;$ prevents numerical instability, and $\eta_{t}\;$ is the learning rate, which may be decayed according to


$$\eta_{t}=\eta_{0}\times \gamma^{\lfloor t/T_{decay}\rfloor }\;$$ (19)

with initial rate $\eta_{0}\;$, decay factor γ<1 , and decay interval $T_{decay}\;$.

#### Stopping criterion.

Training stops when the objective plateaus, e.g., the relative change of the (training or validation) loss stays below a tolerance $\epsilon_{\text{loss}}=0.001\;$ for *W* consecutive epochs:


$$\frac{\left|{ℒ}^{\left(t\right)}-{ℒ}^{\left(t-1\right)}\right|}{{ℒ}^{\left(t-1\right)}}<\epsilon_{\text{loss}}\;\text{for }t\in \{t_{0}\;-\;W+1,\ldots ,t_{0}\}.\;$$ (20)

### Parameters of scZN

The main parameters of scZN are the factorization rank *k* and the regularization coefficients. We typically set *k* to the number of cell types to ensure biological interpretability, yielding a factorization into (i) a cell-by–cell-type probability matrix and (ii) a cell-type–by–gene expression matrix.

### Imputation evaluation

We evaluate the accuracy of imputation using ARI, ACC, F1, AUC, and the silhouette coefficient. The computations are as follows:

#### ARI (Adjusted Rand Index).

Pairwise agreement between the predicted partition and ground truth, corrected for chance:


$$ARI=\frac{\text{observed pairwise agreement}-\text{expected agreement at random}}{\text{maximum possible agreement}-\text{expected agreement at random}}\;$$ (21)

#### ACC (Clustering Accuracy).

Match predicted clusters to ground-truth classes via the Hungarian algorithm, then compute the proportion of correctly assigned samples:


$$ACC=\frac{\text{ correct assignments after matching}}{\text{total samples}}\;$$ (22)

#### F1.

Compute precision and recall per class, then aggregate:


$$Precision=\frac{TP}{TP+FP},\;Recall=\frac{TP}{TP+FN},\;F1=\frac{2\;Precision\cdot Recall}{Precision+Recall}.\;$$ (23)

#### AUC (ROC–AUC).

Using one-vs-rest continuous scores, trace the ROC curve across thresholds and take its area. For multiclass, report the macro-average:


$$AUC=\int_{0}^{1}TPR(FPR)\;d(FPR),\;{AUC}_{macro}=\text{mean of classwise AUCs }$$ (24)

#### Silhouette coefficient.

For each sample, compare the mean dissimilarity to its own cluster with that to the nearest other cluster:


$$s(i)=\frac{b(i)-a(i)}{\max\{a(i),\;b(i)\}},\;$$ (25)

where a(i is the average distance from *i* to samples in its own cluster, and b(i is the minimum average distance to any other cluster. The dataset silhouette is the mean of s(i over all samples.

#### CBDir.

We computed the CBDir score using the functions provided by the UniTVelo [44] Python package. In the UniTVelo paper, CBDir evaluates the correctness of the transition from a source cluster to a target cluster by using boundary cells defined by the ground truth. Here, the boundary of the source cluster consists of cells in that cluster that are adjacent to the target cluster, and vice versa. Boundary cells are used because they reflect short-term developmental dynamics. CBDir is defined as


$$\mathrm{CBDir}(c)=\frac{1}{Norm}\sum_{c^{\prime }\in C_{A}\cap N(c)}\frac{{𝐯}_{c}\cdot ({𝐱}_{c^{\prime }}-{𝐱}_{c})}{\left\|{𝐯}_{c}\|\;\|{𝐱}_{c^{\prime }}-{𝐱}_{c}\right\|},\;Norm=|\;\{c^{\prime }\in C_{A}\cap N(c)\}\;|\;$$ (26)

Here, $C_{A}\;$ denotes the set of cells in the target cluster *A*, N(c denotes the neighbors of cell *c*, and ${𝐯}_{c}\;$ and ${𝐱}_{c}\;$ are the inferred velocity and low-dimensional position vectors of cell *c*, respectively. Thus, ${𝐱}_{c^{\prime }}-{𝐱}_{c}\;$ represents the short-term displacement in the embedding space.

#### Gene-level Pearson correlation.

We estimate gene-level consistency before and after imputation using the Pearson correlation as follows. For each gene *g*, compute the Pearson correlation between its expression vector before imputation and after imputation:


$$r_{g}\;=\;\mathrm{corr}(\text{raw}(g),\;\text{imputed}(g))\;$$ (27)

Summarize the dataset-level consistency by aggregating across genes.

## Supporting information

S1 Fig The performance of all methods across D1. Box plots summarizing the performance of all methods across D1 (ERCC spike-in, Human Brain, Timecourse, mESC, scDrop-seq, scCelseq2, and sc10X). Columns correspond to evaluation metrics, and rows represent datasets.
(PDF)

S2 Fig Label sensitivity analysis. Label sensitivity analysis during the imputation process across D1, shaded regions around the curves indicate variability.
(PDF)

S3 Fig Human brain marker gene heatmap. This figure shows the heatmap expression of labeled genes after imputation on a human dataset using multiple methods.
(PDF)

S4 Fig Volcano plots. Genes significantly upregulated by multiple imputation methods.
(PDF)

S5 Fig Significance comparison of GAD1 in Neurons and OPC. Comparison of the significance of GAD1 in Neurons and OPC datasets after imputation using 14 methods.
(PDF)

S6 Fig Significance comparison of marker genes in Neurons and OPC. Significance comparison before and after imputation of SLC17A7, CLDN11, SLC6A1, GRIN1, CSPG4, and SLC44A1.
(PDF)

S7 Fig KEGG pathway. Changes in KEGG pathways of upregulated genes in human brain datasets after scZN imputation.
(PDF)

S8 Fig Monocle 3–based pseudotime analysis. Focusing on comparing pseudotime results using data imputed by other methods.
(PDF)

S9 Fig Gene analysis based on RNA velocity. Focusing on fitting the spliced/unspliced dynamics of the genes in the Fig 5c heatmap and supplementing with corresponding UMAP plots of velocity and gene expression.
(PDF)

S1 Table External consistency evaluation. For the D1 dataset, we evaluated ARI, ACC, AUC, F1, and the silhouette coefficient on the data before and after processing by 14 imputation methods.
(XLS)

S2 Table Robustness Analysis of scZN and scZN_priorNMF across D1. We conducted five independent runs on the ERCC Spike-in, Time-course, and Dentate Gyrus scRNA-seq datasets to assess stability with and without incorporating priors, in order to determine whether the framework’s non-convexity is mitigated.
(XLS)
