# Supplementary figures and images for: Prior-guided factorization for reliable imputation of scRNA-seq data
Source: PLoS Comput Biol. 2026 Mar 20;22(3):e1014051. doi: 10.1371/journal.pcbi.1014051 (PMC13004523; doi:10.1371/journal.pcbi.1014051)

# Evaluation Metrics for All Methods on the Entire D1 Dataset

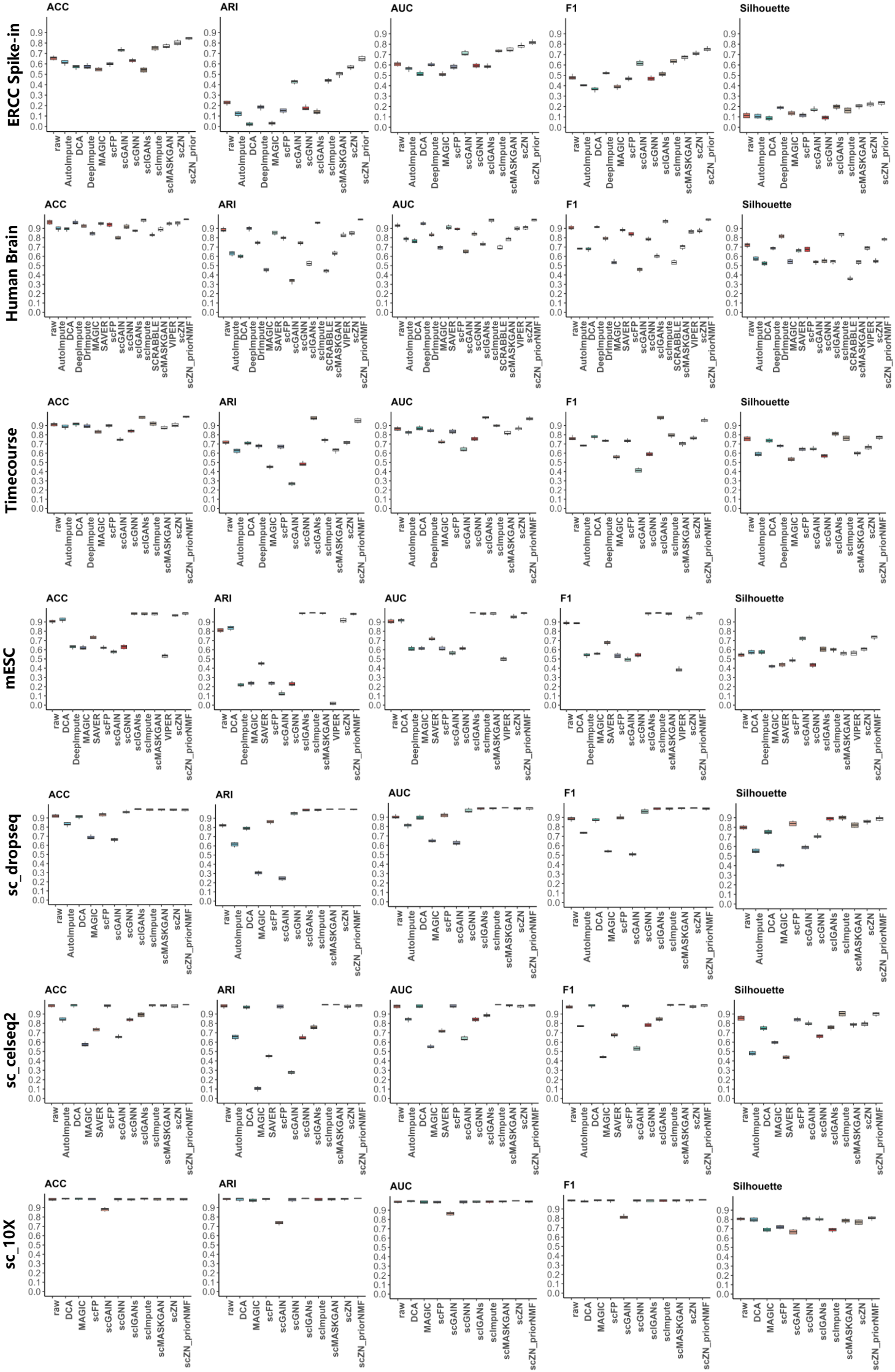

Supplement: S1 Fig — Box plots summarizing the performance of all methods across D1 (ERCC spike-in, Human Brain, Timecourse, mESC, scDrop-seq, scCelseq2, and sc10X). Columns correspond to evaluation metrics, and rows represent datasets. (PDF) [file pcbi.1014051.s001.pdf]

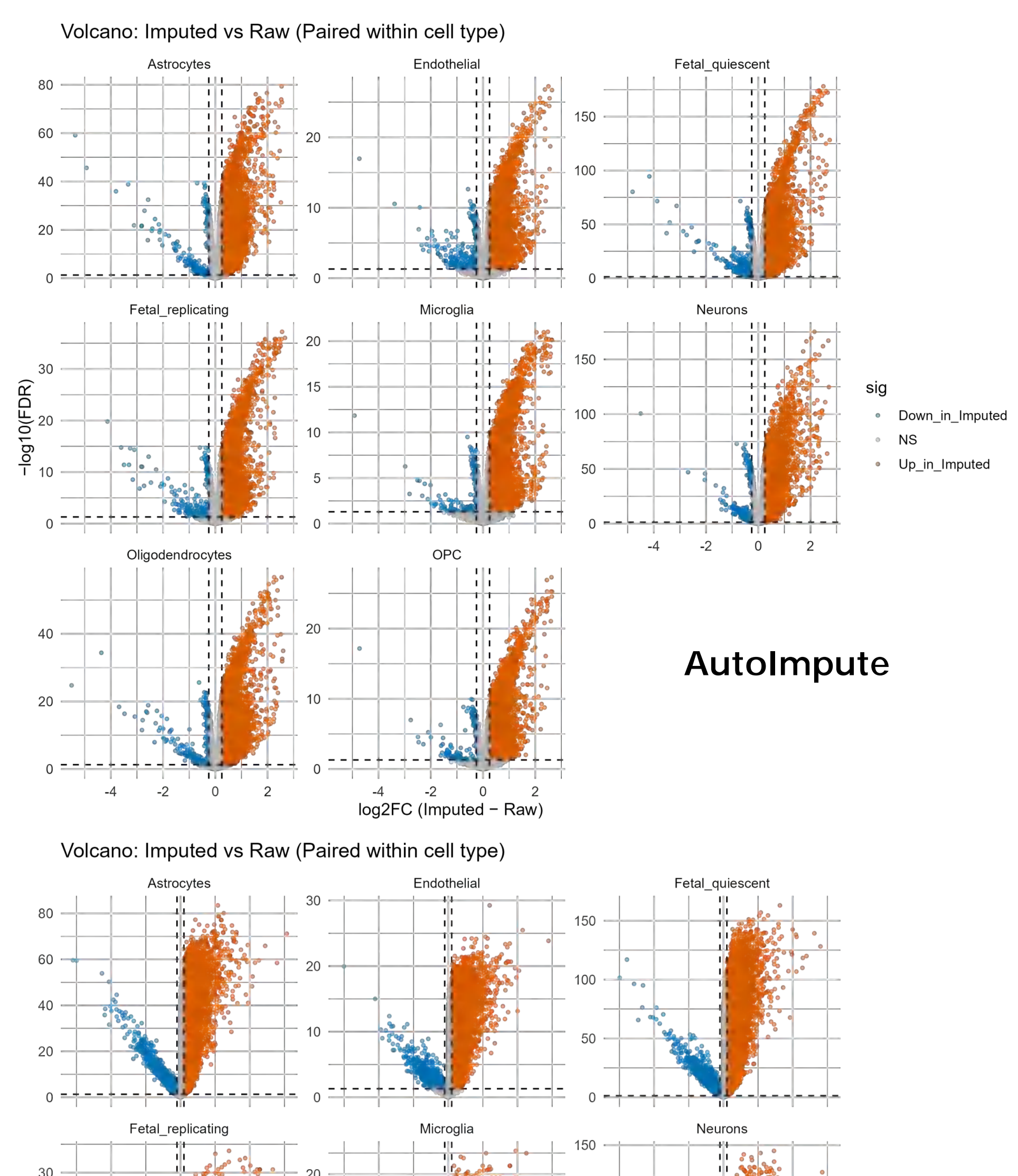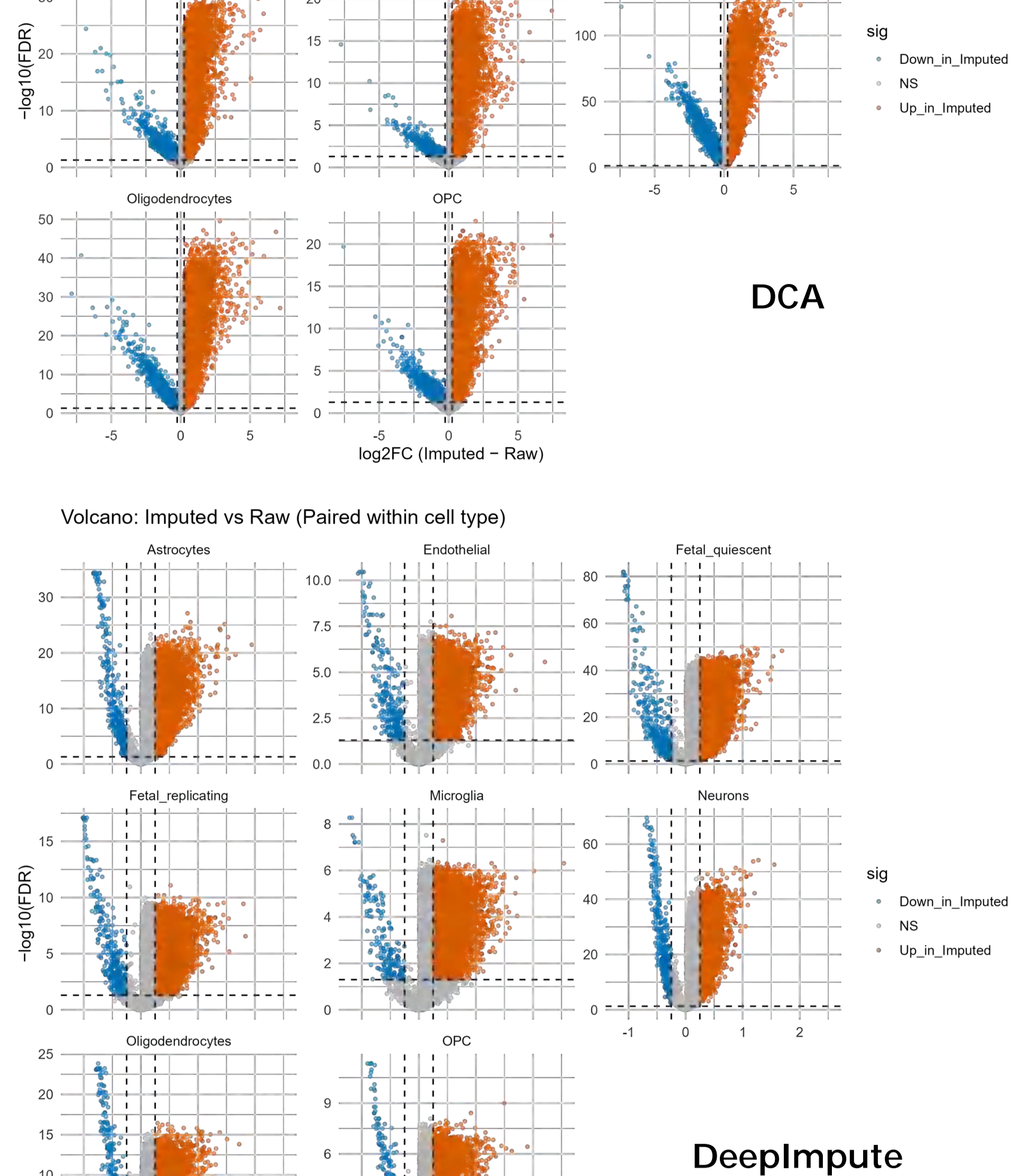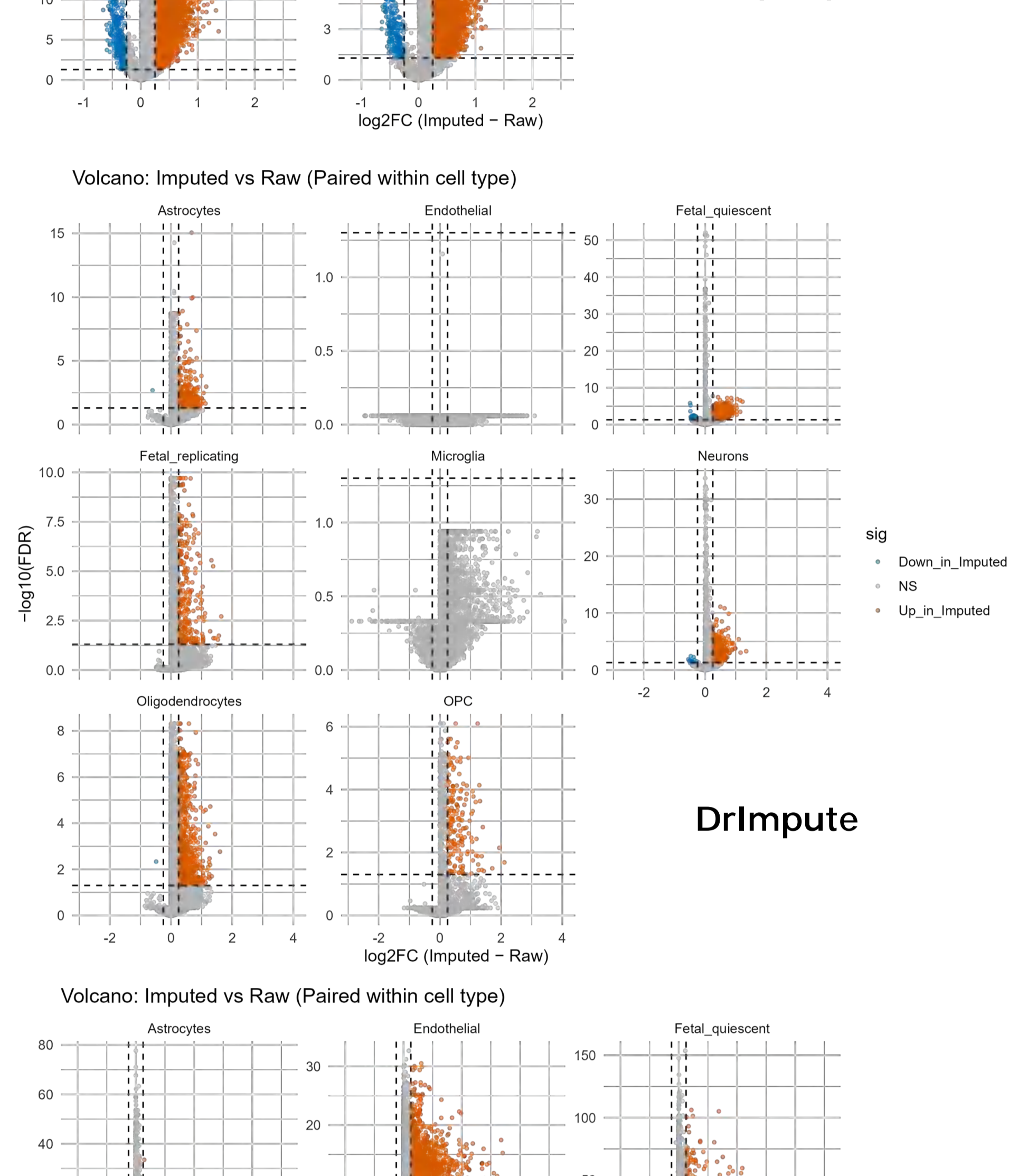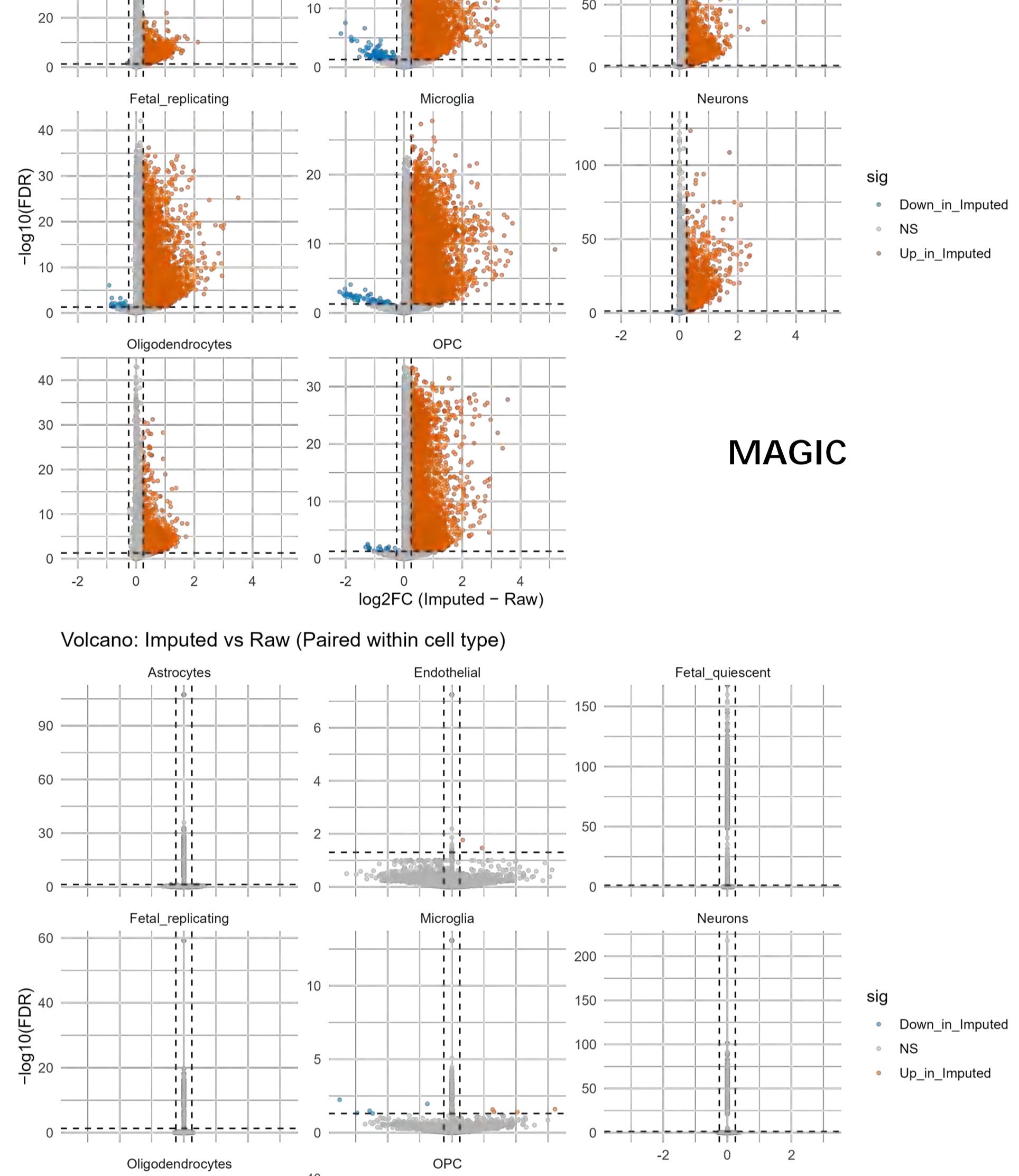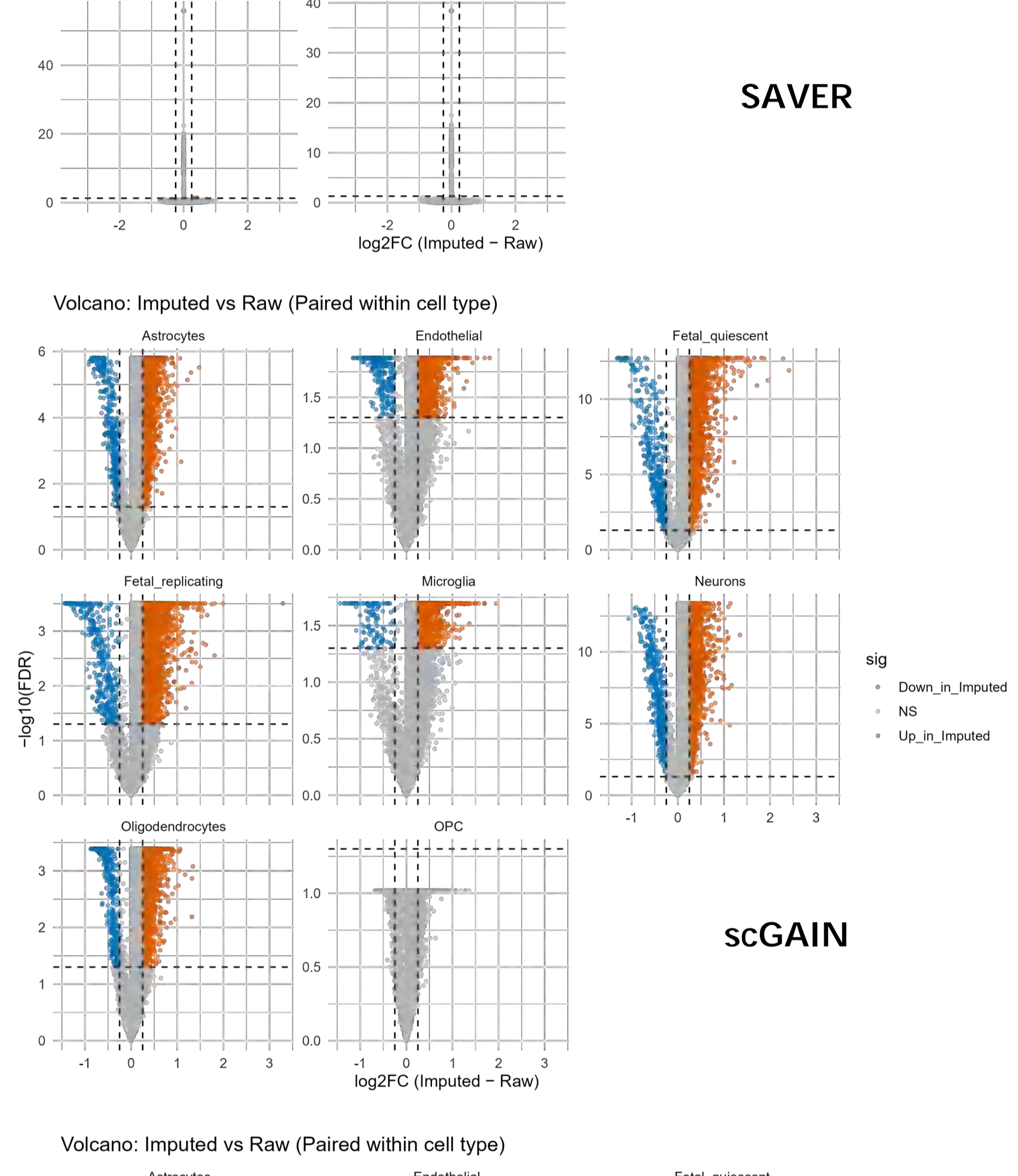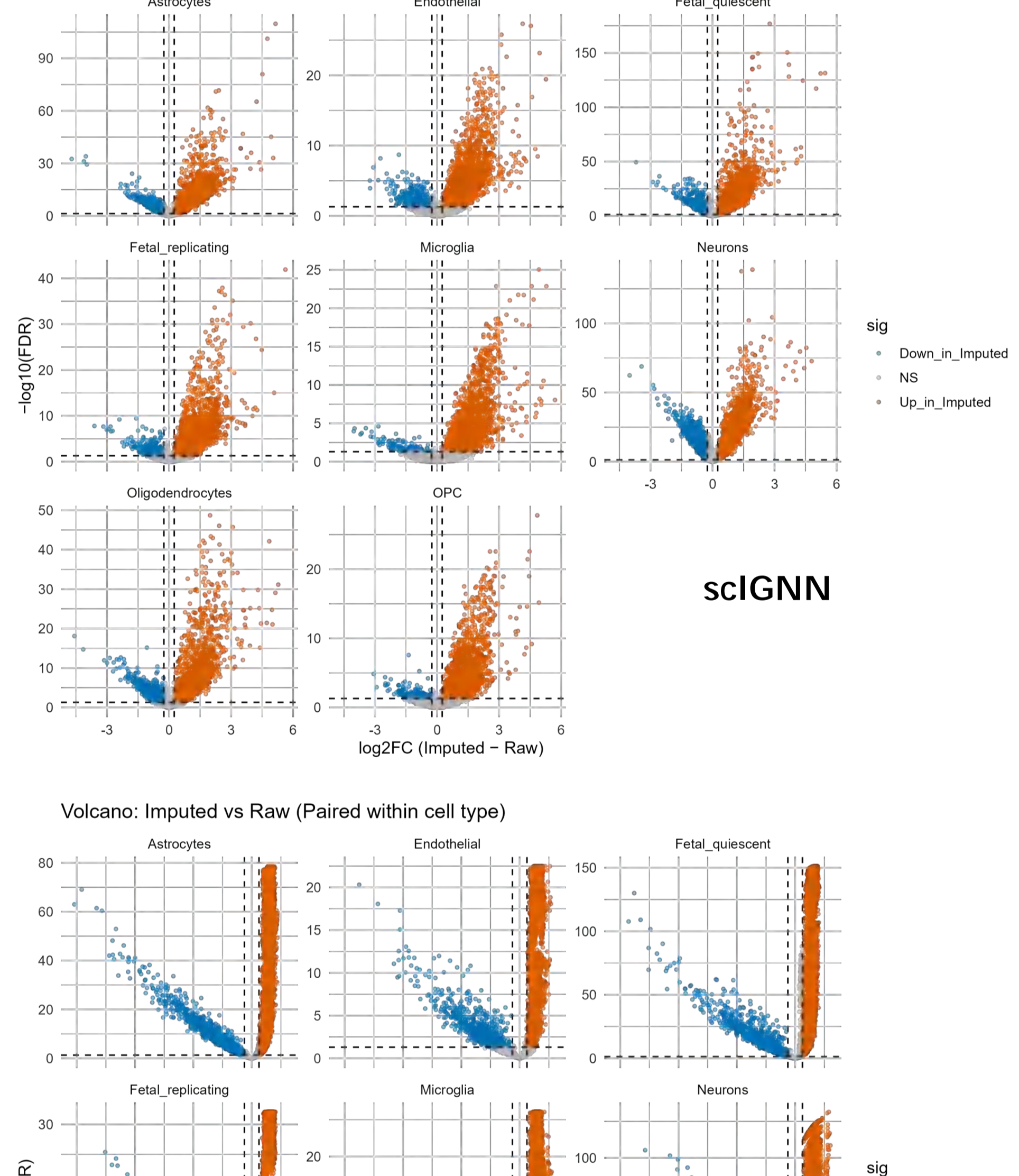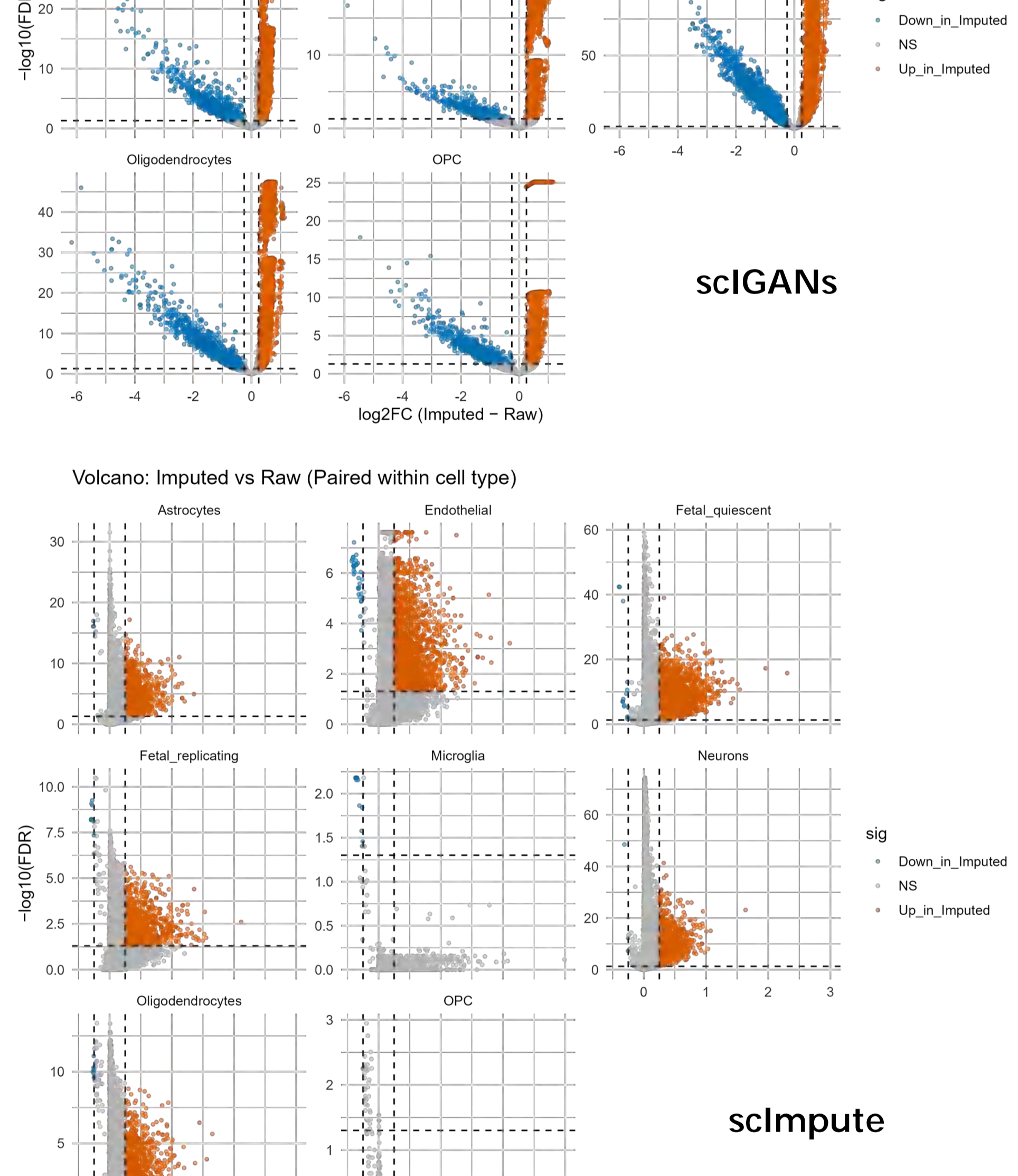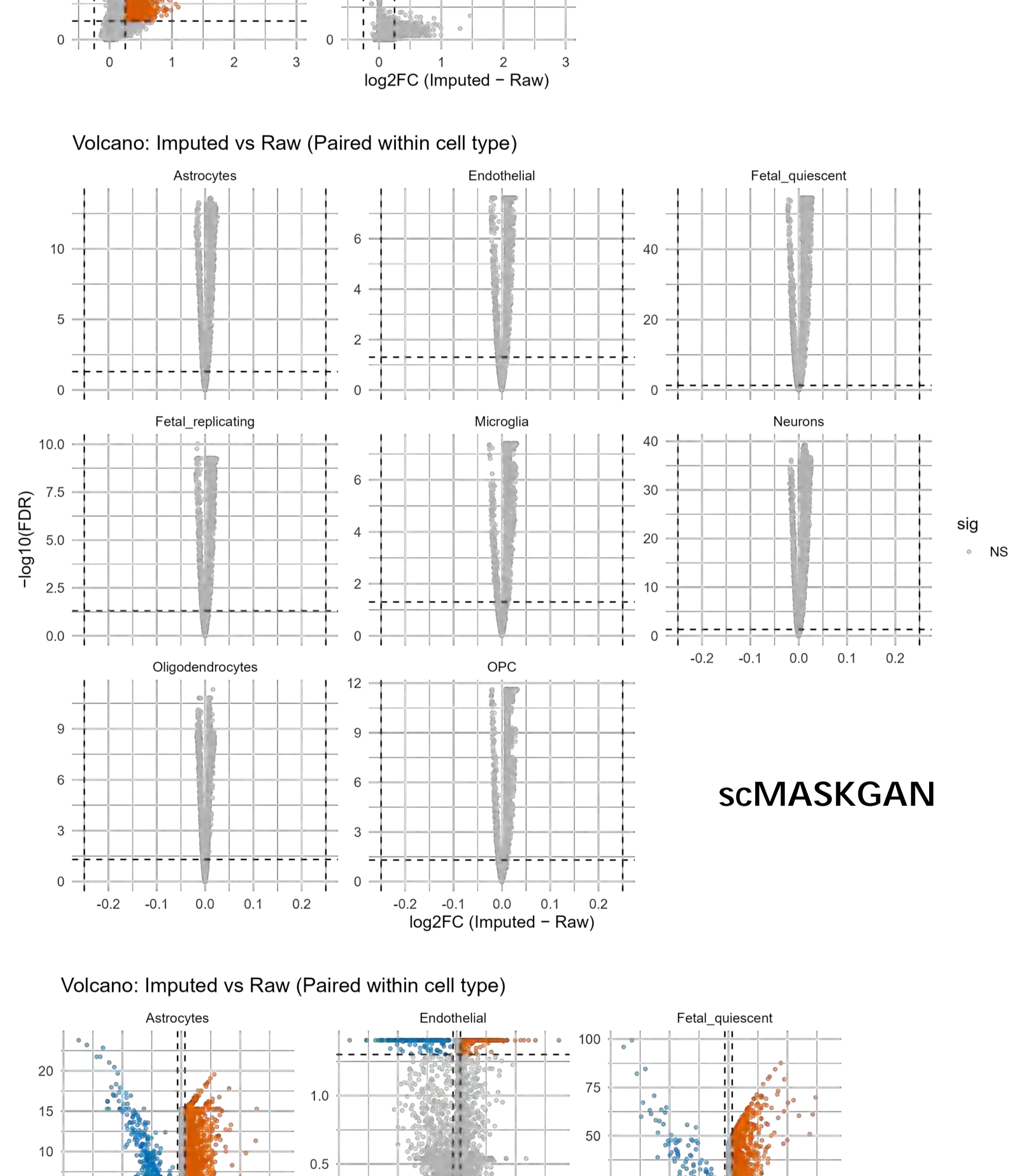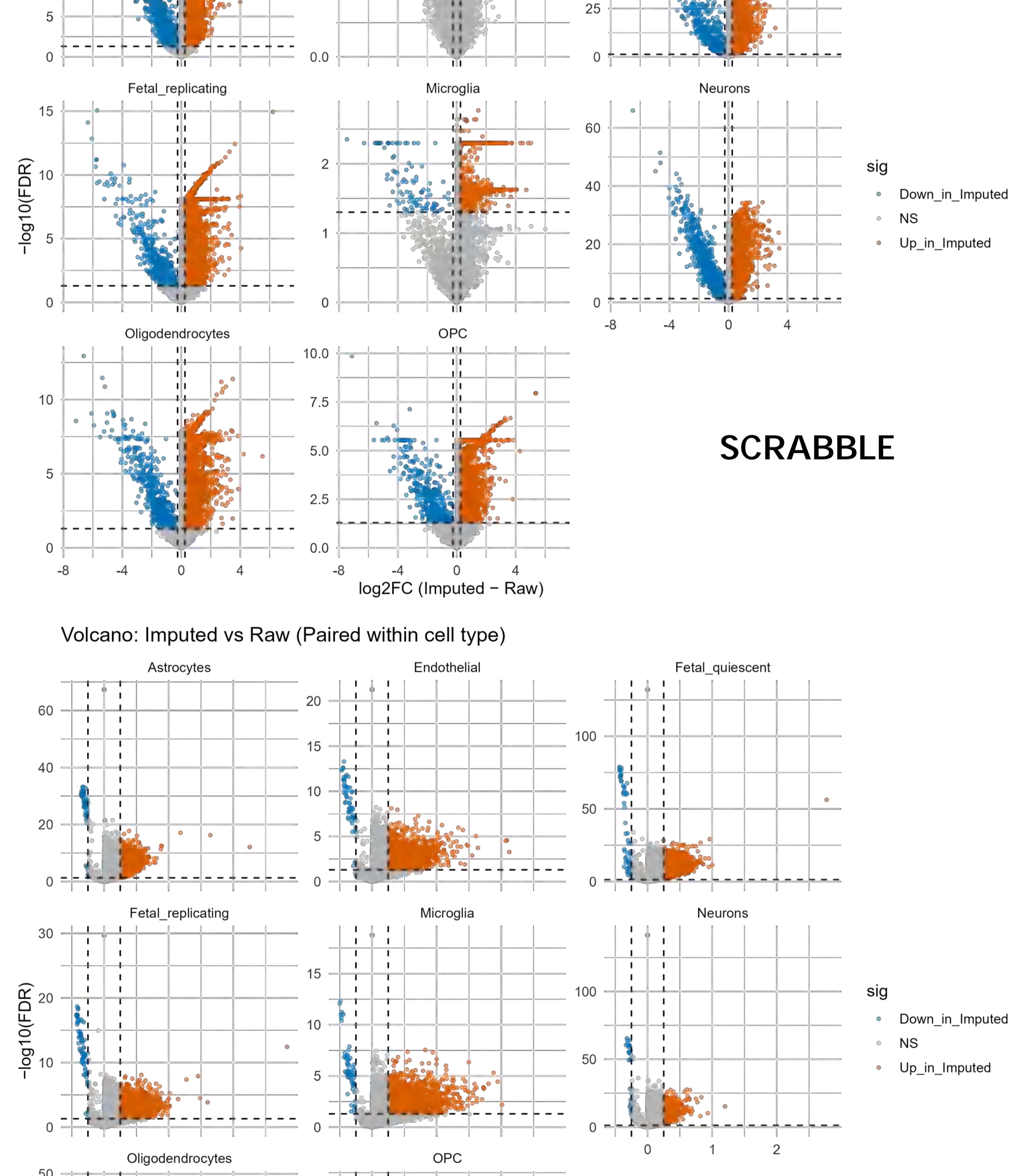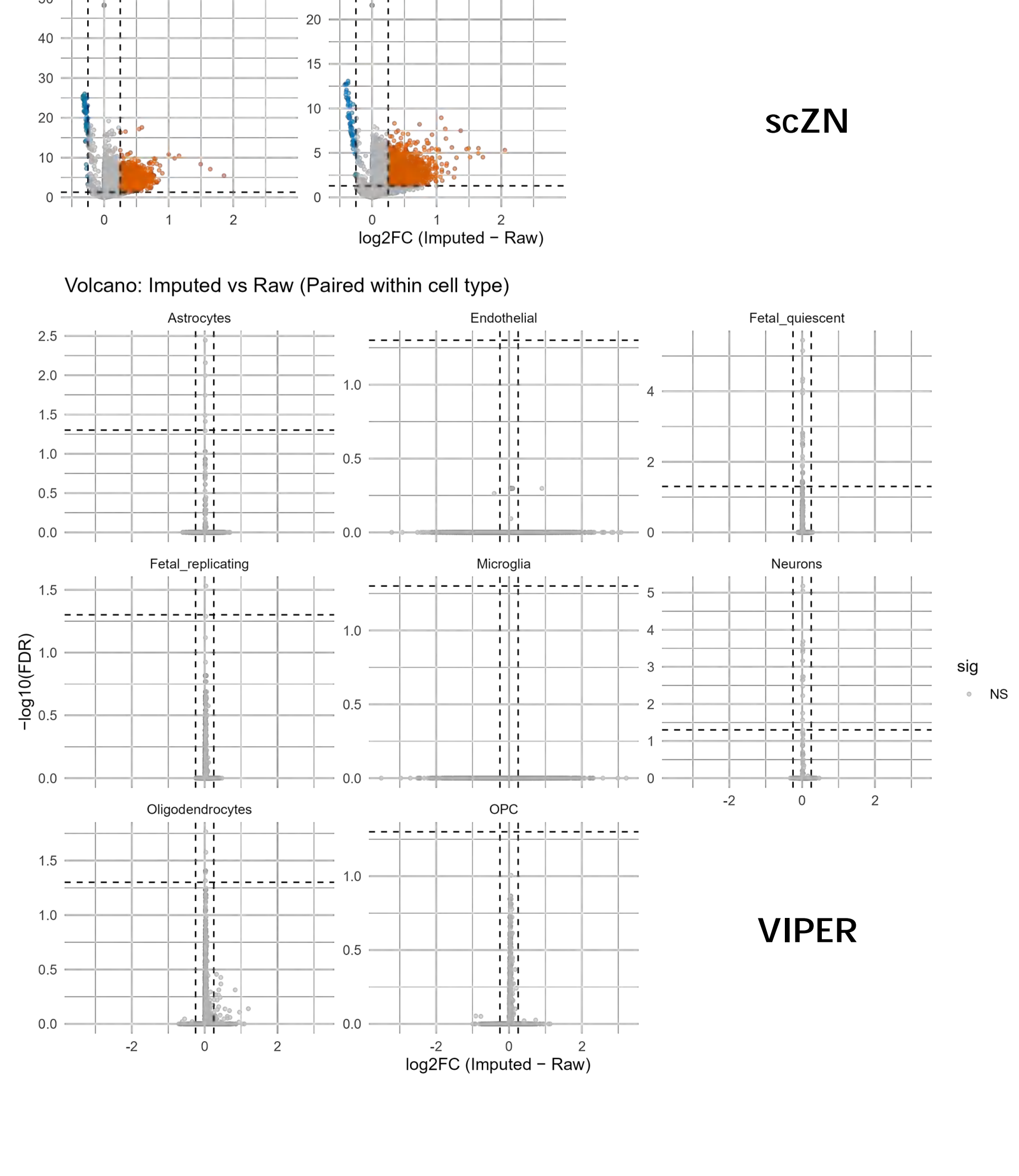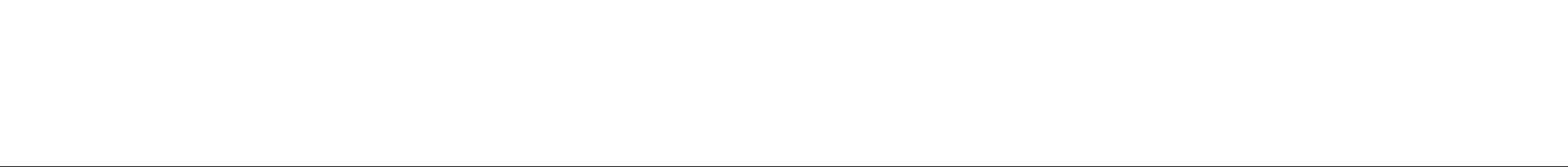

Supplement: S4 Fig — Genes significantly upregulated by multiple imputation methods. (PDF) [file pcbi.1014051.s004.pdf]

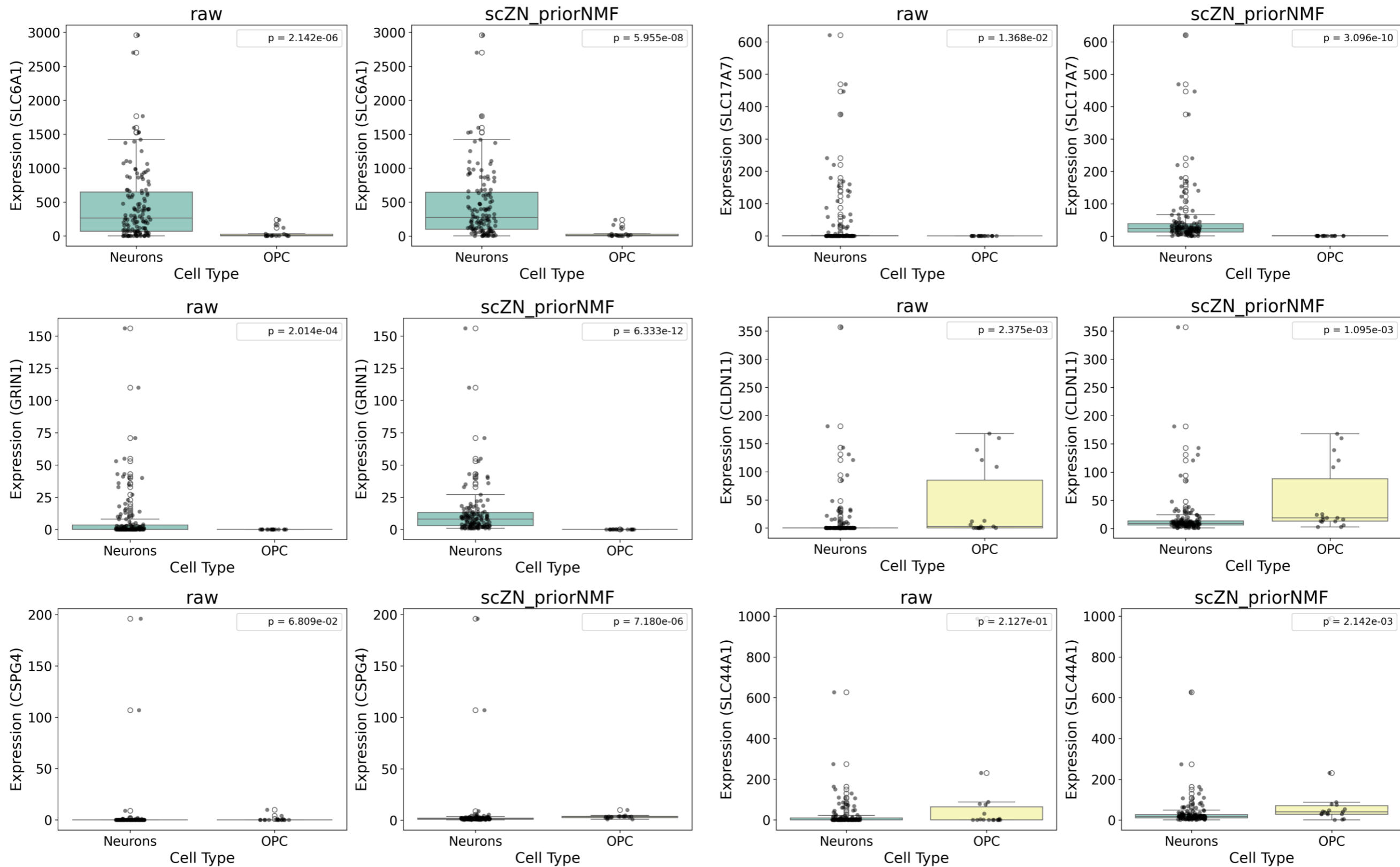

Supplement: S6 Fig — Significance comparison before and after imputation of SLC17A7, CLDN11, SLC6A1, GRIN1, CSPG4, and SLC44A1. (PDF) [file pcbi.1014051.s006.pdf]

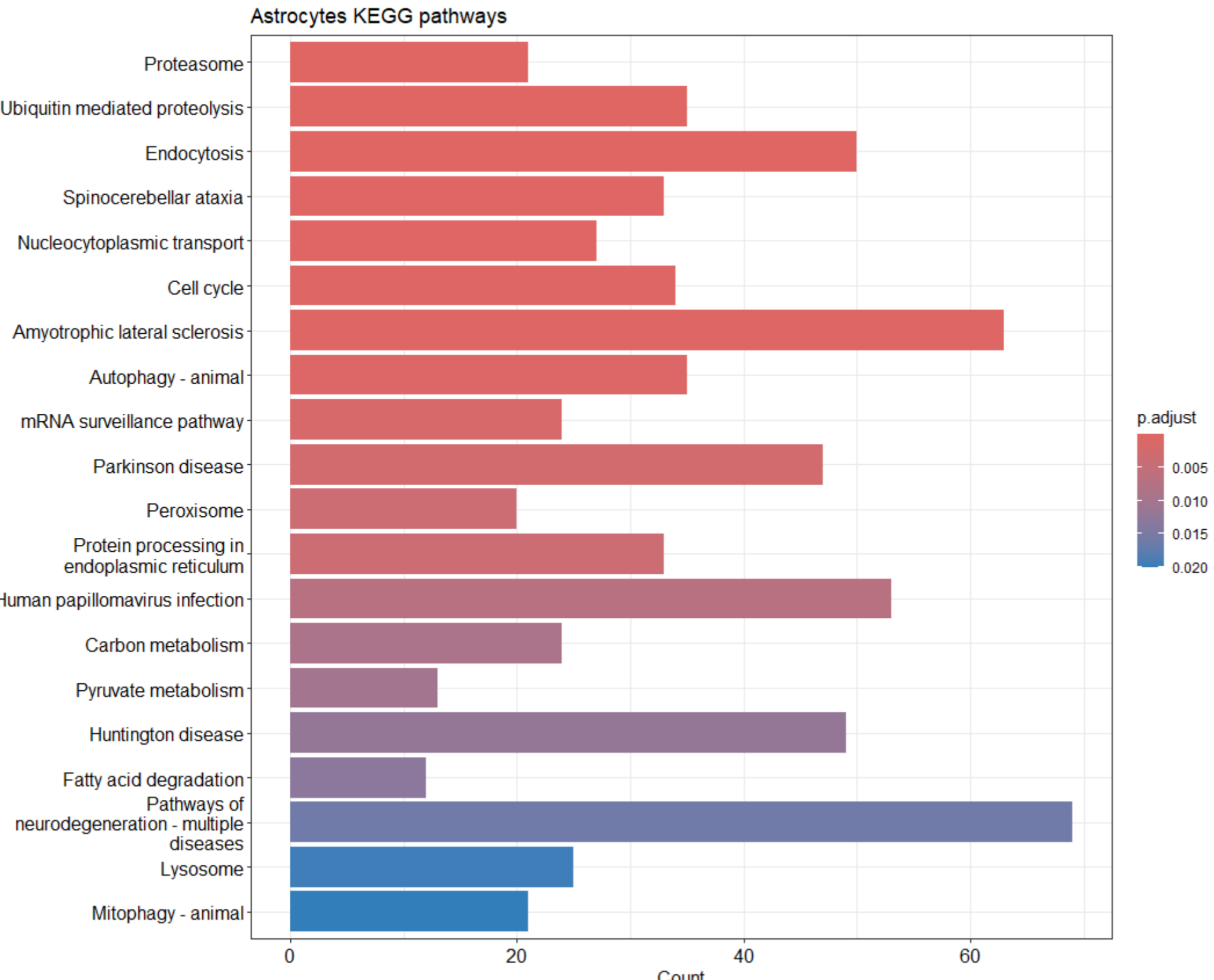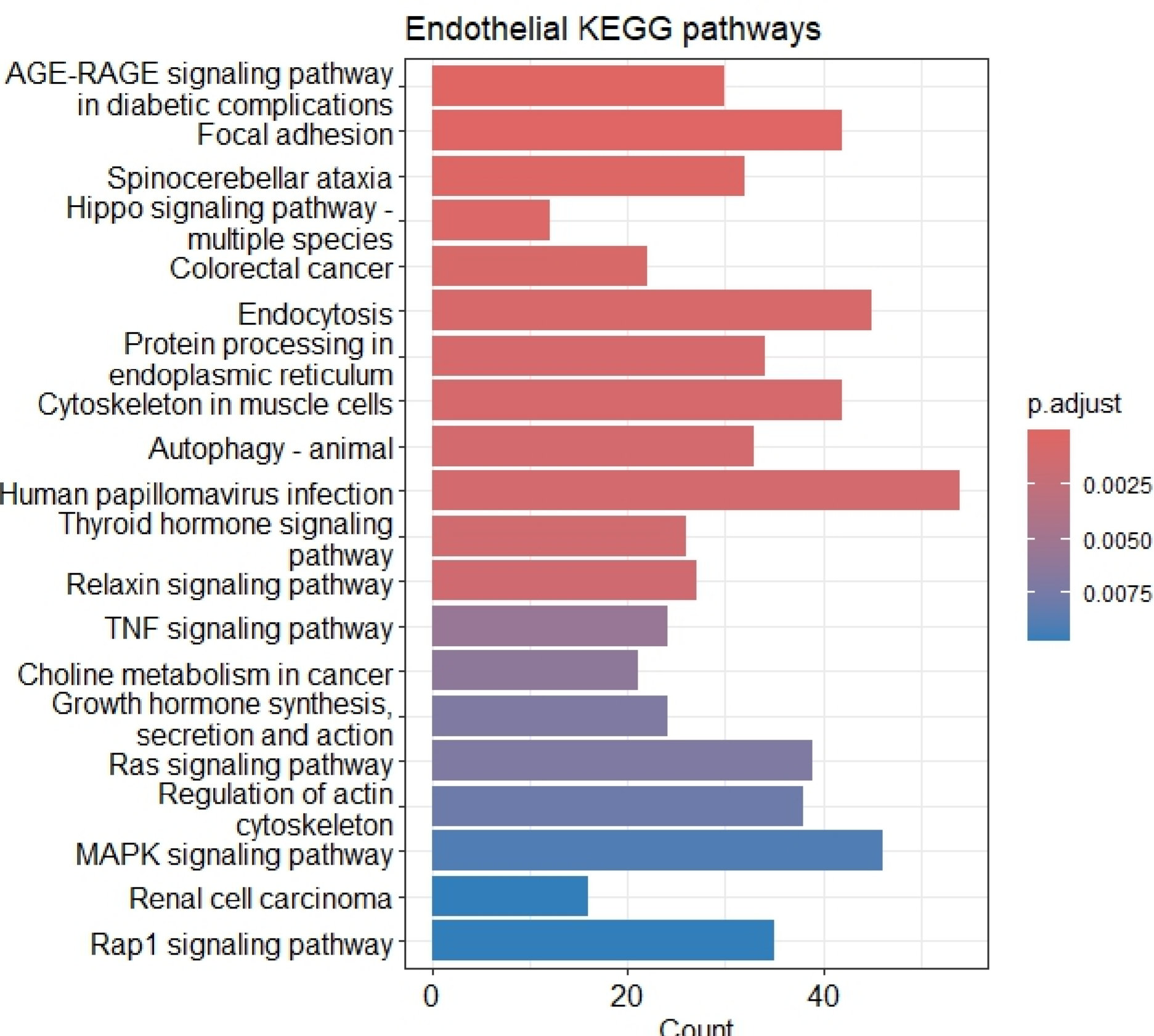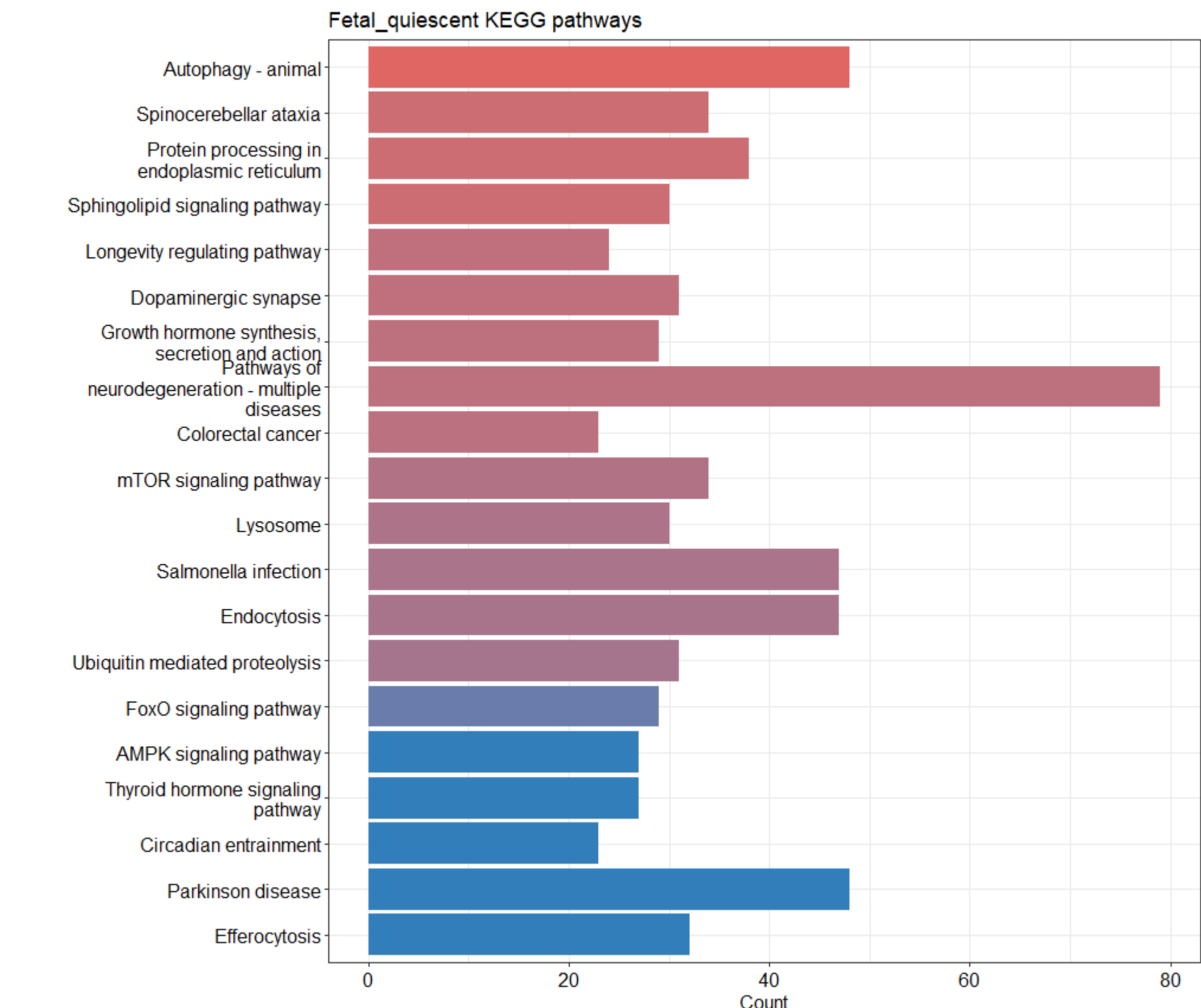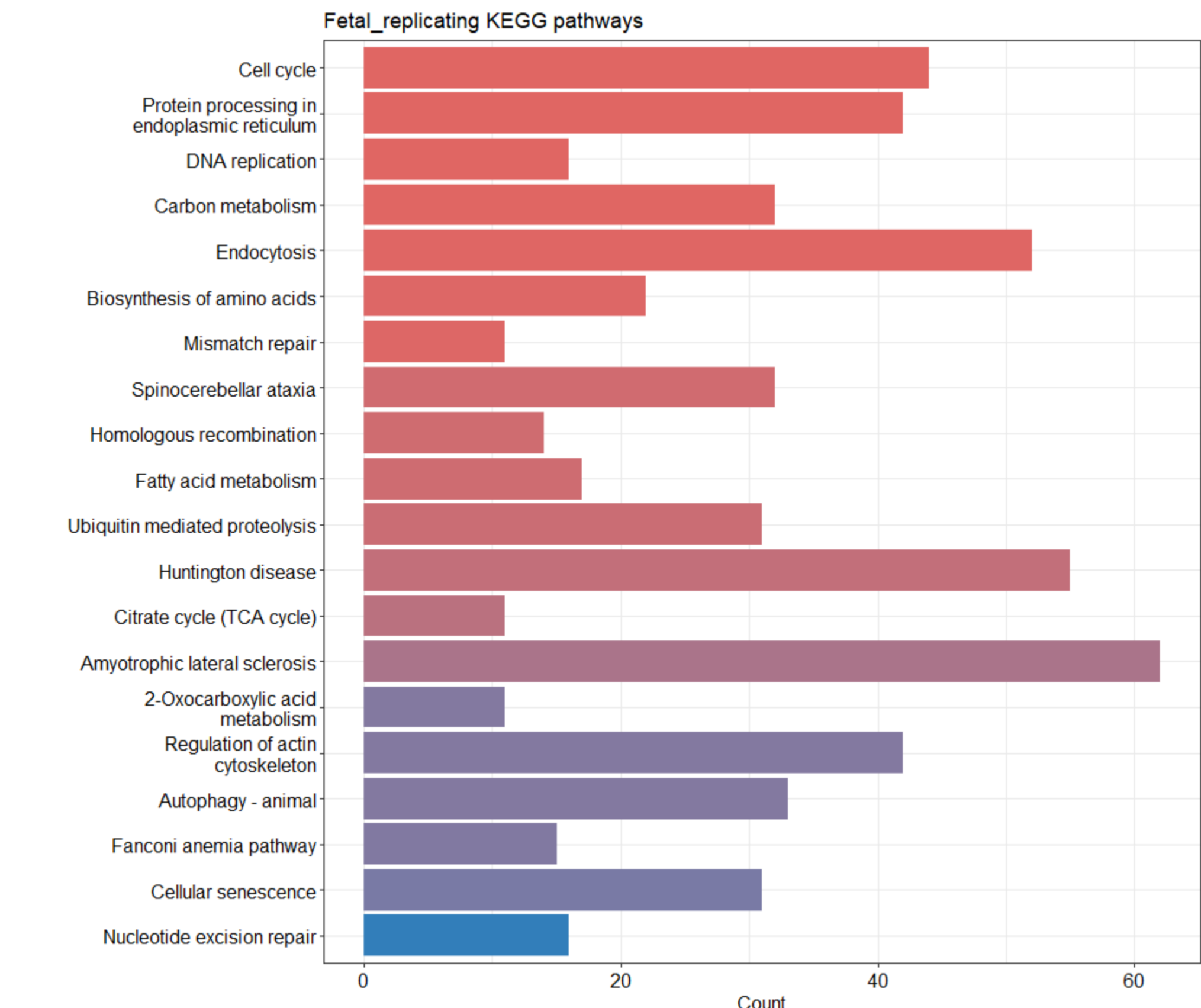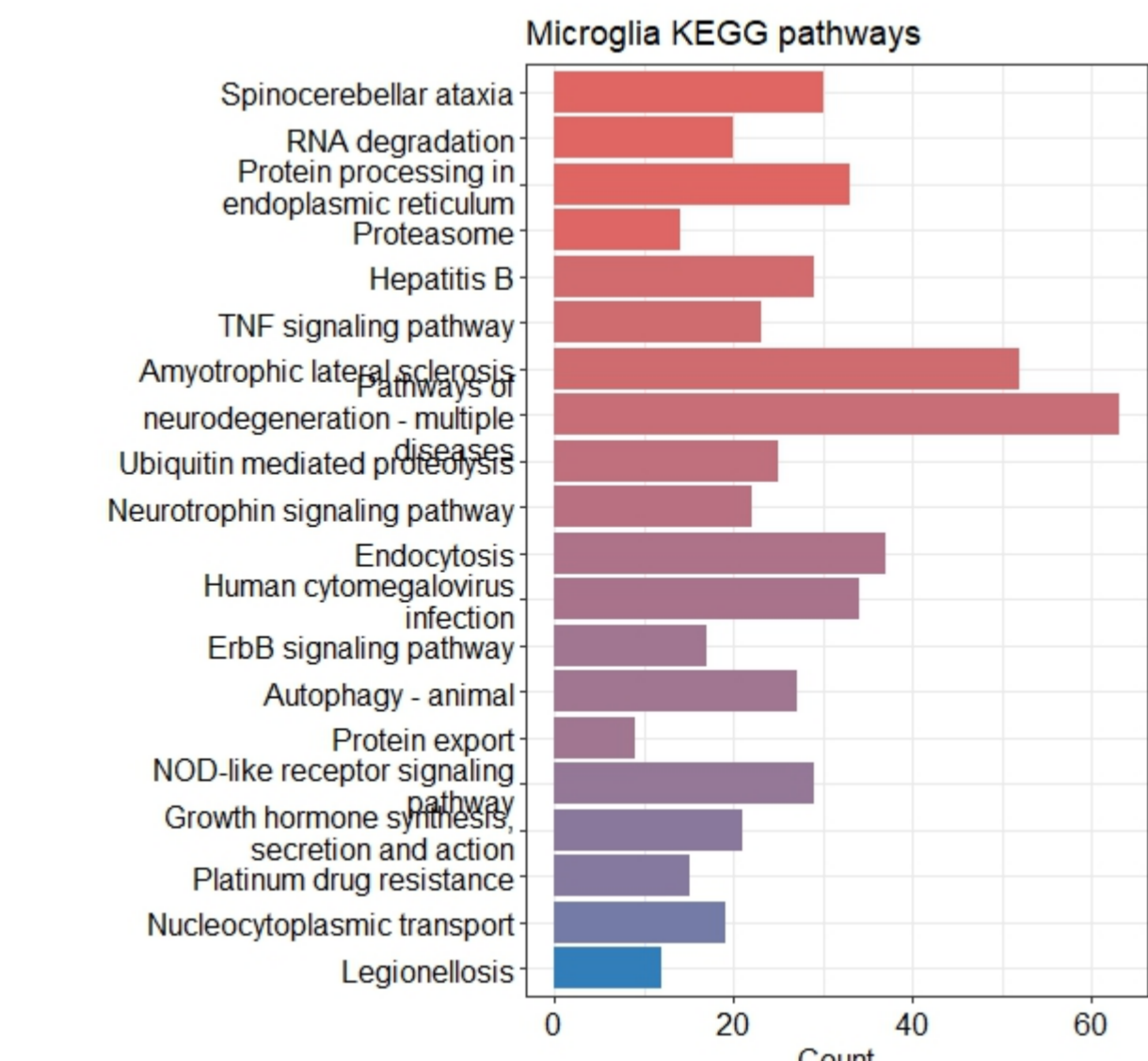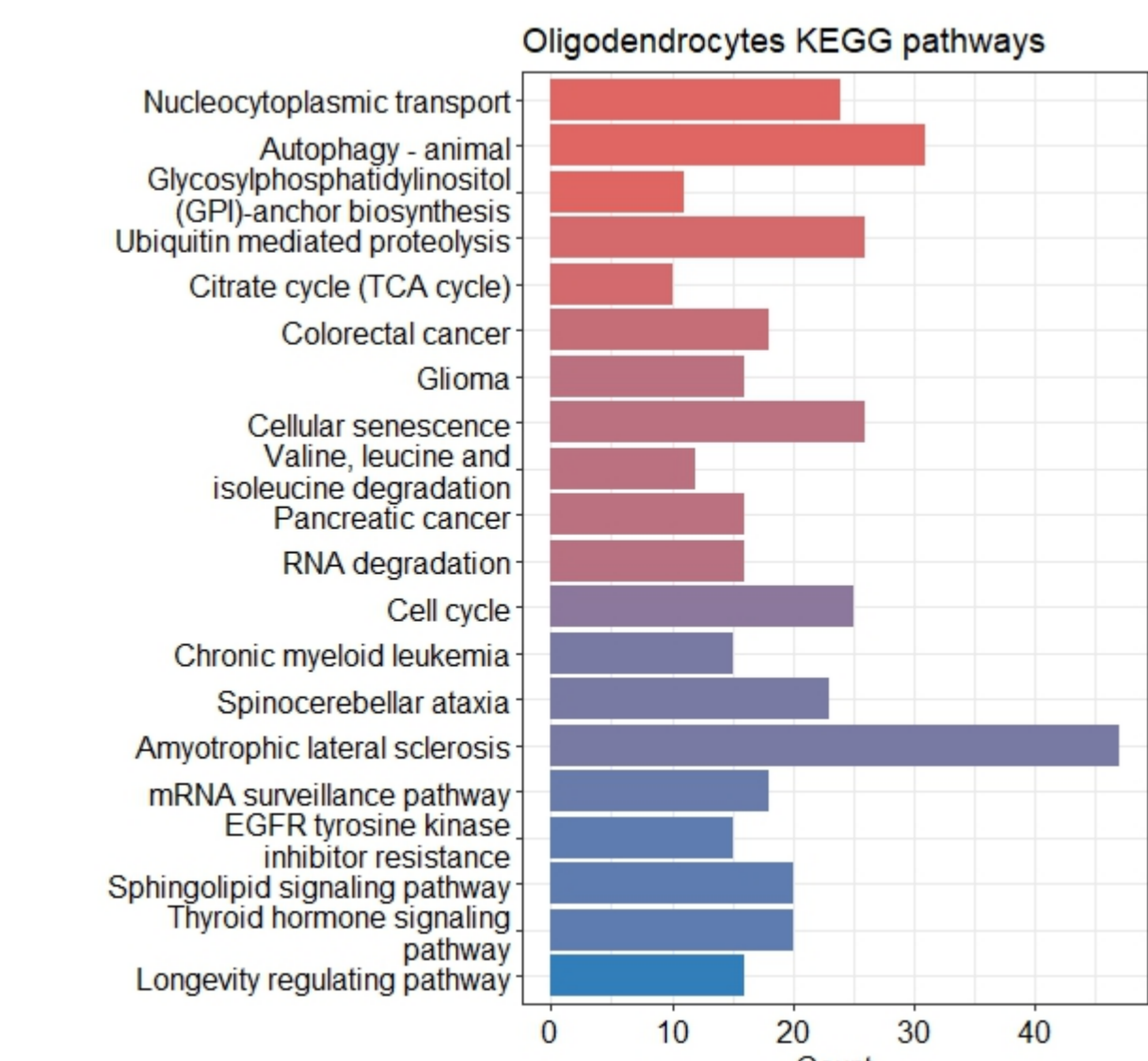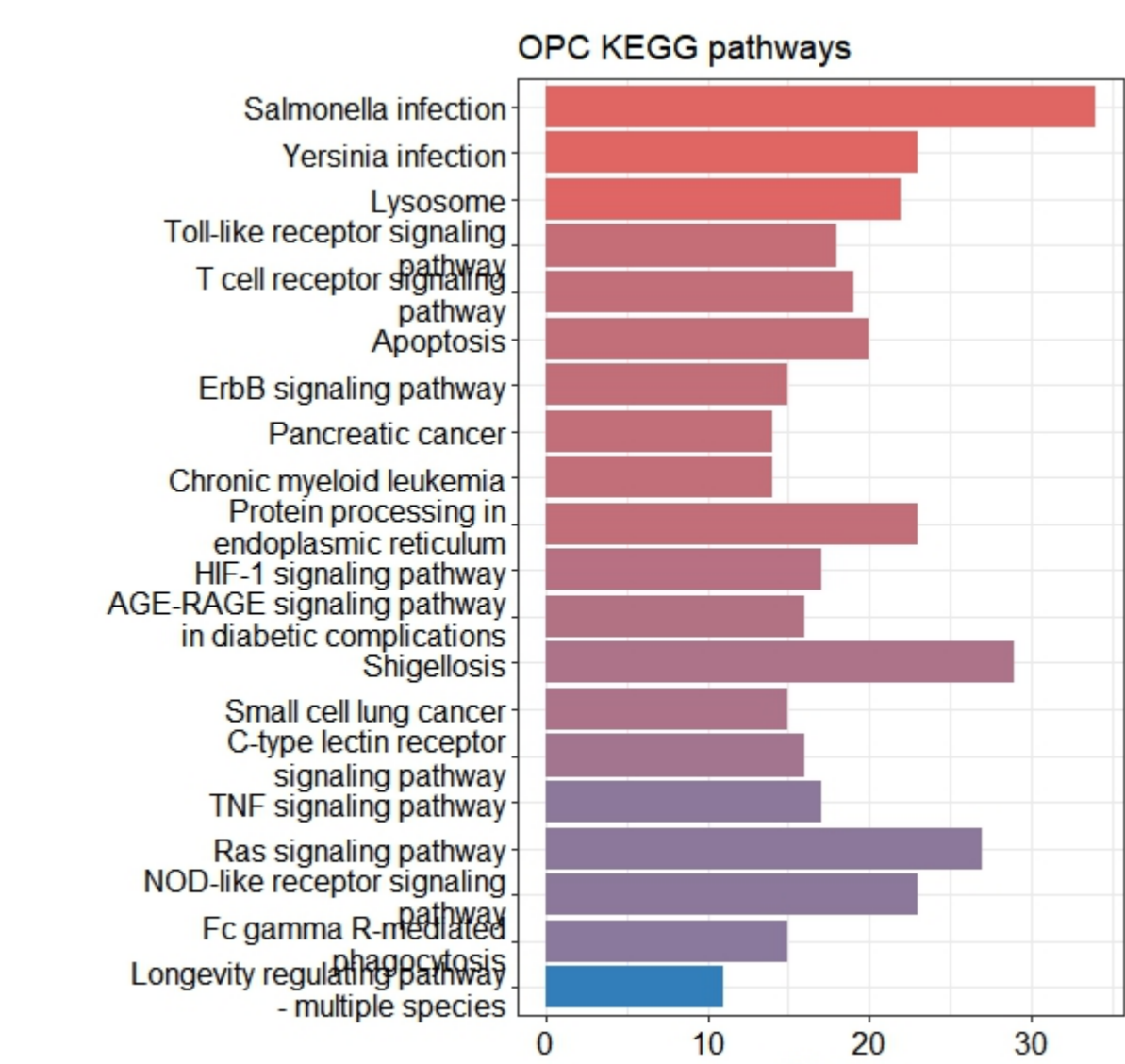

Supplement: S7 Fig — Changes in KEGG pathways of upregulated genes in human brain datasets after scZN imputation. (PDF) [file pcbi.1014051.s007.pdf]

# AutoImpute

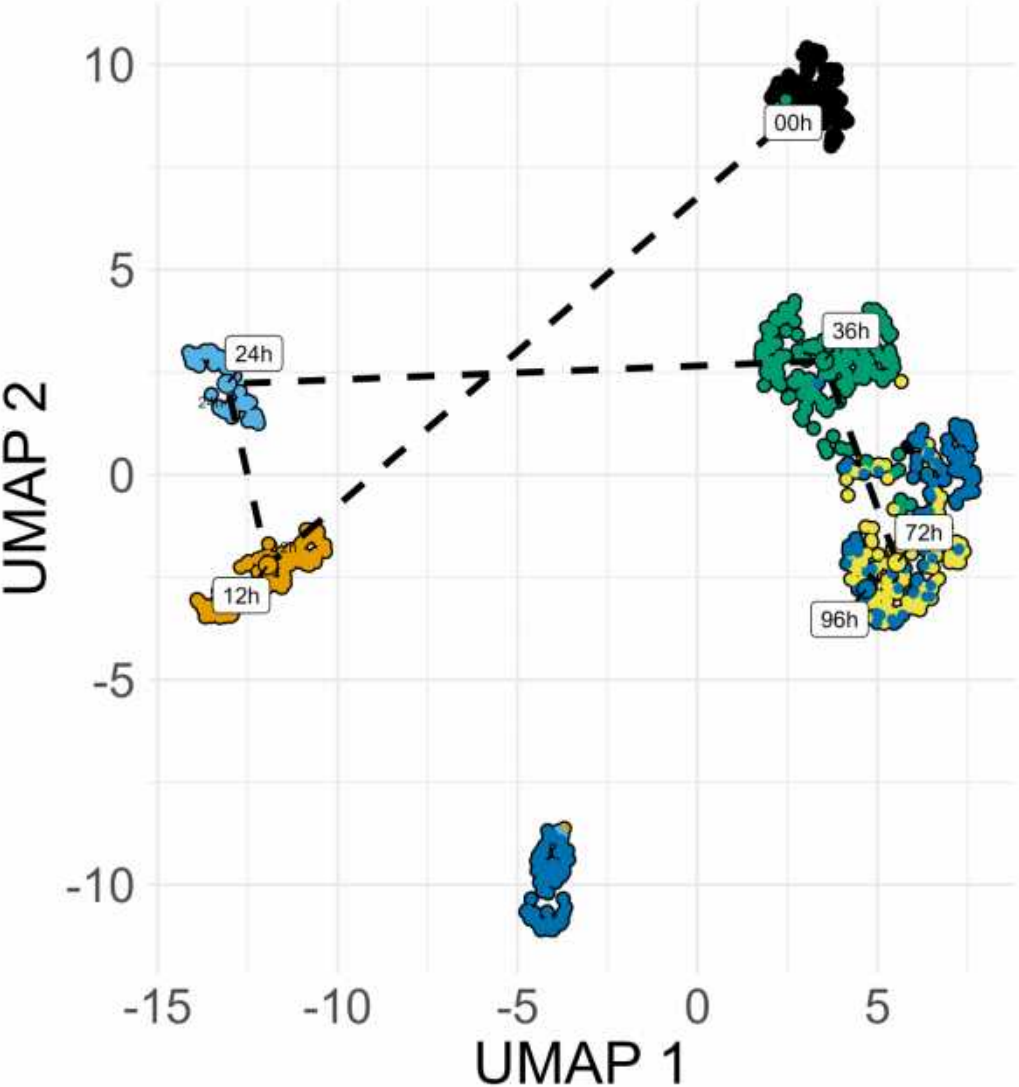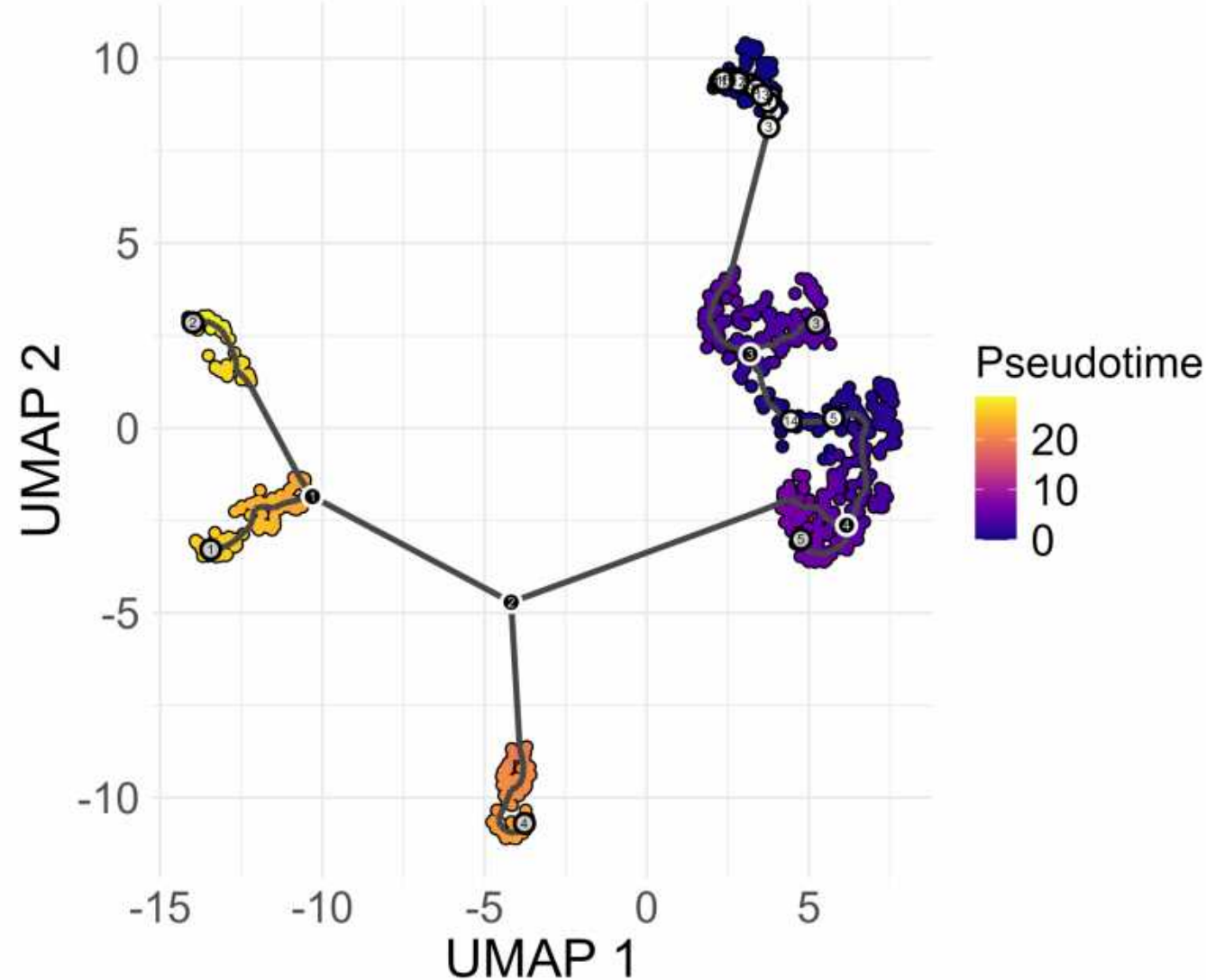

# DCA

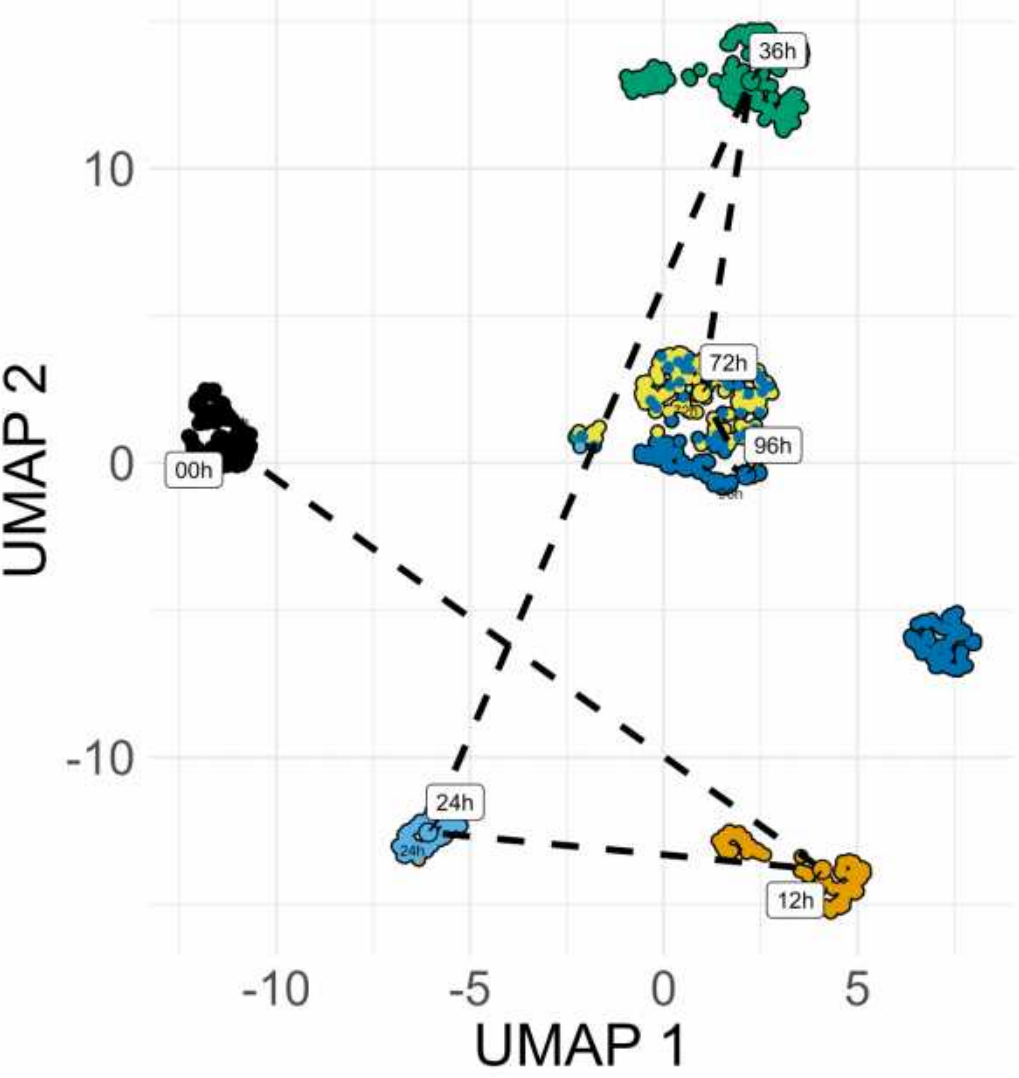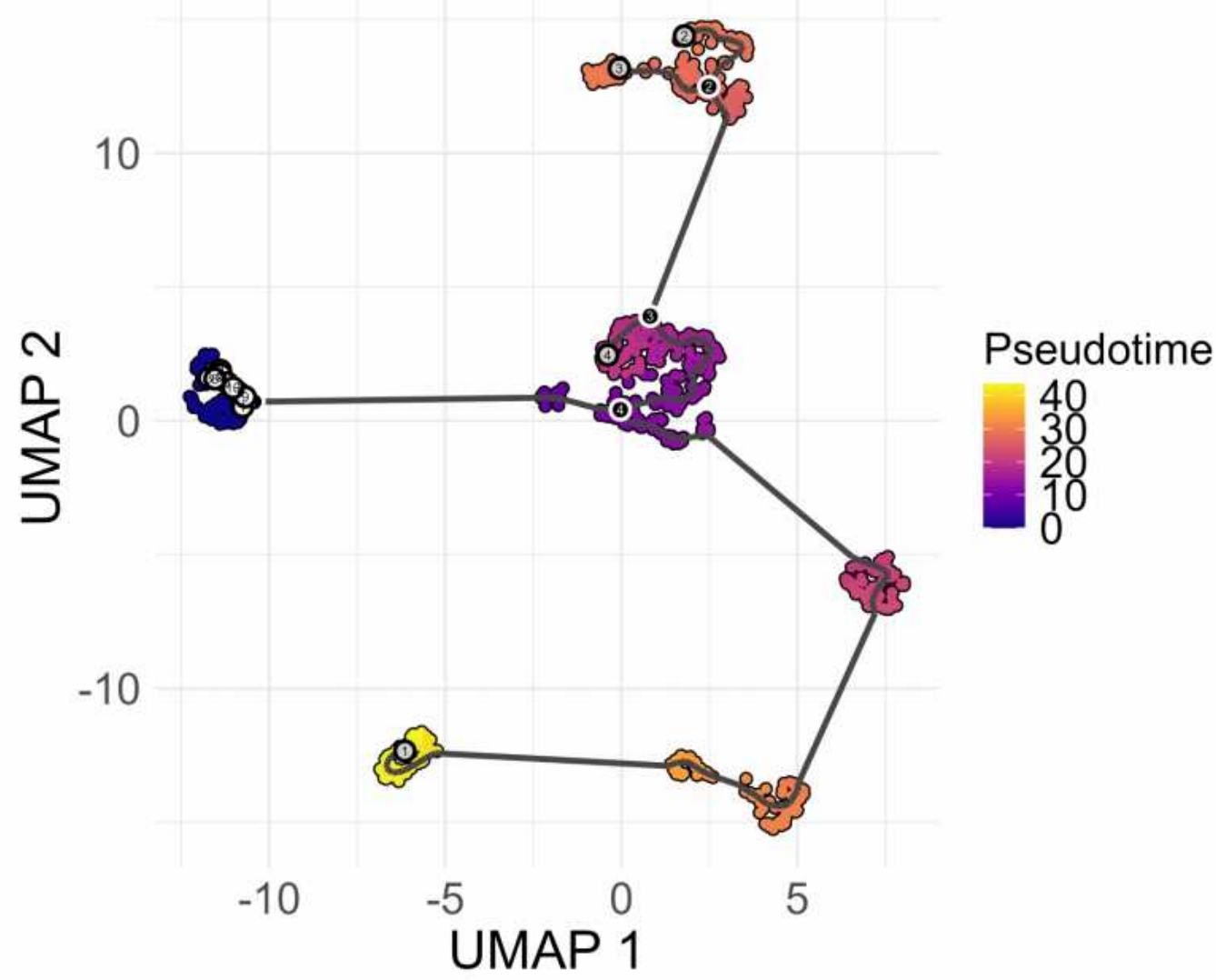

# DeepImpute

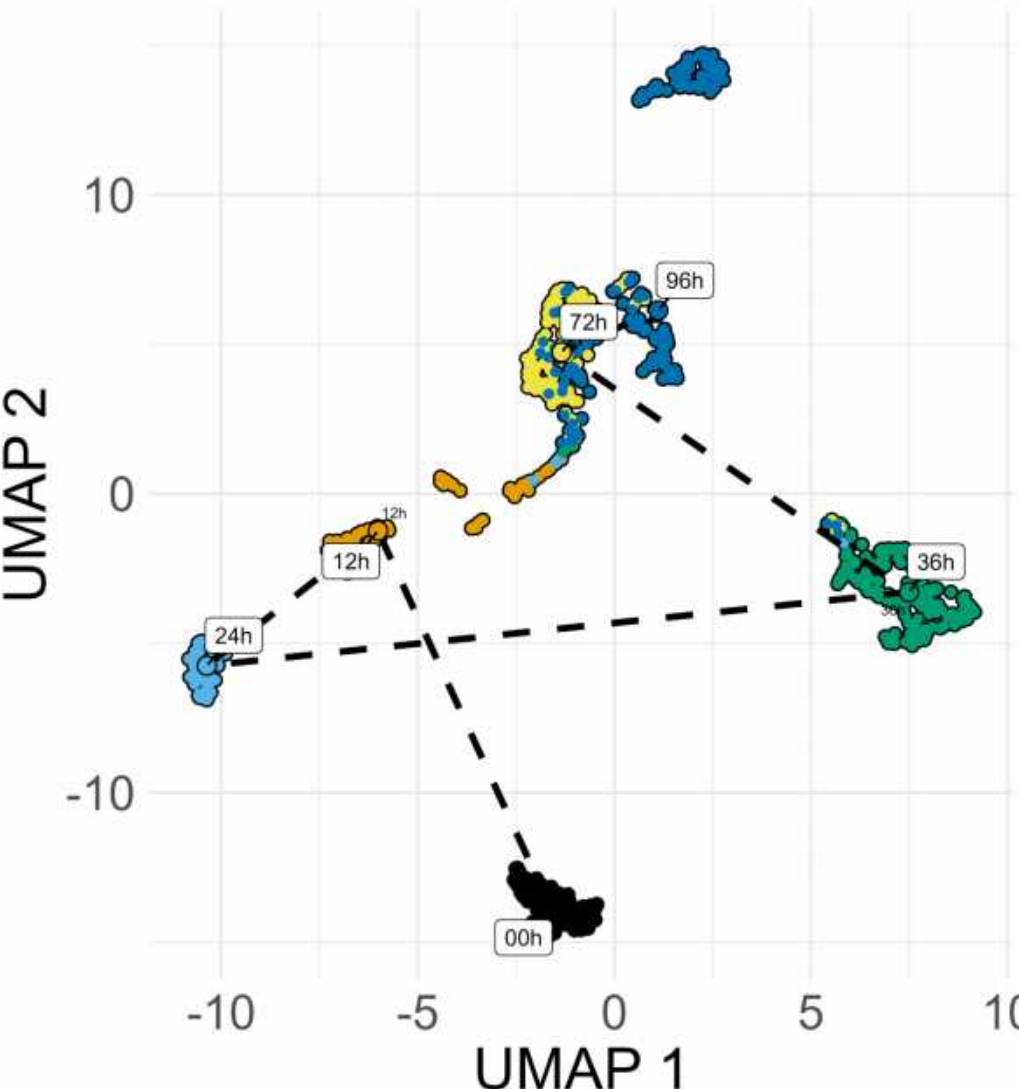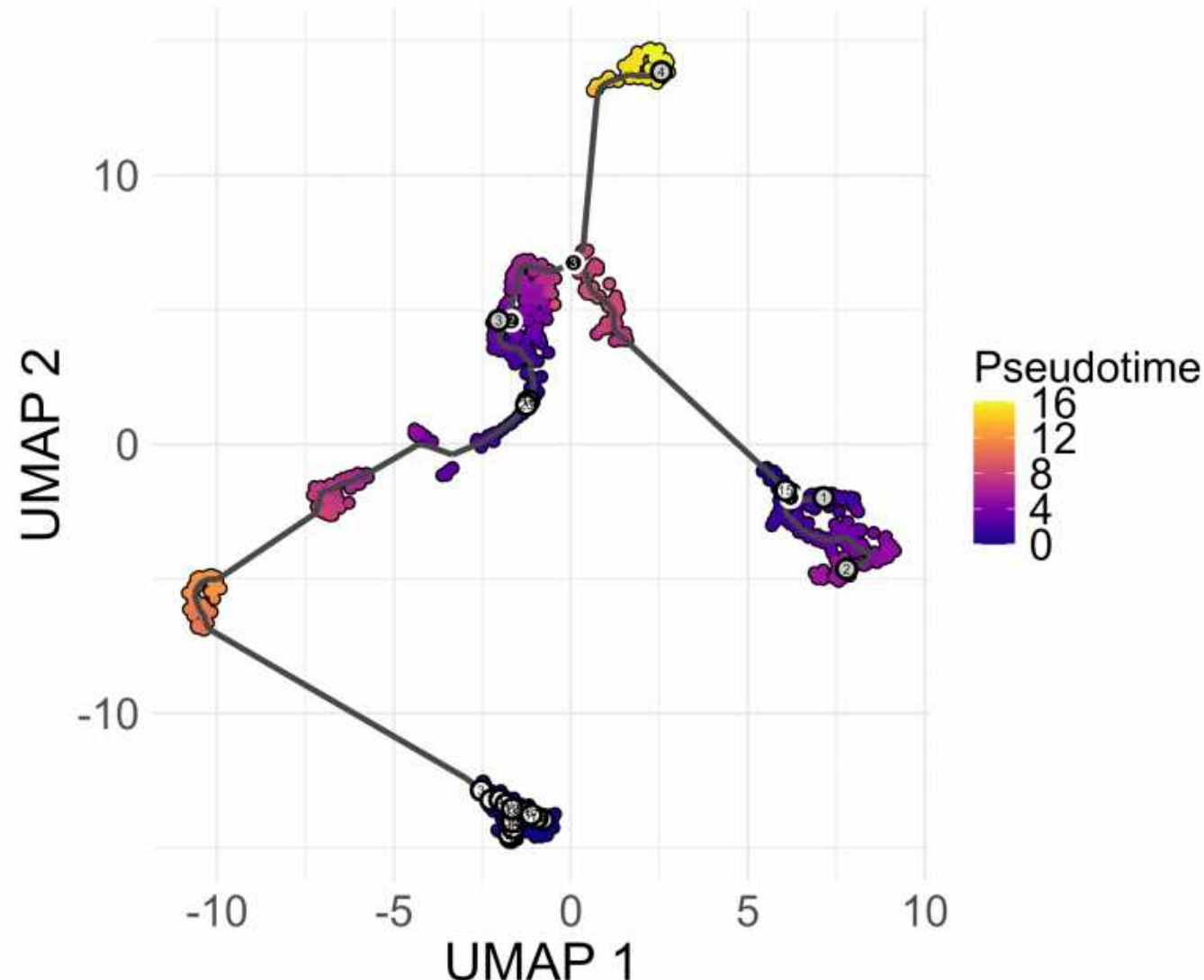

# MAGIC

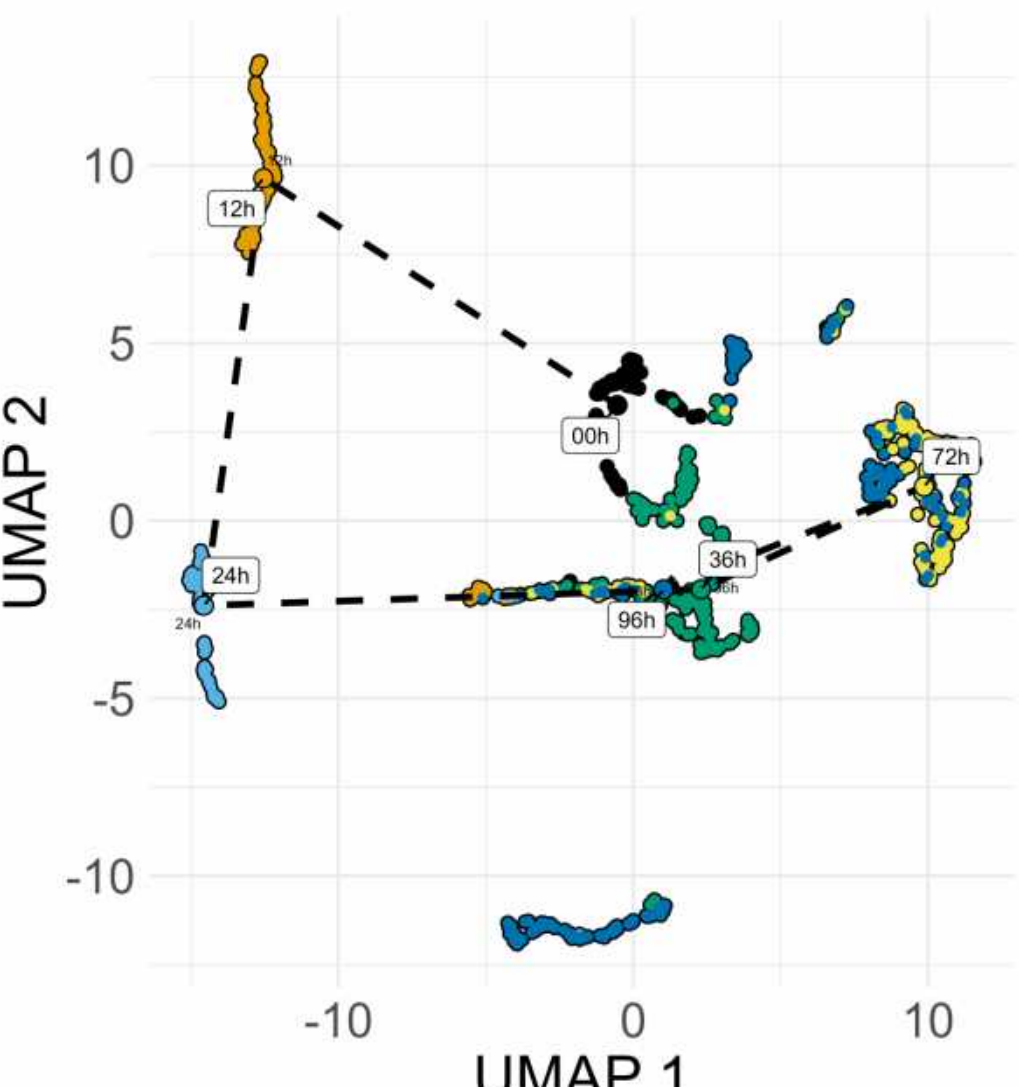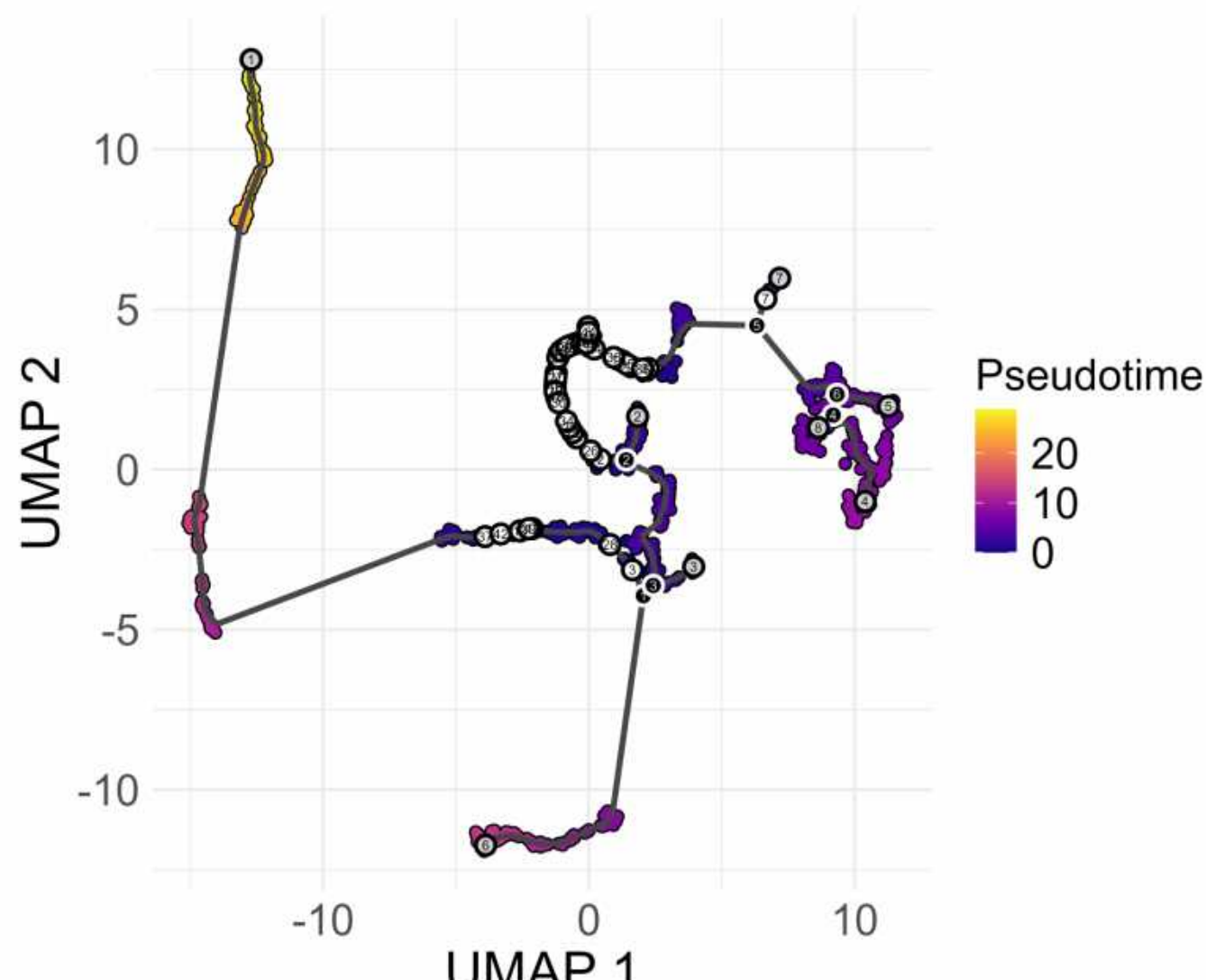

# scGAIN

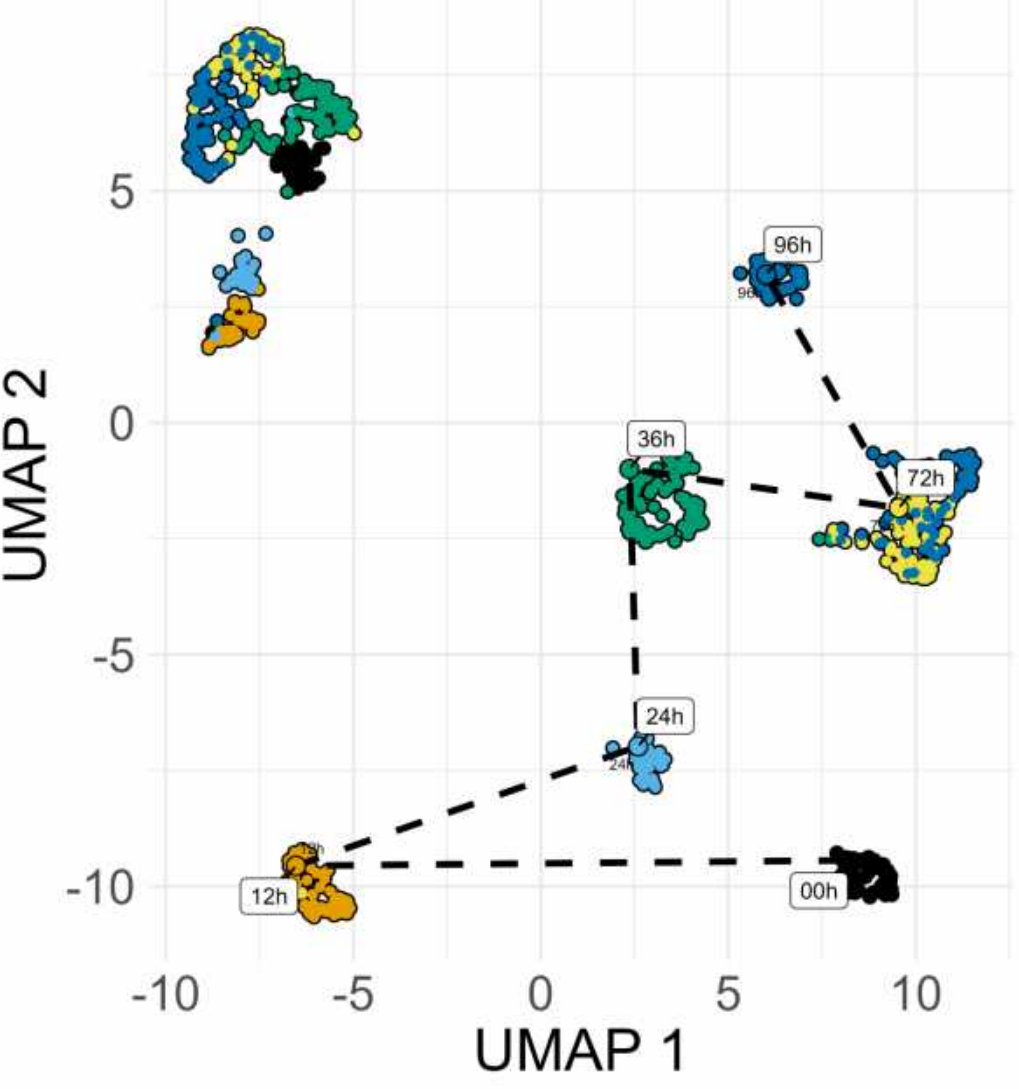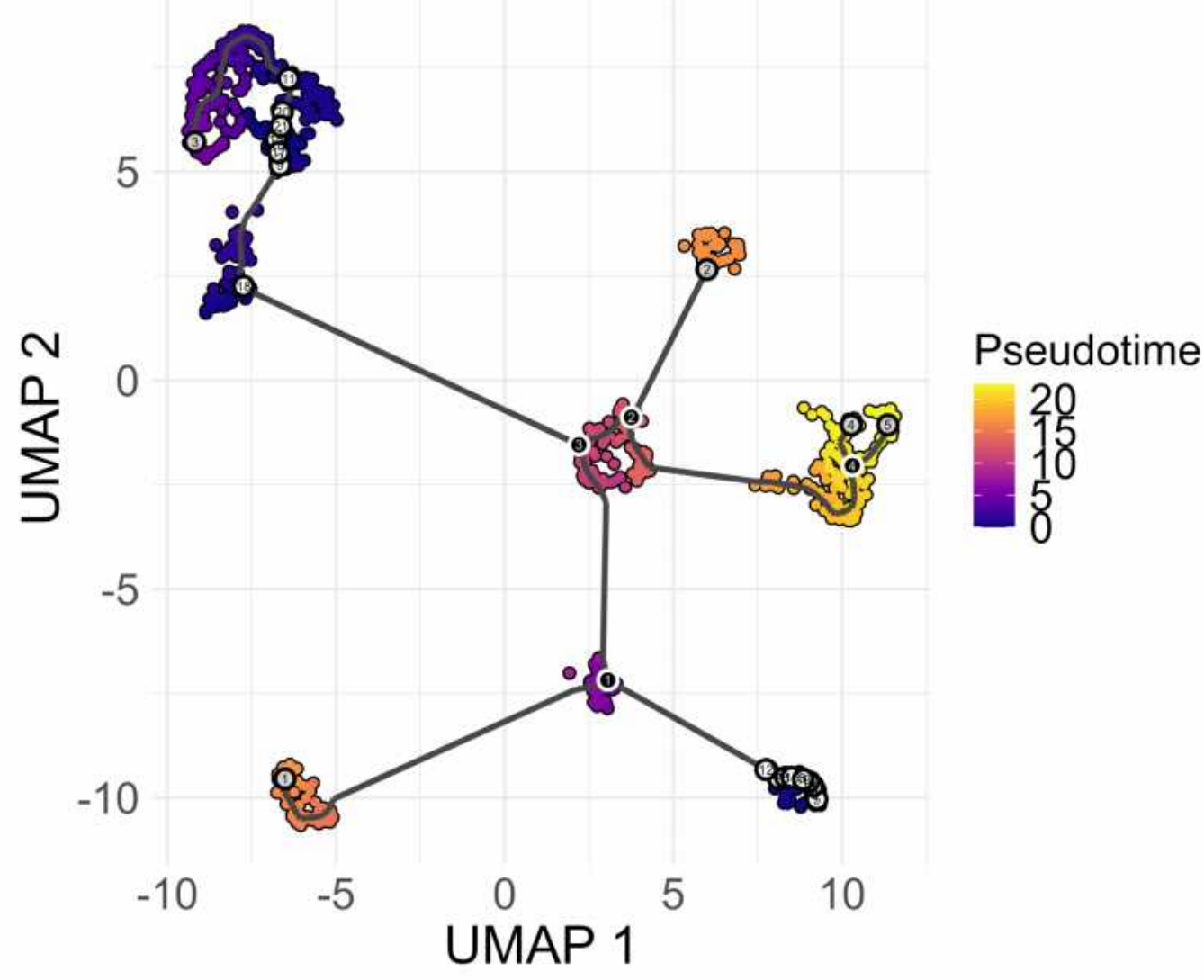

# scIGANs

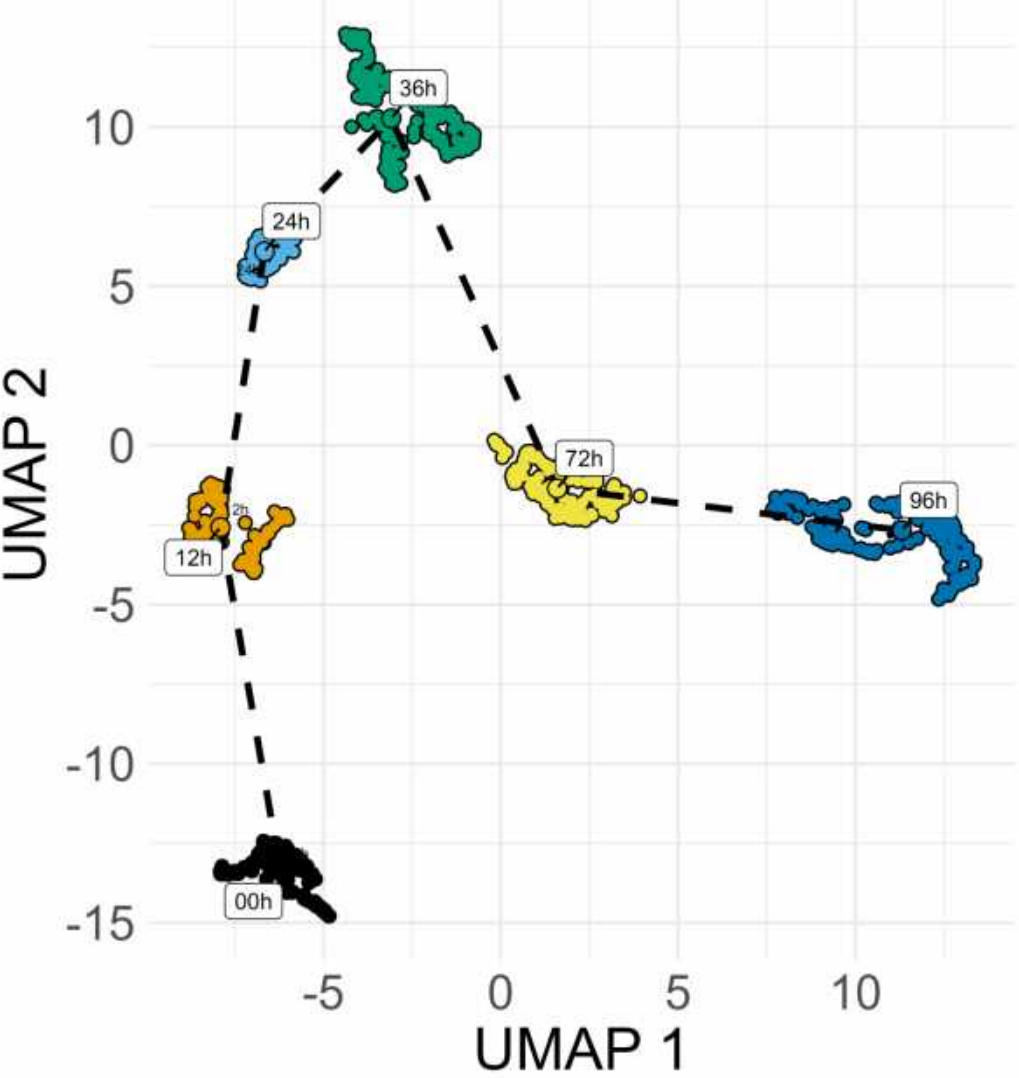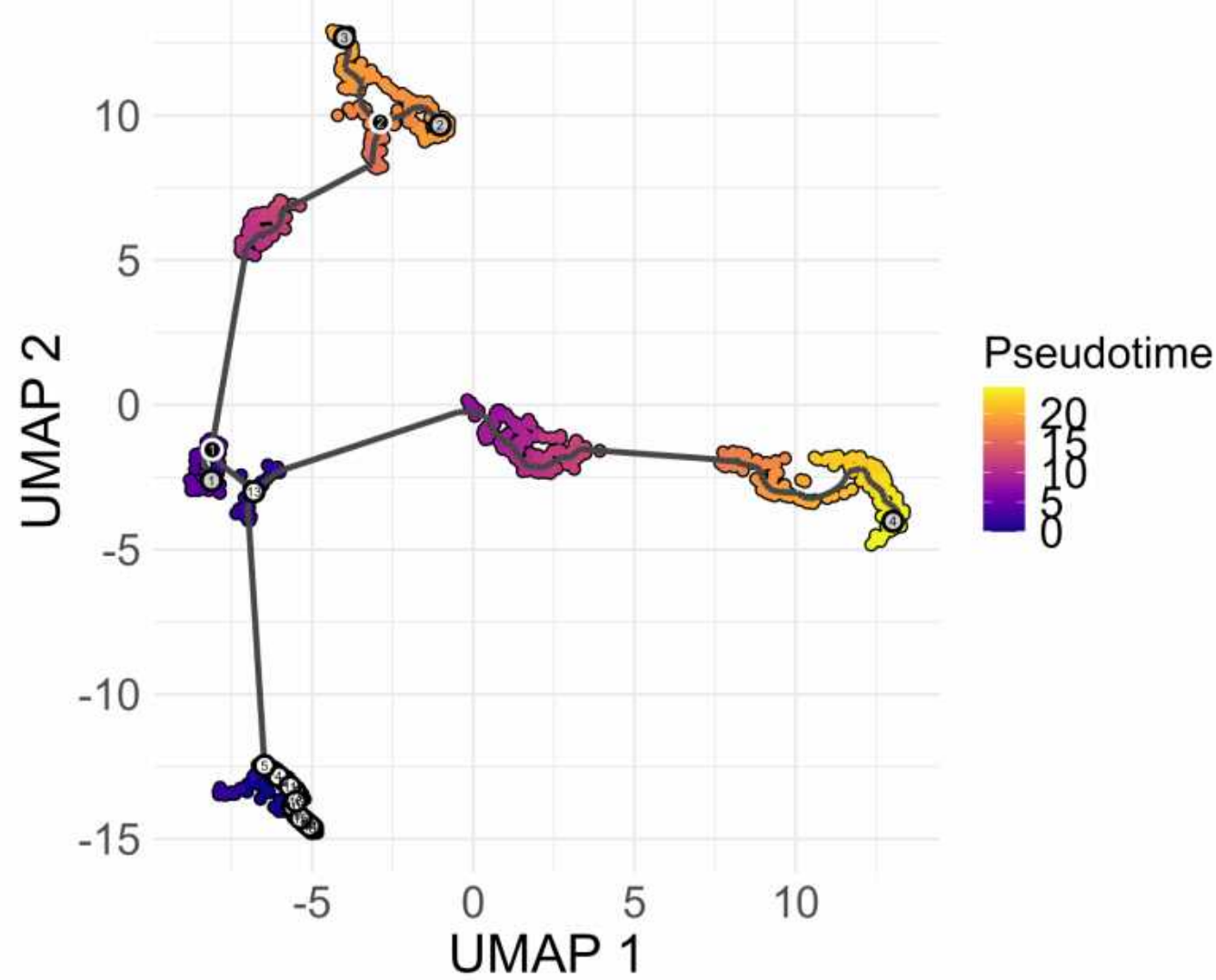

# scImpute

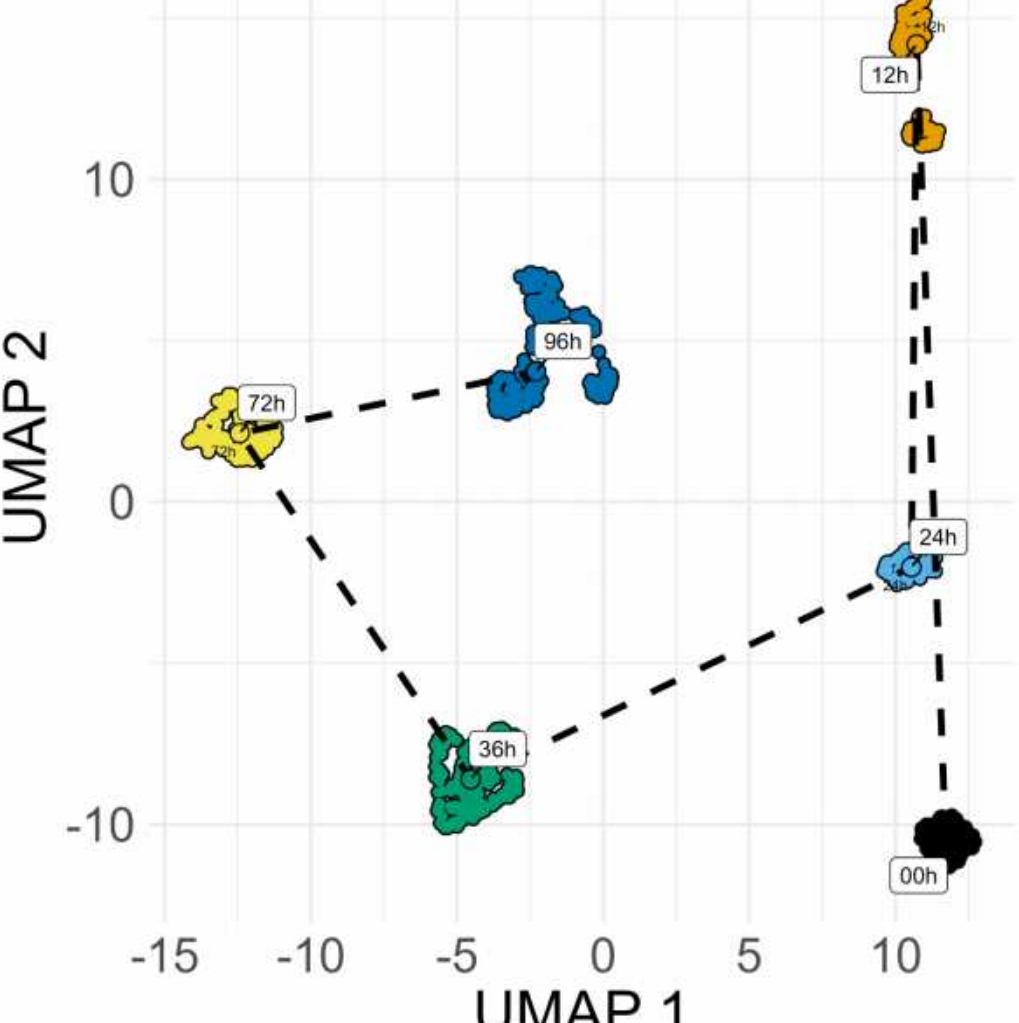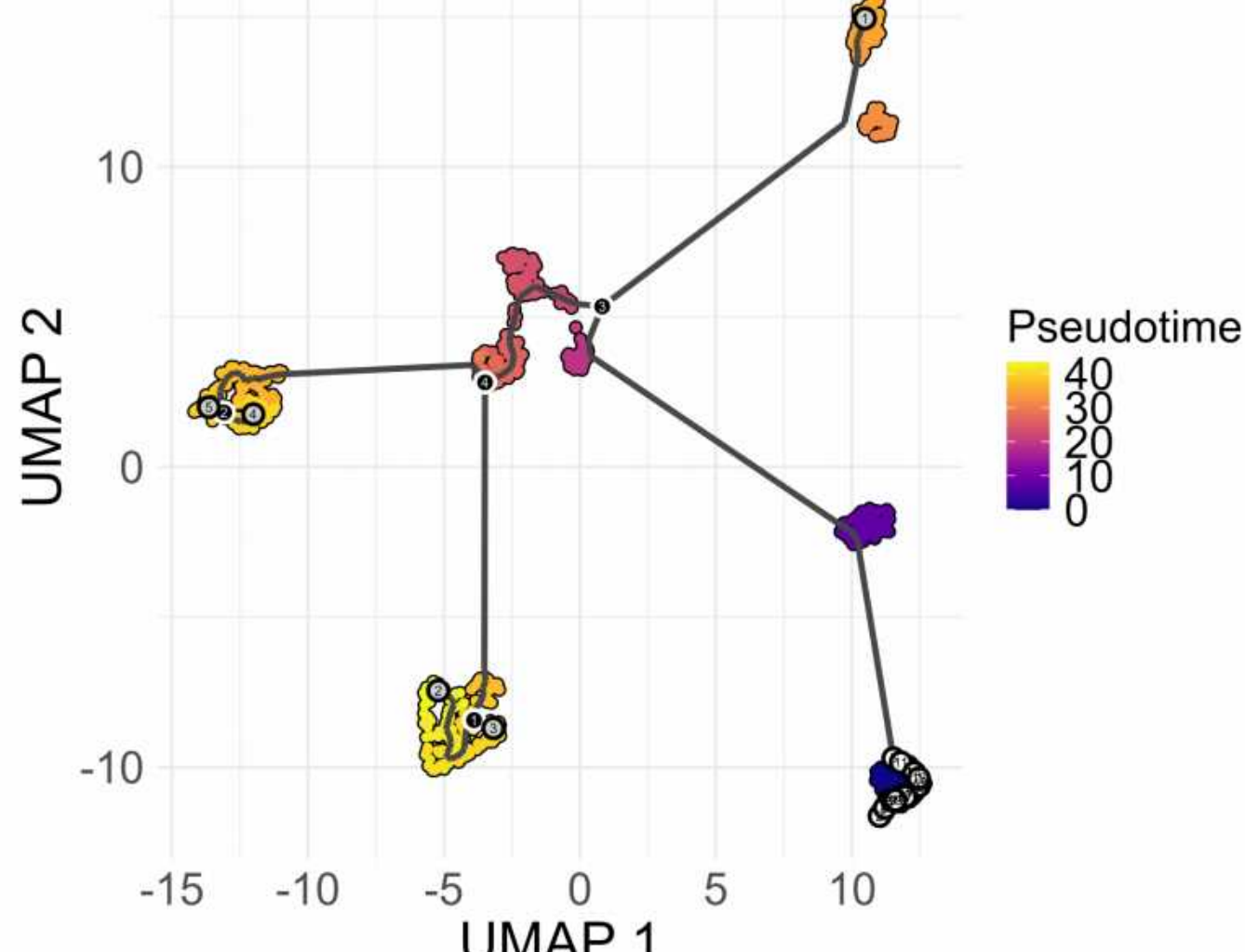

# scMASKGAN

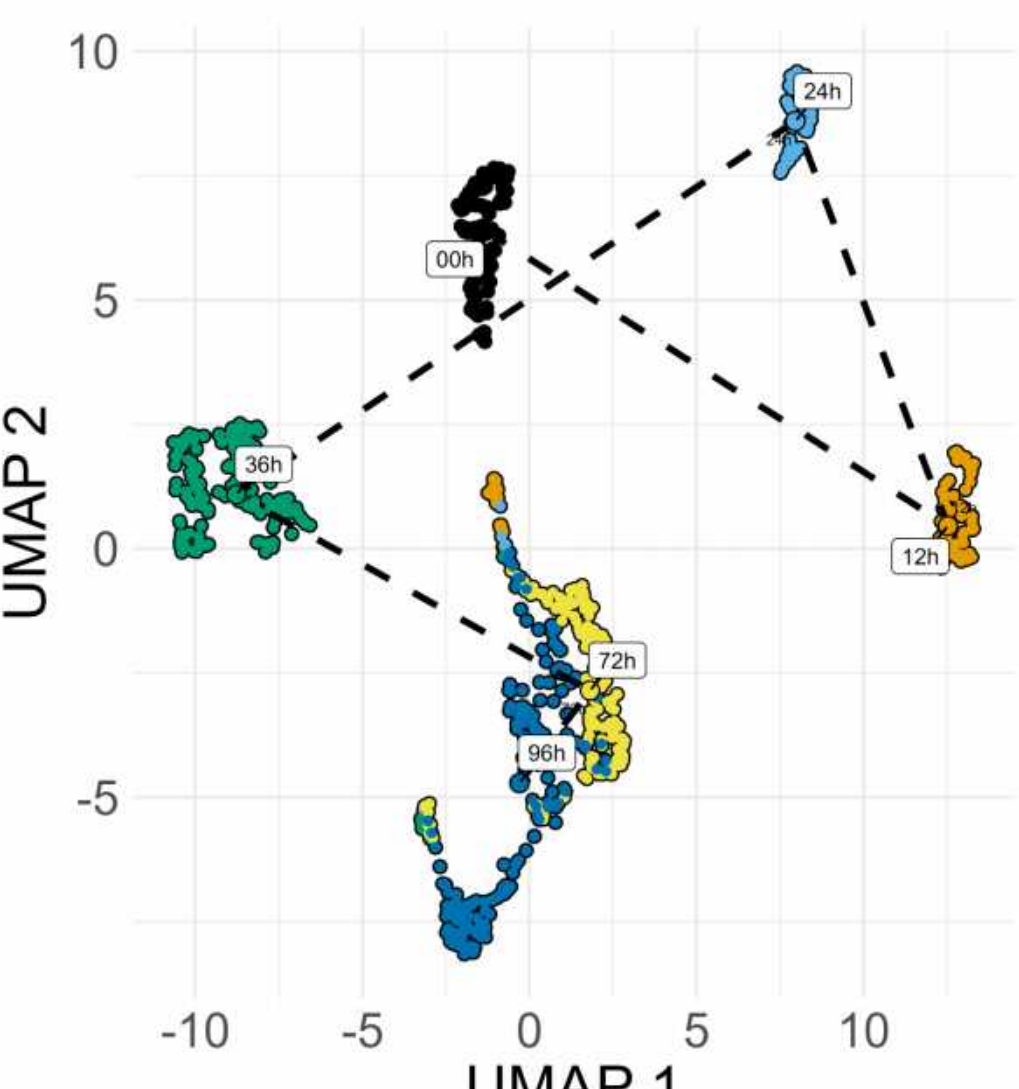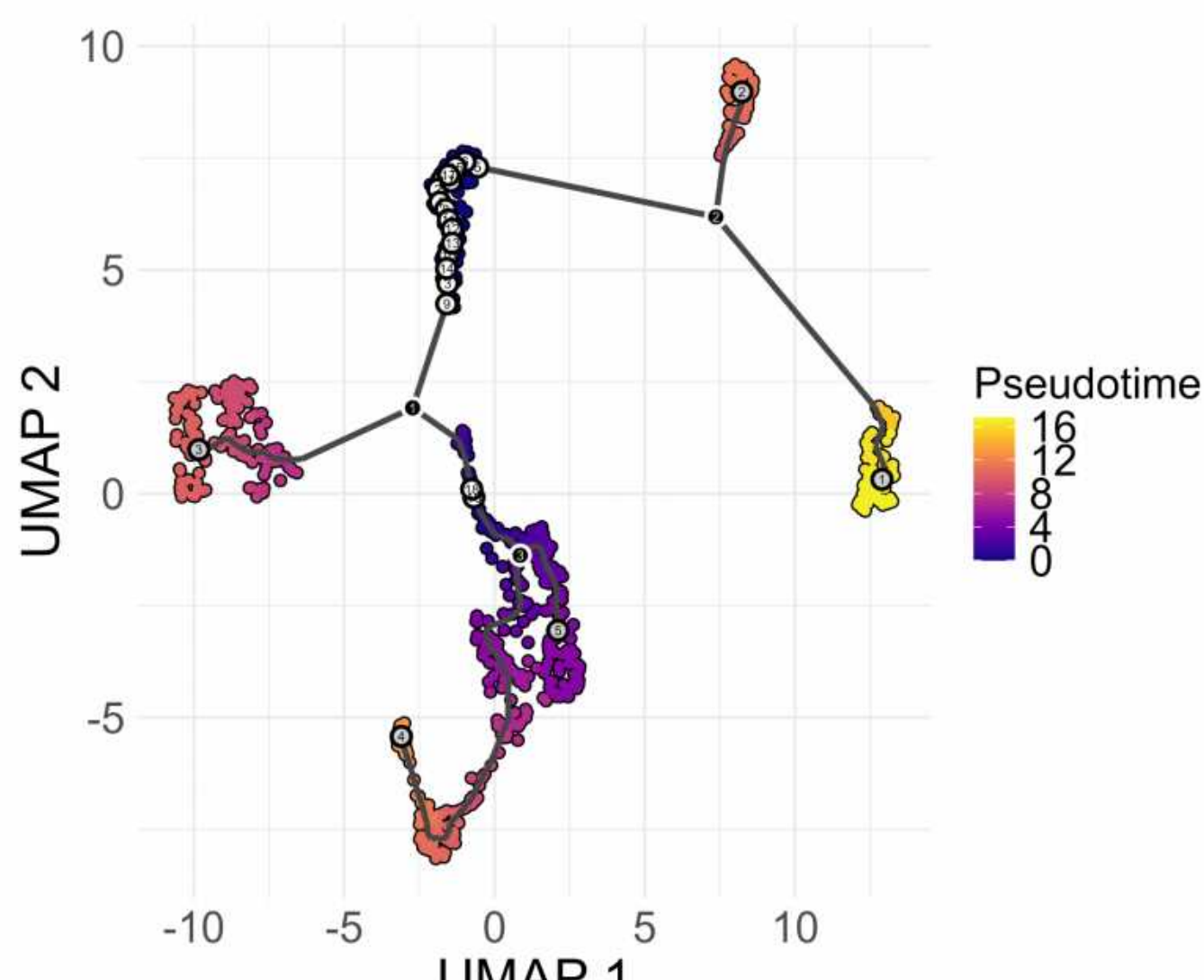

Supplement: S8 Fig — Focusing on comparing pseudotime results using data imputed by other methods. (PDF) [file pcbi.1014051.s008.pdf]
