# Supplementary material for: Prior-guided factorization for reliable imputation of scRNA-seq data
Source: PLoS Comput Biol. 2026 Mar 20;22(3):e1014051. doi: 10.1371/journal.pcbi.1014051 (PMC13004523; doi:10.1371/journal.pcbi.1014051)
Supplement: S2 Fig — Label sensitivity analysis during the imputation process across D1, shaded regions around the curves indicate variability. (PDF) [file pcbi.1014051.s002.pdf]

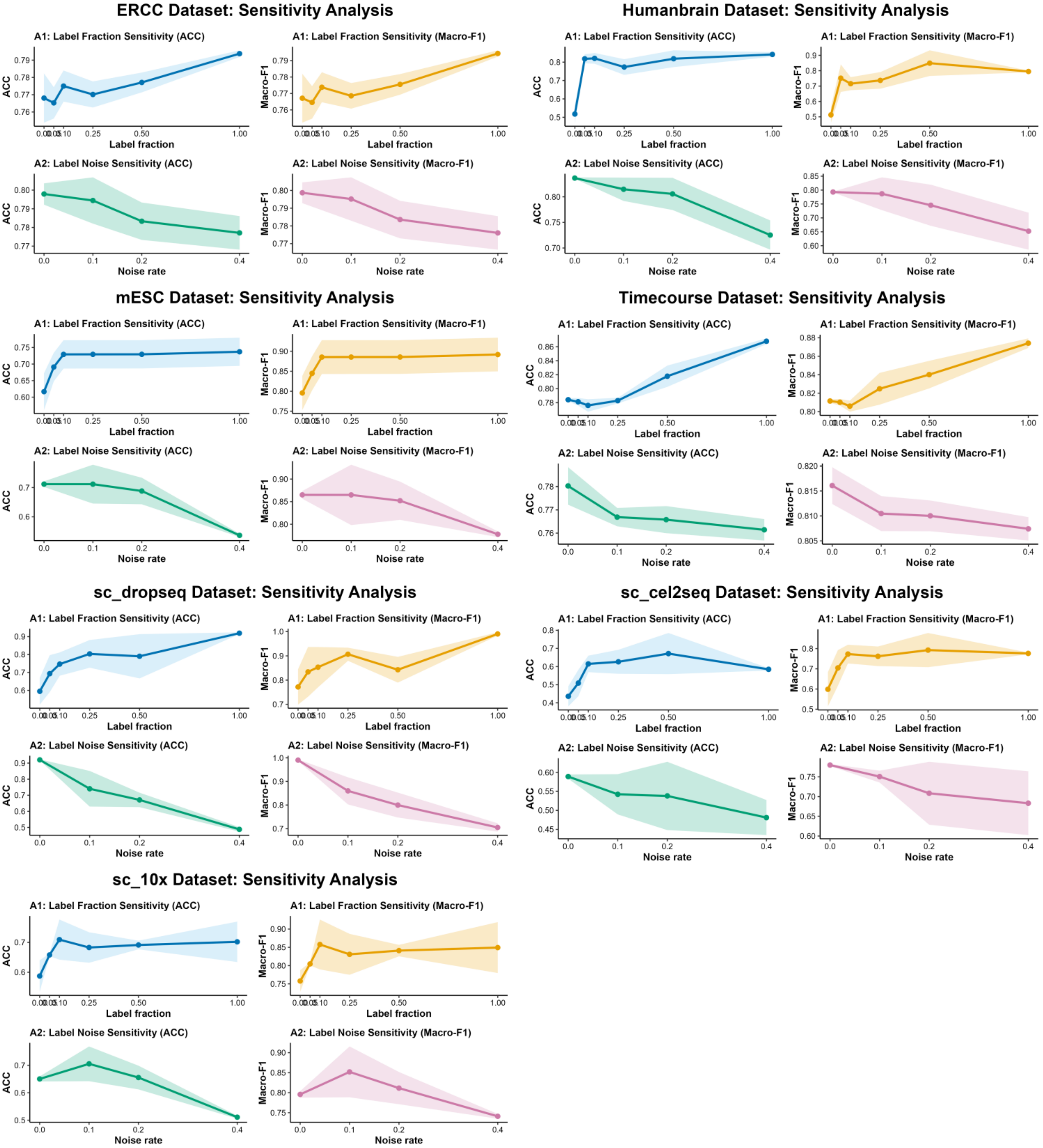
ERCC Dataset: Sensitivity Analysis
A1: Label Fraction Sensitivity (ACC)
A1: Label Fraction Sensitivity (Macro-F1)
A2: Label Noise Sensitivity (ACC)
A2: Label Noise Sensitivity (Macro-F1)
Humanbrain Dataset: Sensitivity Analysis
A1: Label Fraction Sensitivity (ACC)
A1: Label Fraction Sensitivity (Macro-F1)
A2: Label Noise Sensitivity (ACC)
A2: Label Noise Sensitivity (Macro-F1)
mESC Dataset: Sensitivity Analysis
A1: Label Fraction Sensitivity (ACC)
A1: Label Fraction Sensitivity (Macro-F1)
A2: Label Noise Sensitivity (ACC)
A2: Label Noise Sensitivity (Macro-F1)
Timecourse Dataset: Sensitivity Analysis
A1: Label Fraction Sensitivity (ACC)
A1: Label Fraction Sensitivity (Macro-F1)
A2: Label Noise Sensitivity (ACC)
A2: Label Noise Sensitivity (Macro-F1)
sc_dropseq Dataset: Sensitivity Analysis
A1: Label Fraction Sensitivity (ACC)
A1: Label Fraction Sensitivity (Macro-F1)
A2: Label Noise Sensitivity (ACC)
A2: Label Noise Sensitivity (Macro-F1)
sc_cel2seq Dataset: Sensitivity Analysis
A1: Label Fraction Sensitivity (ACC)
A1: Label Fraction Sensitivity (Macro-F1)
A2: Label Noise Sensitivity (ACC)
A2: Label Noise Sensitivity (Macro-F1)
sc_10x Dataset: Sensitivity Analysis
A1: Label Fraction Sensitivity (ACC)
A1: Label Fraction Sensitivity (Macro-F1)
A2: Label Noise Sensitivity (ACC)
A2: Label Noise Sensitivity (Macro-F1)
ACC
Macro-F1
Label fraction
Noise rate
