# Supplementary material for: Prior-guided factorization for reliable imputation of scRNA-seq data
Source: PLoS Comput Biol. 2026 Mar 20;22(3):e1014051. doi: 10.1371/journal.pcbi.1014051 (PMC13004523; doi:10.1371/journal.pcbi.1014051)

Human Brain (Raw vs Imputed) AutoImpute

Values are per-gene z-scores of AverageExpression grouped by celltype

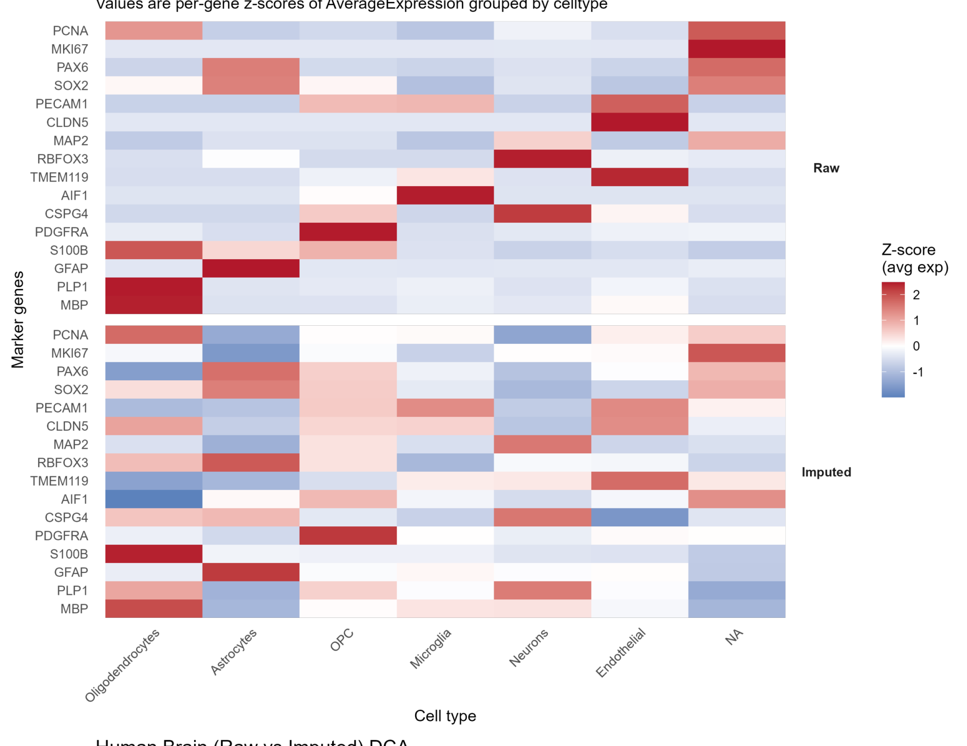

Human Brain (Raw vs Imputed) DCA

Values are per-gene z-scores of AverageExpression grouped by celltype

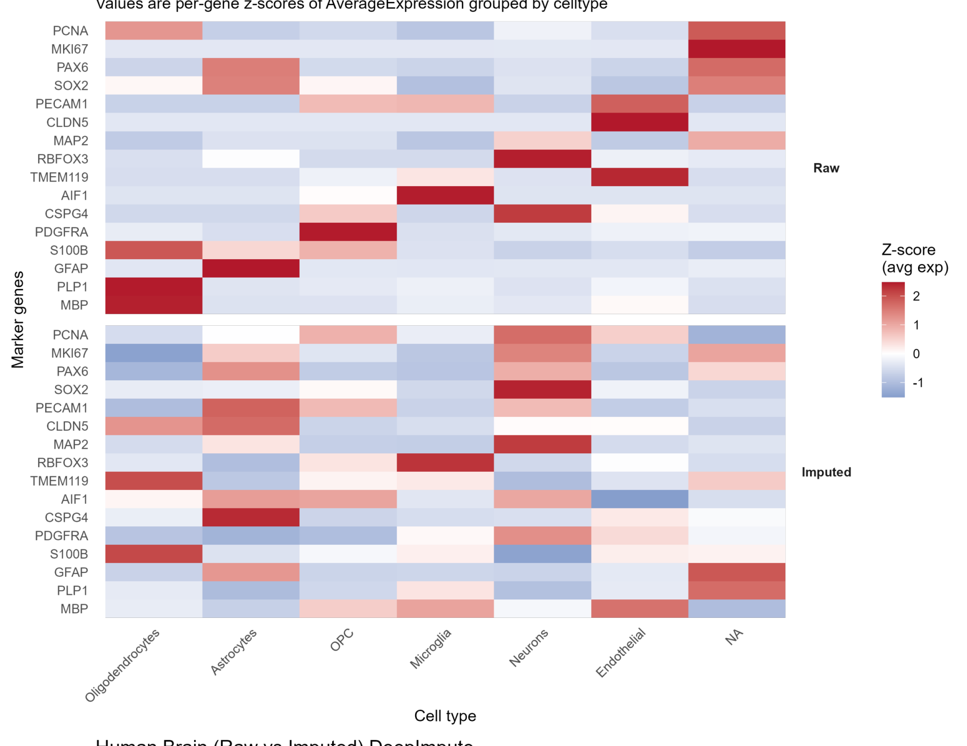

Human Brain (Raw vs Imputed) DeepImpute

Values are per-gene z-scores of AverageExpression grouped by celltype

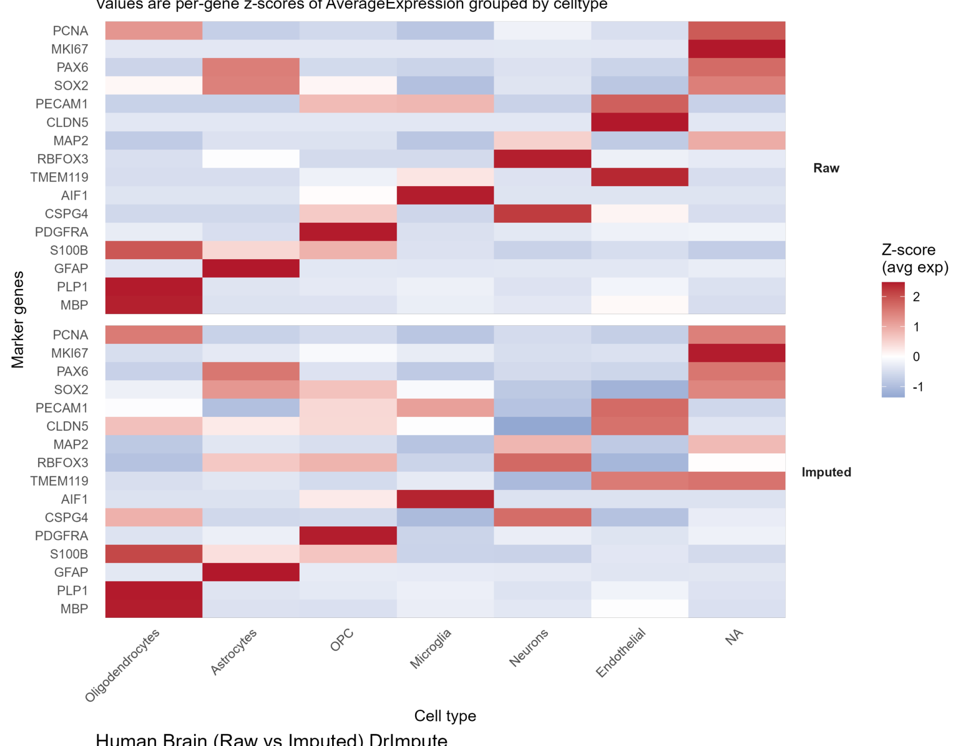

Human Brain (Raw vs Imputed) DrImpute

Values are per-gene z-scores of AverageExpression grouped by celltype

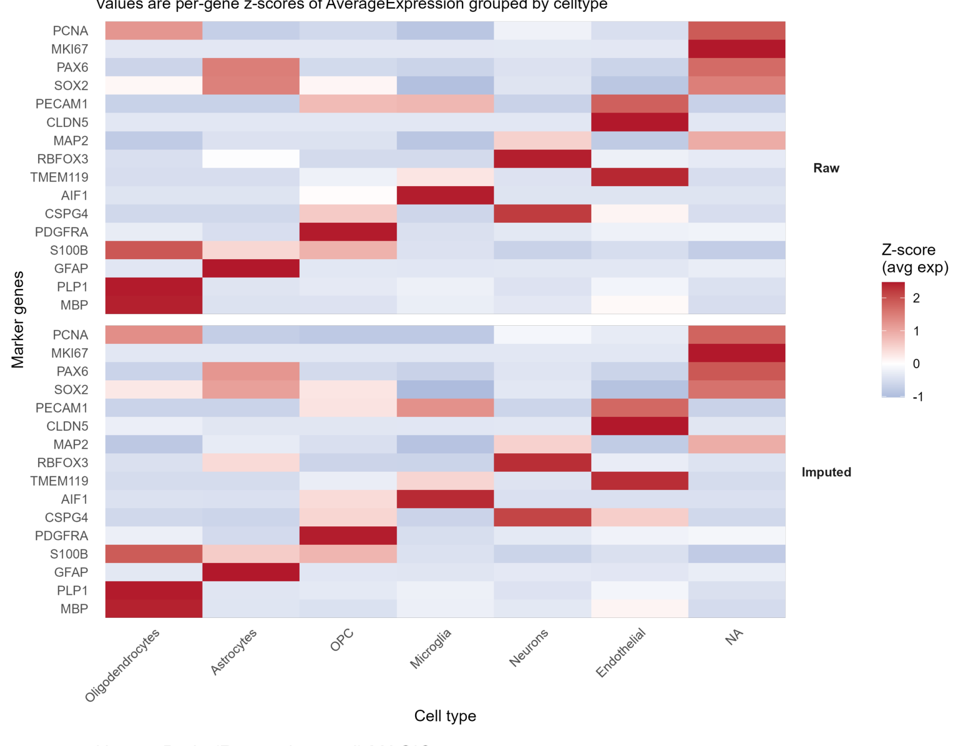

Human Brain (Raw vs Imputed) MAGIC

Values are per-gene z-scores of AverageExpression grouped by celltype

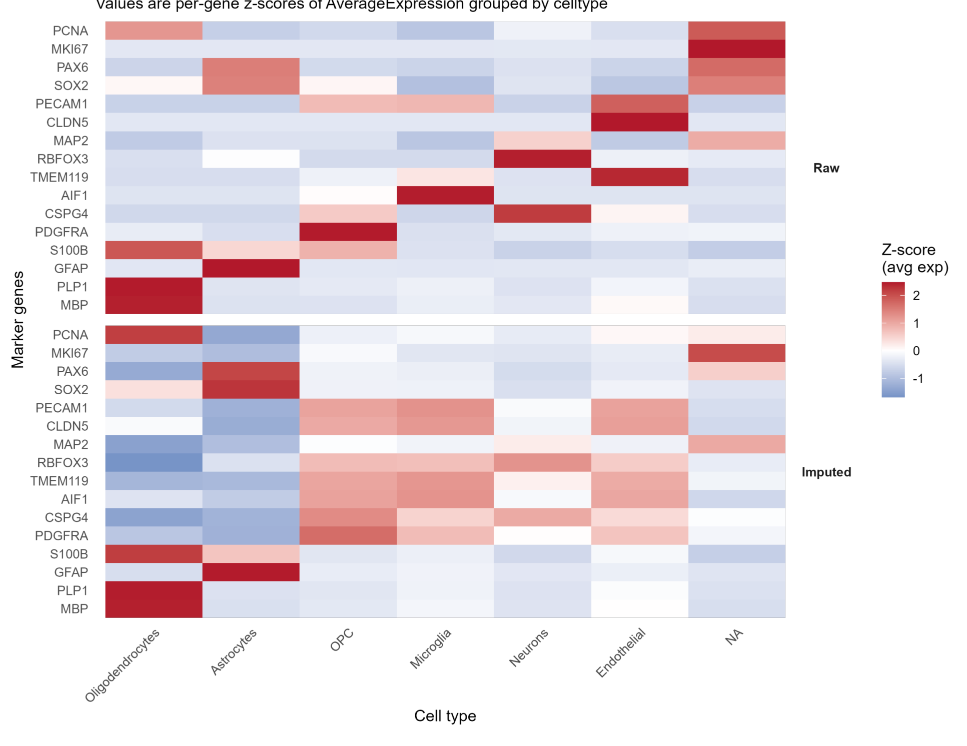

Human Brain (Raw vs Imputed) SAVER

Values are per-gene z-scores of AverageExpression grouped by celltype

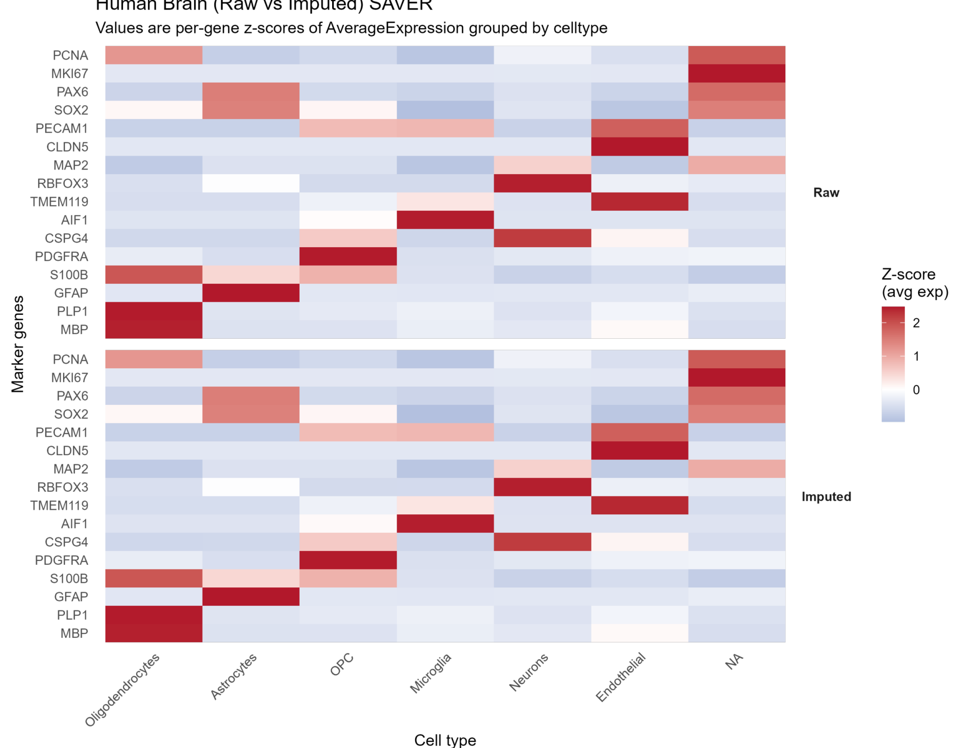

Human Brain (Raw vs Imputed) scGAIN

Values are per-gene z-scores of AverageExpression grouped by celltype

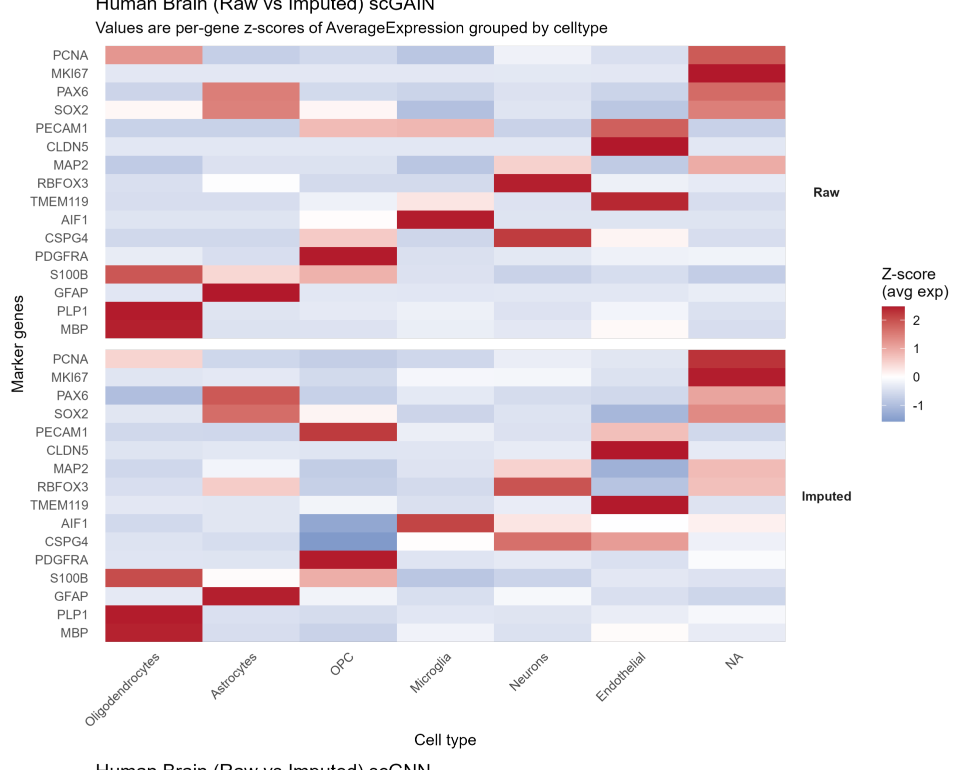

Human Brain (Raw vs Imputed) scGNN

Values are per-gene z-scores of AverageExpression grouped by celltype

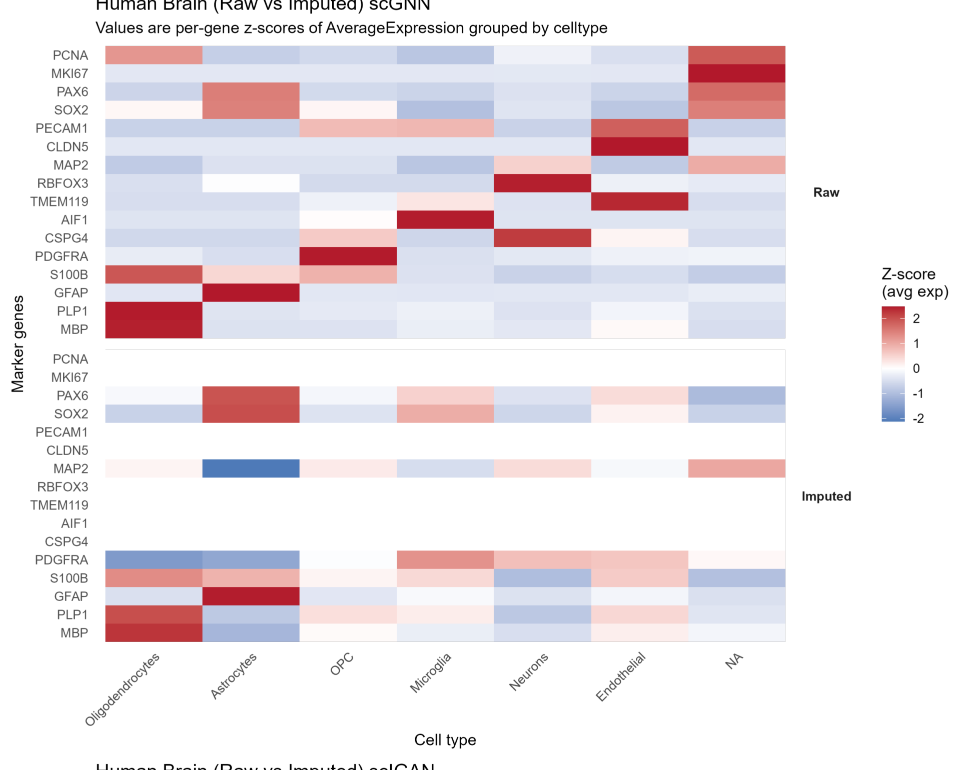

Human Brain (Raw vs Imputed) scIGAN

Values are per-gene z-scores of AverageExpression grouped by celltype

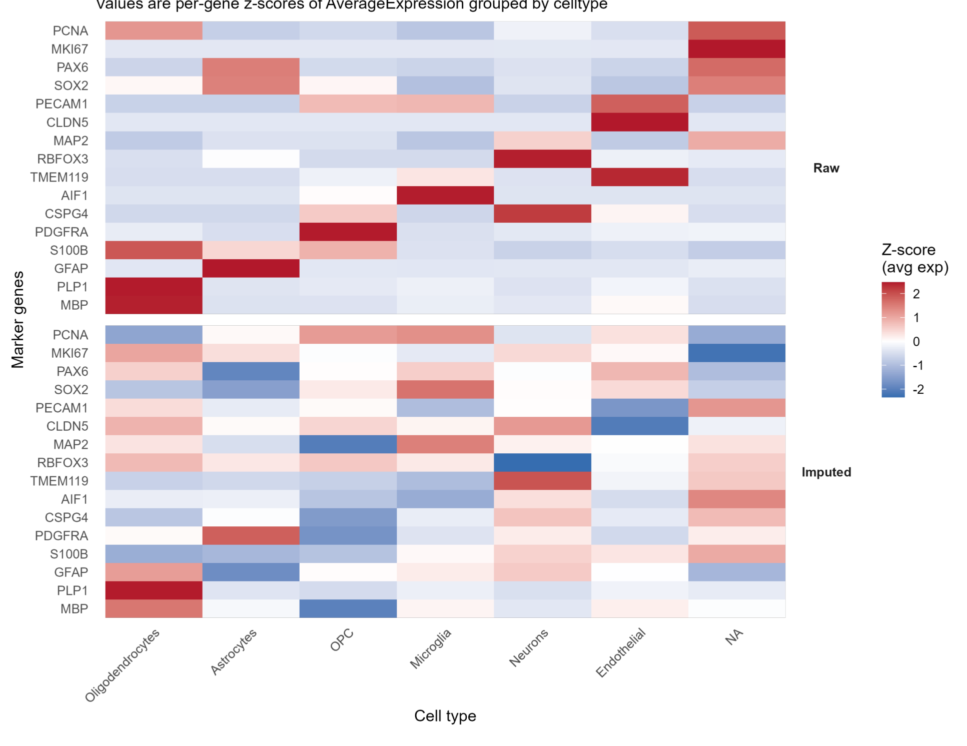

Human Brain (Raw vs Imputed) scImpute

Values are per-gene z-scores of AverageExpression grouped by celltype

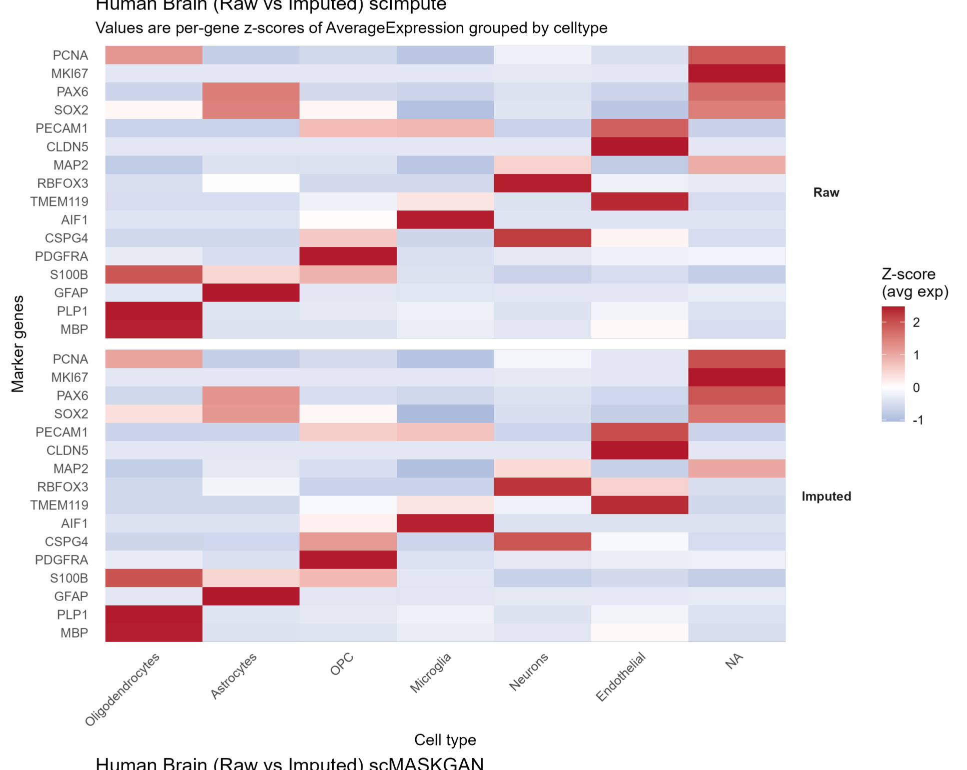

Human Brain (Raw vs Imputed) scMASKGAN

Values are per-gene z-scores of AverageExpression grouped by celltype

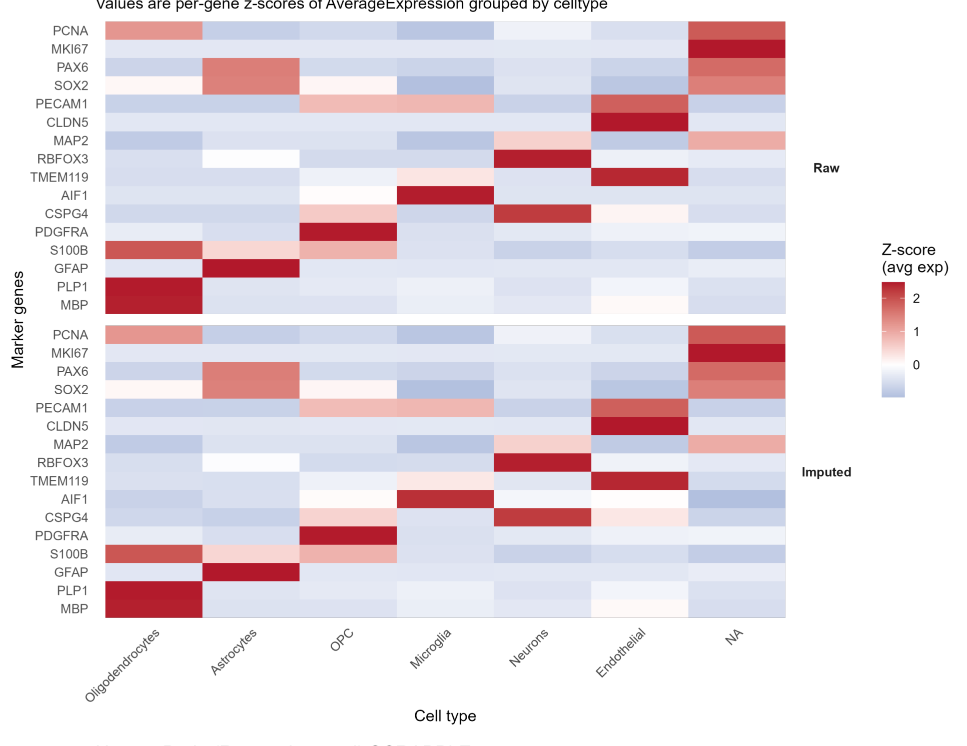

Human Brain (Raw vs Imputed) SCRABBLE

Values are per-gene z-scores of AverageExpression grouped by celltype

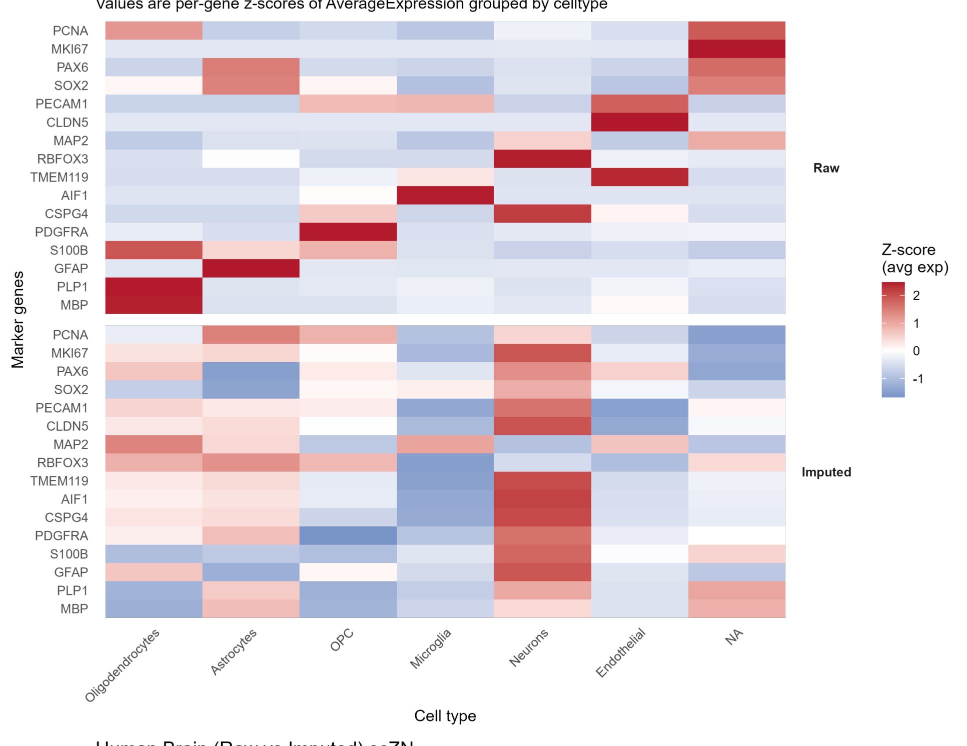

Human Brain (Raw vs Imputed) scZN

Values are per-gene z-scores of AverageExpression grouped by celltype

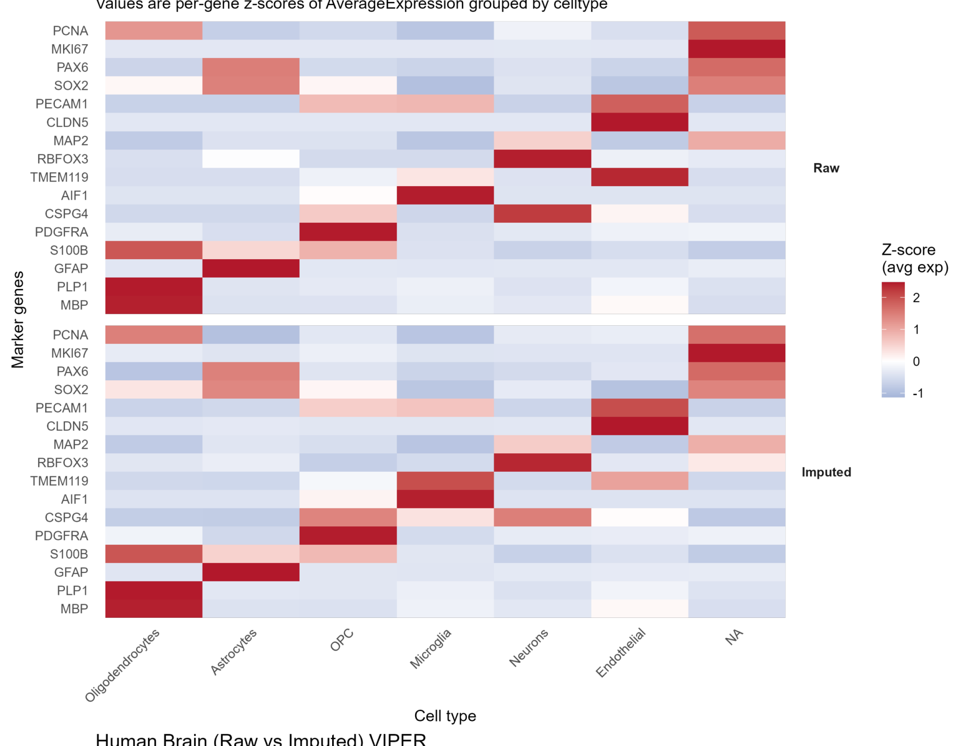

Human Brain (Raw vs Imputed) VIPER

Values are per-gene z-scores of AverageExpression grouped by celltype

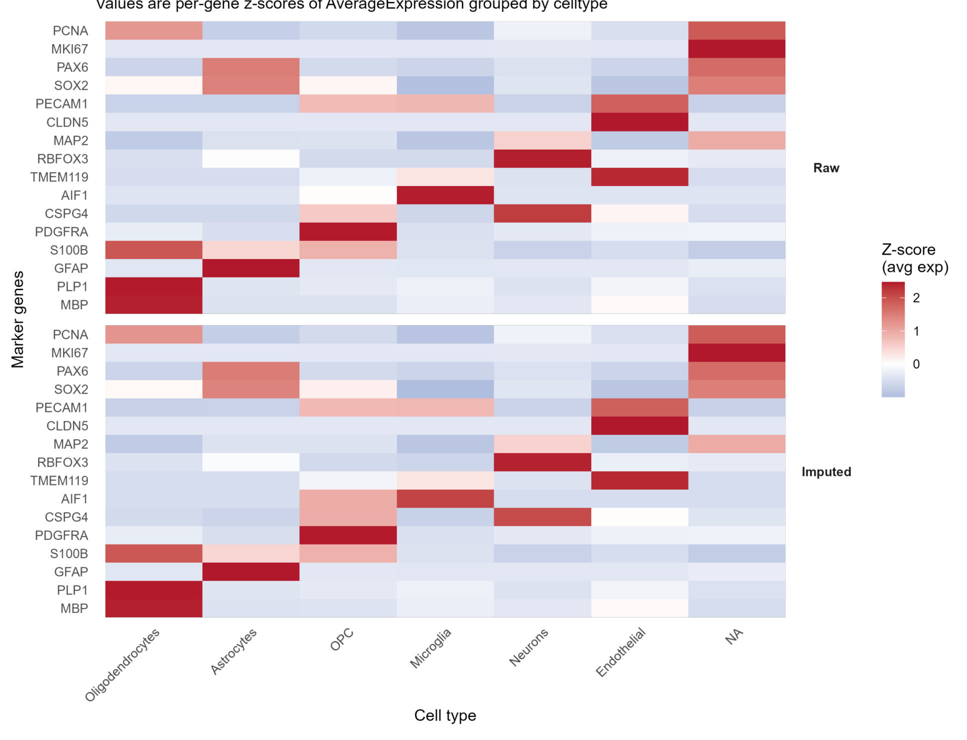

Supplement: S3 Fig — This figure shows the heatmap expression of labeled genes after imputation on a human dataset using multiple methods. (PDF) [file pcbi.1014051.s003.pdf]
