# Supplementary material for: Prior-guided factorization for reliable imputation of scRNA-seq data
Source: PLoS Comput Biol. 2026 Mar 20;22(3):e1014051. doi: 10.1371/journal.pcbi.1014051 (PMC13004523; doi:10.1371/journal.pcbi.1014051)
Supplement: S5 Fig — Comparison of the significance of GAD1 in Neurons and OPC datasets after imputation using 14 methods. (PDF) [file pcbi.1014051.s005.pdf]

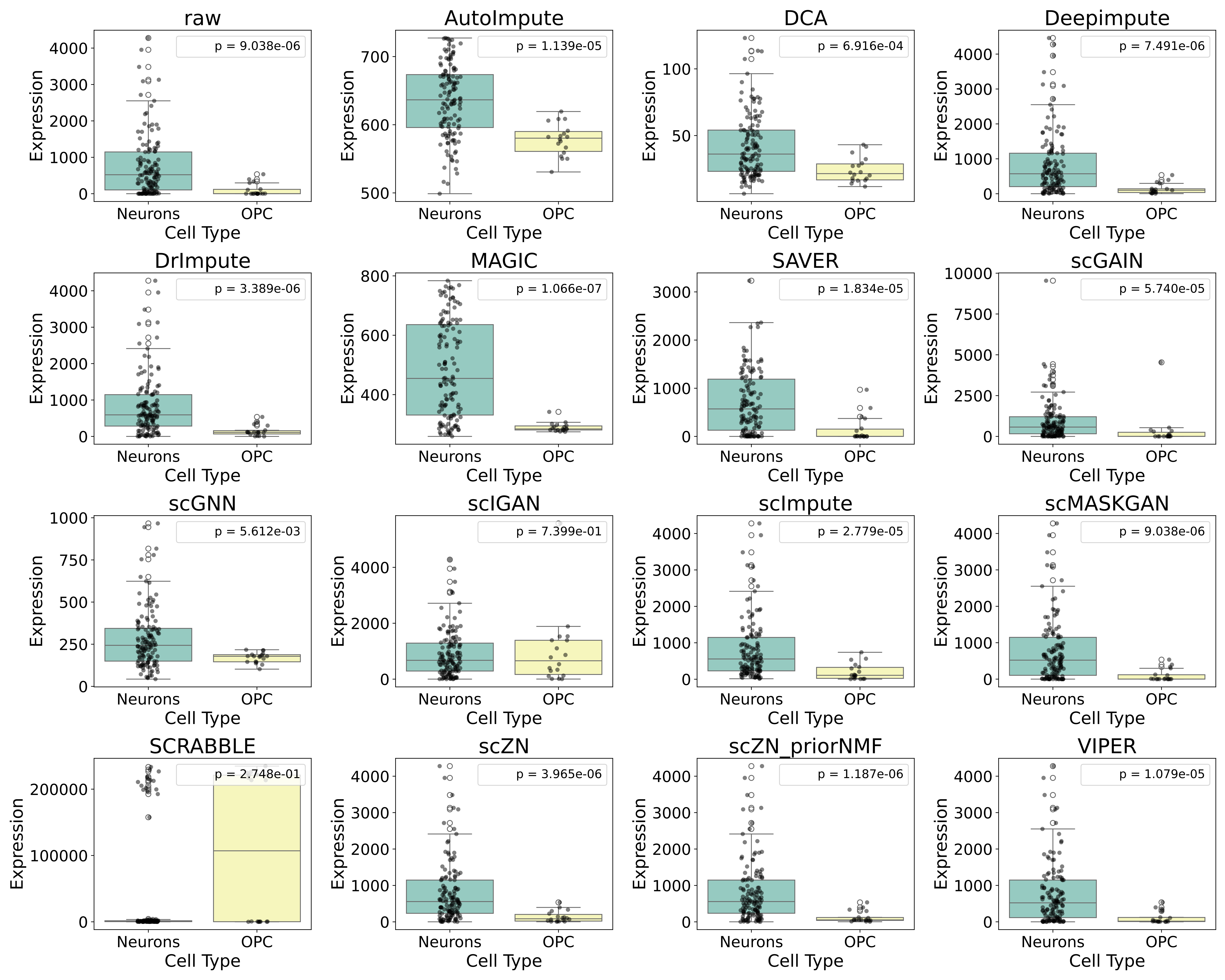

raw
p = 9.038e-06
AutoImpute
p = 1.139e-05
DCA
p = 6.916e-04
Deepimpute
p = 7.491e-06
DrImpute
p = 3.389e-06
MAGIC
p = 1.066e-07
SAVER
p = 1.834e-05
scGAIN
p = 5.740e-05
scGNN
p = 5.612e-03
scIGAN
p = 7.399e-01
scImpute
p = 2.779e-05
scMASKGAN
p = 9.038e-06
SCRABBLE
p = 2.748e-01
scZN
p = 3.965e-06
scZN_priorNMF
p = 1.187e-06
VIPER
p = 1.079e-05
Expression
Cell Type
Neurons
OPC
