# Supplementary material for: Prior-guided factorization for reliable imputation of scRNA-seq data
Source: PLoS Comput Biol. 2026 Mar 20;22(3):e1014051. doi: 10.1371/journal.pcbi.1014051 (PMC13004523; doi:10.1371/journal.pcbi.1014051)
Supplement: S9 Fig — Focusing on fitting the spliced/unspliced dynamics of the genes in the Fig 5c heatmap and supplementing with corresponding UMAP plots of velocity and gene expression. (PDF) [file pcbi.1014051.s009.pdf]

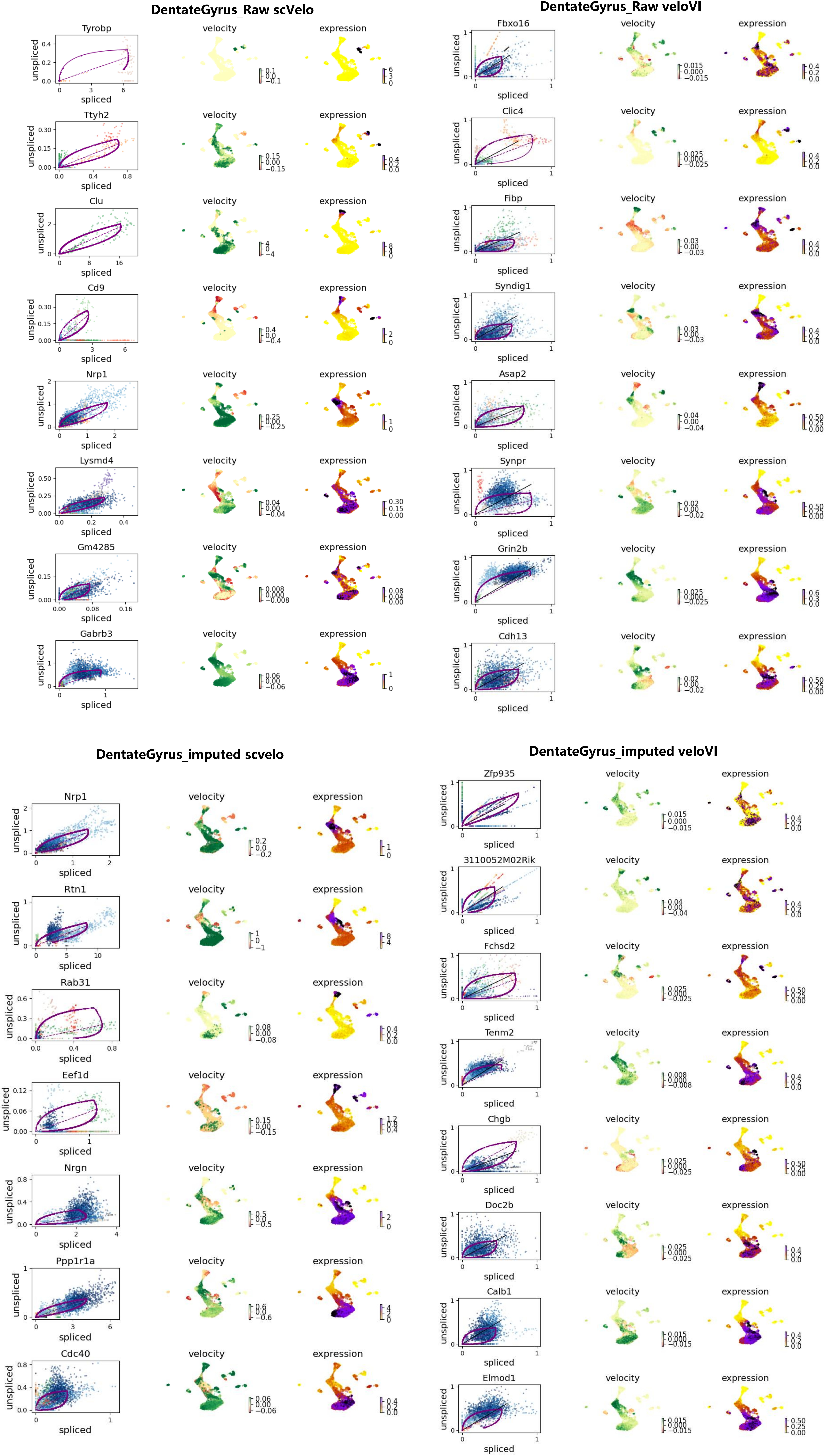
DentateGyrus_Raw scVelo
Tyrobp
Ttyh2
Clu
Cd9
Nrp1
Lysmd4
Gm4285
Gabrb3
DentateGyrus_Raw veloVI
Fbxo16
Clic4
Fibp
Syndig1
Asap2
Synpr
Grin2b
Cdh13
DentateGyrus_imputed scvelo
Nrp1
Rtn1
Rab31
Eef1d
Nrgn
Ppp1r1a
Cdc40
DentateGyrus_imputed veloVI
Zfp935
3110052M02Rik
Fchsd2
Tenm2
Chgb
Doc2b
Calb1
Elmod1
unspliced
spliced
velocity
expression
